# Angucycline Glycosides from Mangrove-Derived *Streptomyces* *diastaticus* subsp. SCSIO GJ056

**DOI:** 10.3390/md16060185

**Published:** 2018-05-28

**Authors:** Chun Gui, Yena Liu, Zhenbin Zhou, Shanwen Zhang, Yunfeng Hu, Yu-Cheng Gu, Hongbo Huang, Jianhua Ju

**Affiliations:** 1 CAS Key Laboratory of Tropical Marine Bio-resources and Ecology, Guangdong Key Laboratory of Marine Materia Medica, RNAM Center for Marine Microbiology, South China Sea Institute of Oceanology, Chinese Academy of Sciences, 164 West Xingang Road, Guangzhou 510301, China; guichun1988@sina.com (C.G.); zzb1881396@163.com (Z.Z.); sherry920111@163.com (S.Z.); yunfeng.hu@scsio.ac.cn (Y.H.); 2 University of Chinese Academy of Sciences, 19 Yuquan Road, Beijing 110039, China; 3 State Key Laboratory of Oncology in South China, Collaborative Innovation Center for Cancer Medicine, Sun Yat-Sen University Cancer Center, Guangzhou 510060, China; LIUYN@sysucc.org.cn; 4 Syngenta Jealott’s Hill International Research Centre, Bracknell, Berkshire RG42 6EY, UK; yucheng.gu@syngenta.com

**Keywords:** mangrove-derived *Streptomyces*, angucycline, urdamycin

## Abstract

Nine new angucycline glycosides designated urdamycins N1–N9 (**1**–**9**), together with two known congener urdamycins A (**10**) and B (**11**), were obtained from a mangrove-derived *Streptomyces* *diastaticus* subsp. SCSIO GJ056. The structures of new compounds were elucidated on the basis of extensive spectroscopic data analysis. The absolute configurations of **6**–**9** were assigned by electronic circular dichroism calculation method. Urdamycins N7 (**7**) and N8 (**8**) represent the first naturally occurring (5*R*, 6*R*)-angucycline glycosides, which are diastereomers of urdamycins N6 (**6**) and N9 (**9**), respectively.

## 1. Introduction

The angucycline group members are type II polyketide derived metabolites obtained exclusively from actinomycetes [1,2,3]. Angucycline compounds exhibited various bioactivities, including antitumor, cytostatic, enzyme inhibition, antibacterial, antiviral, and inhibition of platelet aggregation function [4,5,6,7].

During the course of searching for novel anti-infective and antitumor agents from the marine environment, we found that the chemical profile of strain SCSIO GJ056 cultivated in AM2 medium revealed an array of secondary metabolites showing typical UV/VIS absorptions, which were similar to those of angucyclines/anthracyclines. Subsequent solvent extraction and isolation procedures led to the purification and structure elucidation of nine new angucycline glycosides, named urdamycins N1–N9 (**1**–**9**), together with two known urdamycins A (**10**) and B (**11**). Urdamycins N7 (**7**) and N8 (**8**) represent the first naturally occurring (5*R*, 6*R*)-angucycline glycosides. Herein, we report the fermentation, isolation, and structure elucidation of these compounds.

## 2. Results and Discussion

**Figure 1:**
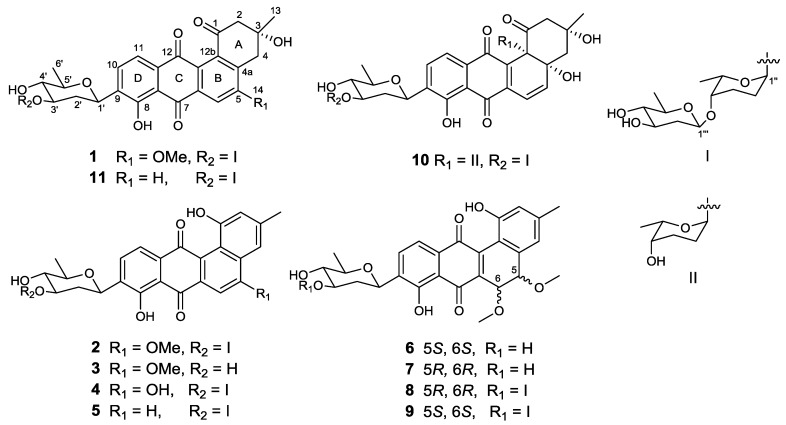
Structures of compounds **1**–**11**.

The strain SCSIO GJ056 was fermented (15 L) and the fermentation broth was extracted with butanone. The extract was subjected to repetitive silica gel column chromatography, followed by preparative HPLC purification to yield compounds **1**–**11** (Figure 1). The known urdamycins A (**10**) and B (**11**) were identified by comparisons of MS, ^1^H, and ^13^C NMR spectroscopic data with those previously reported [8].

**Figure 2:**
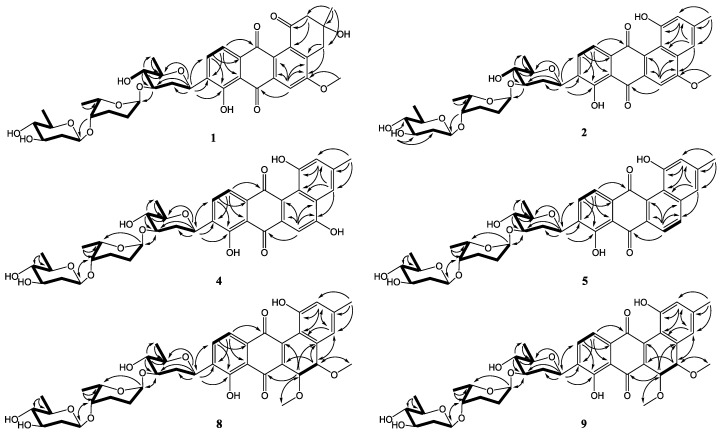

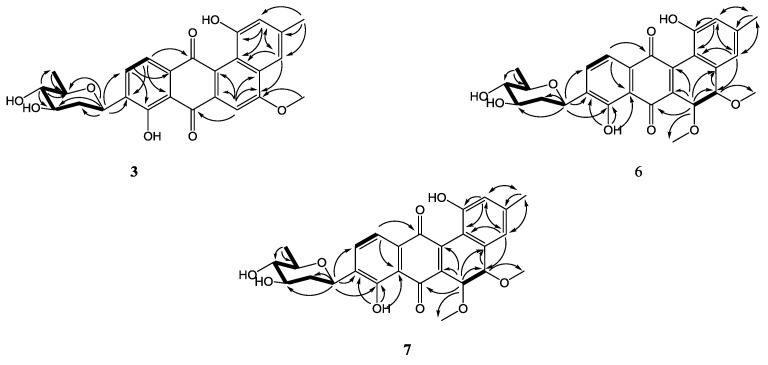
COSY (**bold**) and selected HMBC (**arrow**) correlations for **1**–**9**.

Compound **1** was obtained as a yellowish powder. Its molecular formula was determined to be C_38_H_46_O_14_ on the basis of HRESIMS peak at *m*/*z* 725.2834 [M − H]^−^, indicating 16 degrees of unsaturation. The ^13^C and DEPT NMR spectra of **1** displayed 38 carbon resonances, including five methyls, six methylenes, 14 methines, and 13 nonprotonated carbons. The ^1^H NMR spectrum showed one chelated hydroxy group signal at *δ*_H_ 12.55 (1H, br s, 8-OH), a pair of *ortho*-coupled aromatic proton signals at *δ*_H_ 7.81 (d, 7.8 Hz, H-10) and 7.59 (d, 7.8 Hz, H-11), and a singlet aromatic proton signal at *δ*_H_ 7.65 (s, H-6). The HMBC correlations (Figure 2) from H-6 to C-4a, C-5, C-7, and C-12a, from H-11 to C-7a, C-9, and C-12, and from H-10 to C-8, C-9, and C-11a confirmed the existence of the anthraquinone skeleton (rings B, C, and D). Further HMBC correlations of H_2_-2/C-1, C-12b, C-4; H_2_-4/C-2, C-4a, C-12b; and H_3_-13/C-2, C-3, C-4 allowed the assignment of the angular ring (ring A) with a methyl group (CH_3_-13) substitution at C-3. A methoxy group (OCH_3_-14) attached at C-5 in ring B was deduced by the HMBC correlation of H_3_-14/C-5. A hydroxy group linked at C-3 in ring A was inferred based on the ^13^C NMR chemical shift at *δ*_C_ 71.8. The absolute configuration of C-3 was tentatively deduced to be *R*, which was identical with that of urdamycinone B and N05WA963D in light of the similar ^13^C NMR resonances of C-3 and CH_3_-13, as well as the similar biosynthetic pathway [8,9].

**Figure 3:**
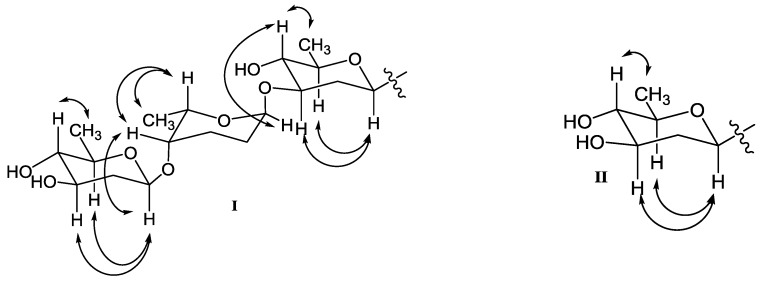
Key NOESY correlations of *β*-olivose-(1→4)-*α*-rhodinose-(1→3)-*β*-olivose in **1**, **2**, **4**, **5**, **8**, and **9** (**I**), and *β*-olivose in **3**, **6** and **7** (**II**).

In addition, three anomeric methine signals at *δ*_H_ 4.82 and *δ*_C_ 71.1 (CH-1′), *δ*_H_ 4.96 and *δ*_C_ 97.4 (CH-1″), and *δ*_H_ 4.45 and *δ*_C_ 101.5 (CH-1′′′), together with three doublet methyl signals at *δ*_H_ 1.37 (CH_3_-6′), *δ*_H_ 1.16 (CH_3_-6′′), and *δ*_H_ 1.23 (CH_3_-6′′′) revealed the presence of a trisaccharide moiety consisting of three deoxy sugars in **1**. The ^1^H-^1^H COSY spectrum allowed the full assignment of the sugar moieties from CH-1′ to CH_3_-6′, from CH-1′′ to CH_3_-6′′, and from CH-1′′′ to CH_3_-6′′′. The HMBC correlations of H-3′/C-1′′ and H-4′′/C-1′′′ confirmed the existence of a *β*-olivose-(1→4)-*α*-rhodinose-(1→3)-*β*-olivosyl unit. This trisaccharide was connected with the aglycone at C-9 based on the HMBC correlations of H-1′/C-9 and H-9/C-1′. The relative configuration of the trisaccharide moiety was deduced by ^1^H-^1^H coupling constants (Table 1) and NOE experiment (Figure 3). Detailed comparisons showed that the ^1^H and ^13^C NMR spectroscopic data of the sugar units were almost identical with those in urdamycin A [10,11]. Thus, the structure of **1** was determined and named urdamycin N1.

Compound **2**, isolated as a dark red powder, has the molecular formula of C_38_H_44_O_13_ on the basis of HRESIMS peak at *m/z* 707.2708 [M − H]^−^, showing 17 degrees of unsaturation and an 18 amu less than that of compound **1**. An obvious red shift on the UV-VIS spectrum of **2** relative to that of **1** indicated an additional conjugated system in **2**. The ^13^C and DEPT NMR data of **2** displayed 38 carbon signals attributable to five methyls, four methylenes, 16 methines, and 13 nonprotonated carbons. The ^1^H and HSQC NMR spectra suggested three singlet olefnic proton signals at *δ*_H_ 7.58 (H-4), *δ*_H_ 7.54 (H-6), and *δ*_H_ 6.99 (H-2), and a pair of *ortho*-coupled aromatic proton signals at *δ*_H_ 7.82 (d, 7.6 Hz, H-10) and 7.68 (d, 7.6 Hz, H-11). Comparing the ^1^H and ^13^C NMR spectroscopic data to those of **1** revealed that **2** possessed a similar core structure with that of **1**. The difference between **2** and **1** was the aromatization of ring A in **2**, which supported by the HMBC correlations from CH_3_-13 to C-2, C-3, and C-4, from H-2 to C-1, C-4, and C-12b, and from H-4 to C-2, C-4a, and C-12b. Compound **2** possessed the same trisaccharide moiety with **1** according to similar ^1^H and ^13^C NMR signals in aliphatic area. The structure of **2** was elucidated as shown in Figure 1 by detailed analysis of 2D NMR spectra data. 

Compound **3**, a dark green powder, was isolated as minor component from the extract. Its molecular formula of C_26_H_24_O_8_ was determined by the HRESIMS peak at *m*/*z* 463.1409 [M − H]^−^, indicating 15 degrees of unsaturation. Comprehensive analysis of its ^1^H and ^13^C NMR spectroscopic data revealed that **3** had the same aglycone with that of **2**. However, a set of ^1^H and ^13^C resonances ascribed to *β*-olivose-(1→4)-*α*-rhodinosyl moiety disappeared, indicating the absence of two sugar units in **3**. This is consistent with the HRESIMS data, which showing a C_12_H_20_O_5_ fragment loss relative to **2**. Therefore, the structure of **3** was established and named urdamycin N3.

Compound **4** was obtained as a dark green powder. Its molecular formula was determined to be C_37_H_42_O_13_ by the HRESIMS peak at *m/z* 693.2554 [M − H]^−^. The ^1^H and ^13^C NMR data of **4** were closely similar to those of **2**, except that the methoxy signals at *δ*_H_ 4.14, *δ*_C_ 56.6 in **2** were absent. The ^13^C NMR signal of C-5 shifted from *δ*_C_ 160.3 in **2** to *δ*_C_ 163.6 in **4**, indicating the OMe-5 in **2** was replaced by OH-5 in **4**. Compound **4** was named urdamycin N4.

Compound **5** was obtained as a red powder. The molecular formula of C_37_H_42_O_12_, as determined by HRESIMS, which was one oxygen atom less than that of **4**. The ^1^H and ^13^C NMR spectroscopic data were similar with those of **4**, except that two pairs of *ortho*-coupled aromatic signals were observed. Additionally, the ^13^C NMR signal at *δ*_C_ 163.6 for the oxygen-bearing aromatic C-5 in **4** was replaced by an aromatic methine signal at *δ*_C_ 135.4. Thus, the structure of **5** was determined as 5-demethoxy-urdamycin N2, designated as urdamycin N5.

**Figure 4:**
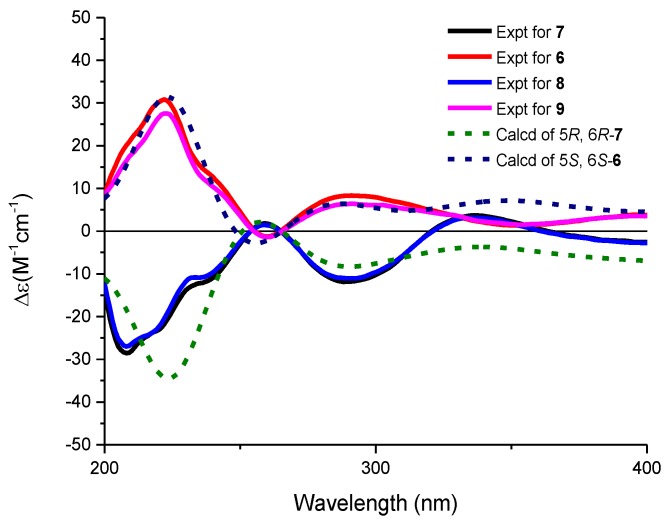
Experimental and calculated ECD spectra of **6**–**9** in methanol.

The molecular formulae of compounds **6** and **7** were determined both to be C_27_H_28_O_9_ by HRESIMS, indicating 14 degrees of unsaturation. The ^1^H and ^13^C NMR spectroscopic data of **6** were similar with those of **3**, except that two aromatic carbon signals at *δ*_C_ 160.5 (C-5) and 100.0 (C-6) in **3** were replaced by two oxygen-bearing methine carbon signals at *δ*_C_ 78.1 (C-5) and 70.2 (C-6). Furthermore, two methoxys were attached at C-5 and C-6 based on the HMBC correlations of H_3_-14/C-5 and H_3_-15/C-6, respectively. Small coupling constants (2.8 Hz) between H-5 and H-6 revealed a *trans* configuration of H-5 and H-6, indicating an (5*R*, 6*R*) or (5*S*, 6*S*) configuration of **6**. To determine the absolute configurations of **6**, comparisons of the experimental and ECD spectra using a time-dependent density functional theory (TDDFT) were employed. Comparison of the experimental and calculated CD spectra (Figure 4) established the absolute configuration as (5*S*, 6*S*) for **6**, which were the same as those of PMO70747, PD116740, and TAN-1085 [12,13,14,15,16,17]. Compound **7** possessed the same planar structure with that of **6**, as deduced by the COSY and HMBC spectra (Figure 2). However, the experimental and calculated CD spectra of **7** showed cotton effects totally opposite to those of **6**, respectively, inferring the (5*R*, 6*R*) configuration for **7** (Figure 4). Compounds **6** and **7** were named urdamycins N6 and N7, respectively.

Compounds **8** and **9** were isolated as red powder, both had the same molecular formula of C_39_H_48_O_14_ as determined by HRESIMS. The ^1^H and ^13^C NMR spectroscopic data of **8** and **9** resembled those of **2**, except that two aromatic carbon signals at *δ*_C_ 160.3 (C-5) and 99.8 (C-6) in **2** were replaced by two oxygen-bearing methine carbon signals at *δ*_C_ 80.0 (C-5) and 67.4 (C-6) in **8**, and at *δ*_C_ 78.1 (C-5) and 70.2 (C-6) in **9**. Two methoxys were attached at C-5 and C-6 in **8** and **9** based on the HMBC correlations of H_3_-14/C-5 and H_3_-15/C-6, respectively. Small coupling constants of H5/H6 revealed the both *trans* configurations in compounds **8** and **9**. The absolute configurations were determined to be (5*R*, 6*R*) for **8** and (5*S*, 6*S*) for **9** by comparing their CD curve to those of **7** and **6**, respectively (Figure 4). Finally, compounds **8** and **9** were elucidated and named urdamycin N8 and urdamycin N9, respectively. Compounds **7** and **8** represent the first naturally occurring (5*R*, 6*R*)-angucycline metabolite.

## 3. Experimental Section

### 3.1. General Experimental Procedures

Column chromatography (CC) was performed using silica gel (100–200 mesh; Jiangpeng Silica gel development, Inc., Shandong, China). Thin layer chromatography (TLC) was conducted with precoated glass plates (0.1–0.2 mm; silica gel GF_254_, 10–40 nm, Jiangpeng, China). HPLC analyses were performed with a 1260 infinity system (Agilent, Santa Clara, CA, USA) using a Phenomenex Prodigy ODS (2) column (150 × 4.6 mm, 5 μm; USA). Semi-preparative HPLC were performed with a Primaide 1110 solvent delivery module equipped with a 1430 photodiode array detector (Hitachi, Tokyo, Japan), using a YMC-Pack ODS-A column (250 mm × 10 mm, 5 μm; YMC Co., Ltd., Kyoto, Japan). UV spectra were recorded on a U-2910 spectrometer (Shimadzu, Kyoto, Japan); IR spectra were obtained on an IRAffinity-1 spectrophotometer (Shimadzu, Kyoto, Japan). CD spectra were measured on a Chirascan circular dichroism spectrometer (Applied Photophysics, Leatherhead, UK). High-resolution mass spectral data were obtained on a MaXis Q-TOF mass spectrometer (Bruker, Billerica, MA, USA). Optical rotations were recorded with an MCP-500 polarimeter (Anton Paar, Graz, Austria). NMR spectra were recorded on a Bruker Avance 500 or a Bruker Avance 700 spectrometer (Bruker, Billerica, MA, USA). Carbon signals and the residual proton signals of DMSO-*d_6_* (*δ*_C_ 39.52 and *δ*_H_ 2.50), CD_3_OD (*δ*_C_ 49.0 and *δ*_H_ 4.87), and CDCl_3_ (*δ*_C_ 77.16 and *δ*_H_ 7.26) were used for calibration. Coupling constants (*J*) are given in Hz.

### 3.2. Bacterial Materials

Strain SCSIO GJ056 was isolated from a mangrove-derived sediment sample collected in Yalong bay, China. It was identified as *Streptomyces diastaticus* subsp. on the basis of morphological characteristics and 16S rRNA sequence analysis by comparisons with other sequences in the GenBank database. The DNA sequence has been deposited in GenBank (accession no. MH368281). The strain was preserved at the RNAM Center for Marine Microbiology, South China Sea Institute of Oceanology, Chinese Academy of Sciences and also at the China General Microbiological Culture Collection Center (CGMCC, Beijing, China), CGMCC No. 13648.

### 3.3. Fermentation, Extraction, and Isolation of the Compounds

A portion of spore and mycelium mixture of SCSIO GJ056 grown on ISP4 medium agar plates were inoculated into 50 mL modified AM2 medium (0.5% soybean flour, 0.5% soluble starch, 0.2% yeast extract, 0.2% peptone, 2.0% glucose, 0.05% KH_2_PO_4_, 0.05% MgSO_4_·7H_2_O, 0.4% NaCl, 0.2% CaCO_3_, 3.0% sea salt (Guangdong Province Salt Industry Group, Guangzhou, China), pH 7.2 before sterilization) in 250 mL Erlenmeyer flasks, and were incubated at 28 °C on a rotary shaker at 200 rpm for 1.5 days as the seed culture. Then the 50 mL of culture solution was transferred into a 1 L Erlenmeyer flask and then incubated at 28 °C, 200 rpm for seven days. On the seventh day, the entire culture broth (15 L) was harvested and centrifuged to yield the mycelial cake and liquid broth. The liquid broth was extracted with butanone for three times, and the mycelial cake was extracted using 1 L of acetone for three times. The combined organic layers were dried under vacuum to yield a residue. The residue was subjected to silica gel CC using gradient elution with CHCl_3_ and MeOH mixtures (100:0, 99:1, 97:3, 95:5, 90:10, 80:20, and 50:50, *v*/*v*) to give seven fractions (Fr.A1–Fr.A7). Fr.A1 and Fr.A2 were combined after HPLC analysis and purified by silica gel CC eluting with petroleum ether and EtOAc mixtures (100:0, 90:10, 80:20, 70:30, 60:40, 40:60, 20:80, 0:100, *v*/*v*) to give eight fractions (Fr.B1–Fr.B8). Fr.B4 was subjected to Sephadex LH-20 CC eluted with CHCl_3_/MeOH (1:1) to obtain **1** (6 mg). Fr.B5 and Fr.B6 were combined and further purified by semi-preparative HPLC with an ODS column to afford **10** (220 mg) at *t*_R_ 26 min and **11** (70 mg) at *t*_R_ 30 min. Fractions B1 and B2 were combined and subjected to preparative TLC using CHCl_3_/MeOH (92:8) to obtain six sub-fractions (Fr.C1–Fr.C6). Fr.C1 was purified by preparative HPLC with an ODS column eluted with CH_3_CN/H_2_O (30:70 to 100:0 over 28 min, then hold 7 min, 9 mL/min) to afford **7** (0.6 mg) and **6** (0.6 mg) at *t*_R_ 13.5 min, 14.5 min, respectively. Fr.C2 was purified by preparative HPLC with an ODS column eluted with CH_3_CN/H_2_O (30:70 to 100:0 over 28 min, then hold 7 min, 9 mL/min) to afford **8** (0.7 mg), **9** (0.6 mg), and **5** (0.6 mg) at *t*_R_ 15.5 min, 16.5 min, and 26 min, respectively. Fr.C3 was purified by preparative HPLC with an ODS column eluted with CH_3_CN/H_2_O (30:70 to 100:0 over 28 min, then hold 7 min, 9 mL/min) to afford **2** (8 mg) and **3** (7 mg) at *t*_R_ 32 min, 32.5 min, respectively. Fr.C6 was purified by preparative HPLC eluted with CH_3_CN/H_2_O (30:70 to 100:0 over 28 min, then held for 7 min, 9 mL/min) to afford **4** (8 mg) at *t*_R_ 25 min.

### 3.4. Spectral Data

*Urdamycin N1* (**1**). Dark red powder; [α${]}_{D}^{20}$ + 24 (*c* 0.05, CDCl_3_); UV (CDCl_3_) *λ*_max_ (log *ε*) 240 (4.06), 285 (4.27), 411 (3.69); IR *ν_max_* 3391, 2932, 1705, 1667, 1632, 1582, 1492, 1364, 1279, 1061, 1011, 754 cm^−1^; ^1^H NMR (500 MHz, CDCl_3_/CD_3_OD) and ^13^C NMR (125 MHz, CDCl_3_/CD_3_OD) data, Table 1; (-)HR-ESI-MS *m*/*z* 725.2834 ([M − H]^−^, calcd for C_38_H_45_O_14_, 725.2815).

*Urdamycin N2* (**2**). Dark green powder; [α${]}_{D}^{20}$ + 235 (*c* 0.06, CDCl_3_); UV (CDCl_3_) *λ*_max_ (log *ε*) 241 (4.62), 263 (4.55), 324 (4.71), 434 (4.17), IR *ν_max_* 3379, 2926, 1631, 1504, 1435, 1300, 1061, 1011, 758 cm^−1^; ^1^H NMR (700 MHz, DMSO-*d_6_*) and ^13^C NMR (176 MHz, DMSO-*d_6_*) data, Table 1; (-)HR-ESI-MS *m/z* 707.2708 ([M − H]^−^, calcd for C_38_H_43_O_13_, 707.2709).

*Urdamycin N3* (**3**). Dark green powder; [α${]}_{D}^{20}$ − 430 (*c* 0.04, CDCl_3_); UV (CDCl_3_) *λ*_max_ (log *ε*) 240 (4.48), 263 (4.39), 324 (4.55), 433 (4.02), IR *ν_max_* 3360, 2920, 1630, 1506, 1435, 1229, 1090, 1057, 772 cm^−1^; ^1^H NMR (700 MHz, DMSO-*d_6_*/CD_3_OD) and ^13^C NMR (176 MHz, DMSO-*d_6_*/CD_3_OD) data, Table 1; (-)HR-ESI-MS *m*/*z* 463.1409 ([M − H]^−^, calcd for C_26_H_23_O_8_, 463.1398).

*Urdamycin N4* (**4**). Dark green powder; [α${]}_{D}^{20}$ + 125 (*c* 0.06, CDCl_3_); UV (CDCl_3_) *λ*_max_ (log *ε*) 207 (3.92), 240 (4.16), 263 (4.05), 323 (4.18), 434 (3.67), IR *ν_max_* 3397, 2930, 1628, 1437, 1298, 1096, 1061, 1013, 852, 786 cm^−1^; ^1^H NMR (500 MHz, DMSO-*d_6_*/CD_3_OD) and ^13^C NMR (125MHz, DMSO-*d_6_*/CD_3_OD) data, Table 2; (-)HR-ESI-MS *m*/*z* 693.2554 ([M − H]^−^, calcd for C_37_H_41_O_13_, 693.2553).

*Urdamycin N5* (**5**). Dark green powder; [α${]}_{D}^{20}$ + 250 (*c* 0.01, CDCl_3_); UV (CDCl_3_) *λ*_max_ (log *ε*) 240 (4.98), 322 (4.85), 437 (4.46), IR *ν_max_* 3392, 2924, 1626, 1435, 1267, 1061, 1013, 773 cm^−1^; ^1^H NMR (700 MHz, DMSO-*d_6_*/CD_3_OD) and ^13^C NMR (176 MHz, DMSO-*d_6_*/CD_3_OD) data, Table 2; (-)HR-ESI-MS *m*/*z* 677.2606 ([M − H]^−^, calcd for C_37_H_41_O_12_, 677.2604).

*Urdamycin N6* (**6**). Red powder; [α${]}_{D}^{20}$ + 325 (*c* 0.06, CDCl_3_); UV (CDCl_3_) *λ*_max_ (log *ε*) 219 (4.57), 257 (4.19), 291 (4.04), 325 (3.67), 454 (3.90), IR *ν_max_* 3379, 2928, 1614, 1435, 1246, 1086, 752 cm^−1^; ^1^H NMR (700 MHz, CDCl_3_) and ^13^C NMR (176 MHz, CDCl_3_) data, Table 2; (-)HR-ESI-MS *m*/*z* 495.1667 ([M − H]^−^, calcd for C_27_H_27_O_9_, 495.1661).

*Urdamycin N7* (**7**). Red powder; [α${]}_{D}^{20}$ + 130 (*c* 0.06, CDCl_3_); UV (CDCl_3_) *λ*_max_ (log *ε*) 218 (4.50), 259 (4.13), 284 (4.03), 325 (3.64), 451 (3.85), IR *ν_max_* 3360, 2918, 1612, 1435, 1238, 1086, 1060, 756 cm^−1^; ^1^H NMR (700 MHz, CDCl_3_) and ^13^C NMR (176 MHz, CDCl_3_) data, Table 3; (-)HR-ESI-MS *m*/*z* 495.1675 ([M − H]^−^, calcd for C_27_H_27_O_9_, 495.1661).

*Urdamycin N8* (**8**). Red powder; [α${]}_{D}^{20}$ + 75 (*c* 0.04, CDCl_3_); UV (CDCl_3_) *λ*_max_ (log *ε*) 240 (4.39), 266 (4.27), 465 (3.91), IR *ν_max_* 3379, 2932, 1612, 1435, 1238, 1057, 1009, 748 cm^−1^; ^1^H NMR (700 MHz, CDCl_3_) and ^13^C NMR (176 MHz, CDCl_3_) data, Table 3; (-)HR-ESI-MS *m*/*z* 739.2984 ([M − H]^−^, calcd for C_39_H_47_O_14_, 739.2971).

*Urdamycin N9* (**9**). Red powder; [α${]}_{D}^{20}$ + 233 (*c* 0.04, CDCl_3_); UV (CDCl_3_) *λ*_max_ (log *ε*) 240 (4.31), 265 (4.28), 296 (4.08), 472 (3.94), IR *ν_max_* 3395, 2932, 1614, 1435, 1369, 1248, 1088, 1059, 1010.7, 756 cm^−1^; ^1^H NMR (700 MHz, CDCl_3_) and ^13^C NMR (176 MHz, CDCl_3_) data, Table 3; (-)HR-ESI-MS *m*/*z* 739.2974 ([M − H]^−^, calcd for C_39_H_47_O_14_, 739.2971).

### 3.5. Electronic Circular Dichroism (ECD) Calculation

Monte Carlo conformational searches were carried out by means of the Spartan’s 10 software (Wavefunction, Inc., Irvine, CA, USA) using Merck Molecular Force Field (MMFF). The conformers with Boltzmann-population of over 5% were chosen for electronic circular dichroism (ECD) calculations, and then the conformers were initially optimized at B3LYP/6-31+g (d, p) level in methanol using the conductor-like polarizable continuum model (CPCM). The theoretical calculation of ECD was conducted in methanol using Time-dependent Density functional theory (TD-DFT) at the B3LYP/6-311+g (d, p) level for all conformers of compounds **6** and **7**. Rotatory strengths for a total of 10 excited states were calculated. ECD spectra were generated using the program SpecDis 1.6 (University of Würzburg, Würzburg, Germany) and GraphPad Prism 5 (University of California San Diego, San Diego, CA, USA) from dipole-length rotational strengths by applying Gaussian band shapes with sigma = 0.3 eV.

## 4. Conclusions

The early report of urdamycin derivatives, urdamycins A–F, were isolated from *Streptomyces fradiae* Tu 2717 by Drautz in 1986. Since then, many urdamycin congeners were discovered. Structurally, urdamycins have different aglycone parts, whereas the sugar moieties are always the same [10]. Most of them were decorated with a trisaccharide chain composed of *β*-olivose-(1→4)-*α*-rhodinose-(1→3)-*β*-olivose via a C-C linkage. Diverse biological activities of these angucycline antibiotics were evaluated, and the most important were their cytotoxicities against the tumor cell lines. At the same time, this potent cytotoxicity also limited their use in the clinic. In this study, nine new angucycline glycosides, urdamycin N1–N9 (**1**–**9**), together with two known congener urdamycins A (**10**) and B (**11**) were obtained from a mangrove-derived *Streptomyces diastaticus* subsp. SCSIO GJ056. Compounds **7** and **8** are the first naturally occurring (5*R*, 6*R*) angucycline glycosides. We will further investigate the biological activity of these new angucycline compounds.

## Figures and Tables

**Table 1:** The ^1^H and ^13^C NMR data of compounds **1**–**3** (*δ* in ppm, *J* in Hz).

Position	1 ^a^		2 ^b^		3 ^c^	
	*δ*_C_	*δ*_H_	*δ*_C_	*δ*_H_	*δ*_C_	*δ*_H_
1	198.4		155.3		155.2	
2	53.2	2.90, dd (14, 1.1); 3.0, m	118.7	6.99, s	118.8	6.98, s
3	71.8		140.8		140.9	
4	37.5	3.18, m; 2.80, d (18.1)	113.0	7.58, s	113.2	7.58, m
4a	137.9		126.0		126.0	
5	160.9		160.3		160.5	
6	108.2	7.65, s	99.8	7.54, s	100.0	7.54, s
6a	135.1		135.8		136.1	
7	187.9		187.5		187.6	
7a	114.8		113.9		114.1	
8	158.0		156.8		157.0	
9	136.6		136.8		137.3	
10	133.9	7.81, d (7.8)	133.4	7.82, d (7.6)	133.3	7.80, m
11	119.5	7.59, d (7.8)	119.9	7.68, d (7.6)	120.0	7.67, m
11a	133.8		133.7		133.8	
12	182.3		185.6		186.0	
12a	128.4		129.8		129.9	
12b	137.4		120.4		120.5	
13	30.1	1.42, s	21.2	2.42, s	21.2	2.41, s
14	56.5	3.99, s	56.6	4.14, s	56.6	4.13, s
1′	71.1	4.82, d (10.6)	70.4	4.8, d (13.7)	70.8	4.78, d (10.9)
2′	37.6	1.41, m	39.7	1.35, m	40.0	1.31, dd (11.4)
		2.42, dd (12.3, 4.2)		2.01, m		2.28, d (10.2)
3′	81.6	3.67, ddd (11.4, 8.4, 5.1)	74.6	3.69, ddd (11.1, 8.9, 4.9)	71.7	3.55, t (11.8)
4′	76.0	3.13, m	74.5	3.06, td (8.9, 5.4)	77.1	2.90, t (8.8)
5′	76.1	3.45, m	76.2	3.47, q (6.0)	76.3	3.38, m
6′	18.4	1.37, d (6.1)	18.5	1.30, d (6.1)	18.5	1.28, d (6.1)
1″	97.4	4.96, s	91.9	4.90, d (2.3)		
2″	25.1	1.47, m	24.1	1.29, m		
		2.06, m		1.83, m		
3″	24.5	1.9, m	24.1	1.76, m; 1.95, m		
4″	76.2	3.49, m	75.4	3.44, m		
5″	67.5	4.09, m	65.3	4.15, m		
6″	16.9	1.16, d (6.5)	17.0	1.05, d (6.5)		
1″′	101.5	4.45, dd (9.7, 1.5)	101.0	4.48, dd (9.7, 1.6)		
2″′	38.9	2.19, ddd (12.5, 4.8, 1.5)	36.0	1.24, m		
		1.60, td (12.1, 10.0)		2.47, m		
3″′	71.4	3.45, m	70.3	3.33, m		
4″′	77.0	2.95, t (8.9)	76.8	2.72, m		
5″′	71.7	3.16, m	71.6	3.11, dq (9.0, 6.2)		
6″′	17.7	1.23, d (6.2)	18.2	1.14, d (6.2)		

^a^ Recorded in CDCl_3_-CD_3_OD (9:1); ^b^ Recorded in DMSO; ^c^ Recorded in DMSO-CD_3_OD (9:1).

**Table 2:** The ^1^H and ^13^C NMR data of compounds **4**–**6** (*δ* in ppm, *J* in Hz).

Position	4 ^a^		5 ^a^		6 ^b^	
	*δ*_C_	*δ*_H_	*δ*_C_	*δ*_H_	*δ*_C_	*δ*_H_
1	155.4		155.0		157.1	
2	119.6	6.97, m	117.3	7.0, s	126.0	6.73, s
3	139.7		141.5		144.0	
4	114.4	7.63, m	119.5	7.37, s	123.0	6.89, s
4a	121.7		138.6		139.2	
5	163.6		135.4	8.22, d (8.5)	78.1	4.23, d (2.8)
6	105.6	7.54, s	121.5	8.18, d (8.5)	70.2	4.91, d (2.8)
6a	136.5		133.8		141.6	
7	188.7		188.0		189.1	
7a	114.2		114.4		113.7	
8	156.8		156.7		157.6	
9	137.5		136.8		135.9	
10	133.6	7.8, m	133.5	7.86, d (7.8)	133.0	7.88, d (7.9)
11	120.2	7.71, m	118.7	7.67, d (7.8)	121.4	7.80, d (7.9)
11a	134.3		134.5		131.5	
12	184.6		186.8		189.0	
12a	130.4		134.4		143.0	
12b	122.1		119.9		112.9	
13	21.1	2.40, s	21.1	2.45, s	21.4	2.34, s
14					58.5	3.45, s
15					56.7	3.26, s
1′	70.6	4.78, dd (11.1, 3.0)	70.5	4.83, d (11.4)	71.4	4.92, d (11.0)
2′	40.0	1.36, dd (22.4, 11.2)	39.5	1.35, td (11.9, 9.9)	39.5	1.46, dd (23.8, 11.7)
		2.02, dd (12.2, 5.0)		2.01, ddd (12.2, 5.0, 1.5)		2.54, dd (12.1, 3.9 )
3′	74.9	3.69, ddd (11.2, 9.0, 4.9)	74.9	3.70, ddd (11.1, 8.9, 4.8)	73.2	3.85, m
4′	74.8	3.05, t (9.0)	74.7	3.06, t (8.9)	78.2	3.21, t (8.9)
5′	76.4	3.48, m	76.3	3.48, overlapped	76.0	3.53, dt (15.1, 6.1 )
6′	18.6	1.29, d (6.0)	18.5	1.30, d (6.1)	18.2	1.42, d (6.1)
1″	92.4	4.89, m	92.2	4.89, d (2.7)		
2″	24.3	1.27, m	24.2	1.26, m		
		1.84, m		1.83, m		
3″	24.3	1.77, m; 1.96, m	24.2	1.77, m; 1.95, m		
4″	75.7	3.44, m	75.6	3.44, overlapped		
5″	65.6	4.15, q (6.5)	65.5	4.15, q ( 6.4)		
6″	17.1	1.05, d (6.5)	17.0	1.04, d (6.4)		
1″′	101.3	4.47, d (9.6)	101.2	4.47, d (9.7)		
2″′	36.3	1.23, m; 2.46, m	36.2	1.24, m; 2.47, m		
3″′	70.6	3.32, m	70.5	3.33, ddd (11.8, 8.7, 5.1)		
4″′	77.0	2.72, t (8.8)	76.9	2.72, t (8.7)		
5″′	71.8	3.1, m	71.7	3.1, m		
6″′	18.3	1.14, d (6.2)	18.2	1.14, d (6.1)		

^a^ Recorded in DMSO-CD_3_OD (9:1); ^b^ Recorded in CDCl_3_.

**Table 3:** ^1^H and ^13^C-NMR data of compounds **7**–**9** in CDCl_3_ (*δ* in ppm, *J* in Hz).

Position	7		8		9	
	*δ*_C_	*δ*_H_	*δ*_C_	*δ*_H_	*δ*_C_	*δ*_H_
1	156.5		156.5		157.1	
2	120.6	6.81, s	120.6	6.81, s	126.0	6.73, s
3	145.4		145.4		144.0	
4	119.0	7.17, s	119.0	7.16, s	123.0	6.89, s
4a	138.8		138.8		139.5	
5	80.0	4.27, d (2.2)	80.0	4.27, d (2.5)	78.1	4.23, d (2.9)
6	67.4	4.99, d (3.1)	67.4	4.99, d (3.1)	70.2	4.91, d (2.9)
6a	140.2		140.2		141.6	
7	189.0		188.9		189.1	
7a	113.6		113.5		113.7	
8	157.6		157.7		157.7	
9	139.4		139.7		135.9	
10	133.2	7.90, d (7.9)	133.2	7.89, d (7.9)	133.0	7.87, d (7.8)
11	121.5	7.80, d (7.9)	121.5	7.78, d (7.9)	121.4	7.79, d (7.8)
11a	131.3		131.3		131.4	
12	188.7		188.8		189.0	
12a	143.1		143.2		143.0	
12b	112.5		112.5		112.9	
13	21.9	2.36, s	21.9	2.36, s	21.4	2.34, s
14	58.2	3.69, s	58.2	3.69, s	58.5	3.44, s
15	59.3	3.42, s	59.3	3.41, s	56.7	3.26, s
1′	71.4	4.94, d (11.0)	71.3	4.89, dd (9.5, 1.1)	71.4	4.87, d (10.4)
2′	39.4	1.46, d (12.1)	37.7	1.48, d (11.7)	37.7	1.47, m
		2.53, dd (12.7, 3.5)		2.48, m		2.50, ddd (13.0, 4.9, 1.7 )
3′	73.2	3.87, m	82.4	3.72, ddd (11.6, 8.3, 5.1 )	82.5	3.71, ddd (11.5, 8.3, 5.1)
4′	78.2	3.22, t (8.9)	76.3	3.18, t (8.7)	76.3	3.18, t (8.7)
5′	76.1	3.54, dt (15.1, 6.0)	76.3	3.51, td (12.2, 6.1)	76.3	3.51, q (6.0)
6′	18.2	1.43, d (6.1)	18.6	1.44, d (6.0)	18.6	1.44, d (6.0)
1″			97.9	5.04, s	98.0	5.03, s
2″			25.3	1.55, d (13.9)	25.4	1.54, d (13.8)
				2.15, m		2.13, m
3″			24.7	1.95, m	24.7	1.96, m
				2.13, m		2.13, m
4″			76.1	3.55, m	76.1	3.55, m
5″			67.7	4.13, dd (12.9, 6.4 )	67.7	4.13, q (6.0)
6″			17.2	1.24, d (6.5)	17.1	1.23, d (6.5)
1′′′			101.5	4.55, dd (9.5, 1.1)	101.5	4.52, dd (9.6, 1.6)
2′′′			39.3	1.71, dd (21.9, 12.1)	39.3	1.71, td (12.1, 9.9)
				2.30, dd (12.5, 5.0 )		2.29, ddd (12.4, 4.9, 1.5)
3′′′			71.7	3.59, m	71.7	3.59, m
4′′′			77.7	3.11, t (8.9)	77.7	3.11, t (8.9)
5′′′			72.0	3.26, dq ( 9.1, 6.1)	72.0	3.24, m
6′′′			17.9	1.31, d (6.1)	17.9	1.31, d (6.2)

## Supplementary Materials

The following are available online at http://www.mdpi.com/1660-3397/16/6/185/s1. This section includes 1D, 2D NMR spectra for new compounds **1**–**9**, and computational details of **6** and **7**.
