# Supplementary material for: Angucycline Glycosides from Mangrove-Derived Streptomyces diastaticus subsp. SCSIO GJ056
Source: Mar Drugs. 2018 May 28;16(6):185. doi: 10.3390/md16060185 (PMC6025406; doi:10.3390/md16060185)

Supporting Information

Angucycline Glycosides from Mangrove-Derived *Streptomyces diastaticus* subsp. SCSIO GJ056

Chun Gui [1,2], Yena Liu [3], Zhenbin Zhou [1,2], Shanwen Zhang [1,2], Yunfeng Hu [1], Yu-Cheng Gu [4], Hongbo Huang [1,*] and Jianhua Ju [1,2,*]

[1] CAS Key Laboratory of Tropical Marine Bio-resources and Ecology, Guangdong Key Laboratory of Marine Materia Medica, RNAM Center for Marine Microbiology, South China Sea Institute of Oceanology, Chinese Academy of Sciences, 164 West Xingang Road, Guangzhou 510301, China; guichun1988@sina.com (C.G.); zzb1881396@163.com (Z.Z.); sherry920111@163.com (S.Z.); yunfeng.hu@scsio.ac.cn (Y.H.)

[2] University of Chinese Academy of Sciences, 19 Yuquan Road, Beijing 110039, China

[3] State Key Laboratory of Oncology in South China, Collaborative Innovation Center for Cancer Medicine, Sun Yat-Sen University Cancer Center, Guangzhou 510060, China; LIUYN@sysucc.org.cn

[4] Syngenta Jealott's Hill International Research Centre, Bracknell, Berkshire RG42 6EY, U.K.; yucheng.gu@syngenta.com

* Correspondence: huanghb@scsio.ac.cn (H.H.); jju@scsio.ac.cn (J.J.); Tel./Fax: +86-20-3406-6449 (H.H.); Tel./Fax: +86-20-8902-3028 (J.J.)

Table of Contents

		Page
**Table S1**.	Gibbs free energies[a] and equilibrium populations[b] of low-energy conformers of 5*S*, 6*S*-**6**.	S2
**Table S2**.	Cartesian coordinates for the low-energy reoptimized MMFF conformers of 5*S*, 6*S*-**6** at B3LYP/6-311+G (d, p) level of theory in methanol.	S2
**Table S3**.	Gibbs free energies[a] and equilibrium populations[b] of low-energy conformers of 5*R*, 6*R*-**7**.	S10
**Table S4**.	Cartesian coordinates for the low-energy reoptimized MMFF conformers of 5*R*, 6*R*-**7** at B3LYP/6-311+G (d, p) level of theory in methanol.	S11
**Figure S1-S7**.	1D and 2D NMR spectrum of compound **1**.	S20-26
**Figure S8-S14**.	1D and 2D NMR spectrum of compound **2**.	S27-33
**Figure S15-S21**.	1D and 2D NMR spectrum of compound **3**.	S34-40
**Figure S22-S28**.	1D and 2D NMR spectrum of compound **4**.	S41-47
**Figure S29-S35**.	1D and 2D NMR spectrum of compound **5**.	S48-54
**Figure S36-S42**.	1D and 2D NMR spectrum of compound **6**.	S55-61
**Figure S43-S49**.	1D and 2D NMR spectrum of compound **7**.	S62-68
**Figure S50-S56**.	1D and 2D NMR spectrum of compound **8**.	S69-75
**Figure S57-S63**.	1D and 2D NMR spectrum of compound **9**.	S76-82

Table S1. Gibbs free energies[a] and equilibrium populations[b] of low-energy conformers of 5*S*, 6*S*-**6**.

Conformers	In methanol	
	Δ*G*	*P* (%)
**6a**	0.19	21.41
**6b**	0	33.99
**6c**	1.26	9.02
**6d**	1.26	9.02
**6e**	0.17	26.56

[a]B3LYP/6-31+G(d,p), in kcal/mol. [b]From Δ*G* values at 298.15K.

Table S2. Cartesian coordinates for the low-energy reoptimized MMFF conformers of 5*S*, 6*S*-**6** at B3LYP/6-311+G (d, p) level of theory in methanol.

**6a**		Standard Orientation (Ångstroms)			
Center number	Atomic number	Atomic Type	X	Y	Z
1.	6.	0.	-2.489164	-1.692739	0.132580
2.	6.	0.	-2.876234	-0.361024	0.234076
3.	6.	0.	-1.876124	0.641492	0.218574
4.	6.	0.	-0.515890	0.280471	0.111762
5.	6.	0.	-0.156760	-1.076871	0.003965
6.	6.	0.	-1.142438	-2.058928	0.012506
7.	6.	0.	0.530855	1.297727	0.116096
8.	6.	0.	1.940794	0.886720	0.019297
9.	6.	0.	2.337380	-0.417624	-0.160880
10.	6.	0.	1.266138	-1.472384	-0.132134
11.	6.	0.	2.971484	1.994657	0.159700
12.	6.	0.	4.145420	1.454256	0.966901
13.	6.	0.	4.697863	0.220798	0.283343
14.	6.	0.	3.785759	-0.741381	-0.250233
15.	8.	0.	0.241656	2.513150	0.198068
16.	8.	0.	1.531194	-2.679755	-0.185030
17.	8.	0.	3.632901	1.197290	2.272552
18.	8.	0.	-2.264713	1.923895	0.315290
19.	6.	0.	6.067575	0.077473	0.174339
20.	6.	0.	6.637351	-1.023209	-0.494815
21.	6.	0.	5.775200	-1.961989	-1.029387
22.	6.	0.	4.373552	-1.856642	-0.916510
23.	8.	0.	3.705231	-2.851187	-1.527338
24.	6.	0.	-7.124481	-0.296717	-0.266691
25.	6.	0.	-6.379021	0.933454	-0.777723
26.	6.	0.	-4.891149	0.626007	-0.957172
27.	6.	0.	-4.328056	0.044119	0.358214

28.	8.	0.	-5.077768	-1.099520	0.752090
29.	6.	0.	-6.458545	-0.825030	1.011569
30.	8.	0.	-8.477098	0.017516	0.022956
31.	6.	0.	-7.091811	-2.108590	1.523239
32.	8.	0.	-7.029144	1.306699	-1.996550
33.	6.	0.	4.622973	0.896339	3.238085
34.	8.	0.	3.503245	2.422493	-1.089159
35.	6.	0.	2.648593	3.259507	-1.850331
36.	6.	0.	8.133925	-1.161369	-0.617603
37.	1.	0.	6.713851	0.845359	0.593562
38.	1.	0.	-3.260408	-2.453792	0.161329
39.	1.	0.	-0.853285	-3.100147	-0.069352
40.	1.	0.	2.518479	2.836356	0.694159
41.	1.	0.	4.927060	2.226721	1.013108
42.	1.	0.	-1.429290	2.469612	0.302340
43.	1.	0.	6.151182	-2.834104	-1.555688
44.	1.	0.	2.819635	-2.940773	-1.098527
45.	1.	0.	-7.059764	-1.080490	-1.040525
46.	1.	0.	-6.504558	1.732787	-0.028792
47.	1.	0.	-4.334725	1.529535	-1.230909
48.	1.	0.	-4.766586	-0.109737	-1.760365
49.	1.	0.	-4.405692	0.818826	1.139278
50.	1.	0.	-6.532613	-0.040675	1.783765
51.	1.	0.	-8.801049	0.504479	-0.753621
52.	1.	0.	-6.594592	-2.438161	2.440664
53.	1.	0.	-8.152634	-1.943474	1.728807
54.	1.	0.	-6.999499	-2.902818	0.774041
55.	1.	0.	-6.710138	2.185116	-2.254672
56.	1.	0.	4.100863	0.767209	4.189549
57.	1.	0.	5.168971	-0.026390	2.999327
58.	1.	0.	5.350313	1.718063	3.337947
59.	1.	0.	3.231331	3.581765	-2.717267
60.	1.	0.	1.751253	2.732120	-2.201274
61.	1.	0.	2.329141	4.140682	-1.276428
62.	1.	0.	8.570530	-0.289041	-1.119780
63.	1.	0.	8.607617	-1.231701	0.369771
64.	1.	0.	8.409433	-2.053113	-1.188091

6b		Standard Orientation (Ångstroms)			
Center number	Atomic number	Atomic Type	X	Y	Z
1.	6.	0.	-2.543703	-1.684707	0.292404
2.	6.	0.	-2.914804	-0.344518	0.300667
3.	6.	0.	-1.900163	0.642871	0.266055
4.	6.	0.	-0.543436	0.258947	0.221859
5.	6.	0.	-0.199953	-1.106748	0.206905
6.	6.	0.	-1.198852	-2.074349	0.245036
7.	6.	0.	0.518253	1.259930	0.207018
8.	6.	0.	1.922331	0.819760	0.144358
9.	6.	0.	2.301266	-0.491728	-0.005502
10.	6.	0.	1.221101	-1.526335	0.145658
11.	6.	0.	2.937757	1.935422	0.193604
12.	6.	0.	4.234322	1.429634	0.819019
13.	6.	0.	4.697118	0.168544	0.123792
14.	6.	0.	3.734004	-0.832831	-0.207728
15.	8.	0.	0.252114	2.481603	0.241793
16.	8.	0.	1.480673	-2.730636	0.259663
17.	8.	0.	3.927317	1.226634	2.198679
18.	8.	0.	-2.271433	1.934125	0.283302
19.	6.	0.	6.040529	0.025994	-0.169915
20.	6.	0.	6.529232	-1.120310	-0.824816
21.	6.	0.	5.617632	-2.108961	-1.145602
22.	6.	0.	4.244463	-2.002101	-0.845510
23.	8.	0.	3.517101	-3.055287	-1.260660
24.	6.	0.	-7.135323	-0.254413	-0.389793
25.	6.	0.	-6.349942	0.936133	-0.933433
26.	6.	0.	-4.860379	0.600988	-1.028014
27.	6.	0.	-4.364239	0.085616	0.341024
28.	8.	0.	-5.147943	-1.024643	0.763169
29.	6.	0.	-6.534615	-0.719142	0.944382
30.	8.	0.	-8.494795	0.092011	-0.179142
31.	6.	0.	-7.208932	-1.964374	1.496641
32.	8.	0.	-6.939567	1.248564	-2.199202
33.	6.	0.	5.047151	0.945659	3.017544
34.	8.	0.	3.151575	2.407034	-1.134381
35.	6.	0.	3.239825	3.819396	-1.232550
36.	6.	0.	7.994670	-1.256548	-1.153975
37.	1.	0.	6.727593	0.824902	0.100627
38.	1.	0.	-3.326518	-2.433094	0.336143
39.	1.	0.	-0.922256	-3.122217	0.238912
40.	1.	0.	2.537876	2.733839	0.826293

41.	1.	0.	5.010951	2.204414	0.721060
42.	1.	0.	-1.427977	2.466912	0.275359
43.	1.	0.	5.931794	-3.022283	-1.641718
44.	1.	0.	2.699487	-3.109001	-0.709430
45.	1.	0.	-7.048231	-1.080537	-1.115920
46.	1.	0.	-6.497173	1.777409	-0.236141
47.	1.	0.	-4.279006	1.480904	-1.325684
48.	1.	0.	-4.710757	-0.180129	-1.782555
49.	1.	0.	-4.463115	0.903019	1.074742
50.	1.	0.	-6.631428	0.107721	1.668270
51.	1.	0.	-8.776811	0.538693	-0.995275
52.	1.	0.	-6.758766	-2.247906	2.452942
53.	1.	0.	-8.275502	-1.775408	1.643527
54.	1.	0.	-7.094010	-2.800421	0.797776
55.	1.	0.	-6.593919	2.105639	-2.492444
56.	1.	0.	4.671575	0.865801	4.040886
57.	1.	0.	5.539214	0.003759	2.740464
58.	1.	0.	5.791927	1.756716	2.972447
59.	1.	0.	3.418019	4.047663	-2.286647
60.	1.	0.	2.305500	4.298422	-0.909527
61.	1.	0.	4.073255	4.224856	-0.638510
62.	1.	0.	8.323798	-0.453534	-1.825538
63.	1.	0.	8.612523	-1.190203	-0.249728
64.	1.	0.	8.209724	-2.212166	-1.640870

**6c**		Standard Orientation (Ångstroms)			
Center number	Atomic number	Atomic Type	X	Y	Z
1.	6.	0.	-2.467909	-1.708636	0.267512
2.	6.	0.	-2.847310	-0.370838	0.262877
3.	6.	0.	-1.841259	0.621393	0.169951
4.	6.	0.	-0.483251	0.245230	0.089595
5.	6.	0.	-0.131904	-1.118334	0.089847
6.	6.	0.	-1.123311	-2.090747	0.177397
7.	6.	0.	0.569483	1.252831	0.009369
8.	6.	0.	1.975337	0.827997	-0.065217
9.	6.	0.	2.368262	-0.489513	-0.123798
10.	6.	0.	1.288575	-1.532729	-0.015460

11.	6.	0.	3.009993	1.937538	-0.039286
12.	6.	0.	4.184035	1.481513	0.828988
13.	6.	0.	4.724558	0.177081	0.291774
14.	6.	0.	3.814504	-0.824516	-0.167128
15.	8.	0.	0.286252	2.473714	0.003410
16.	8.	0.	1.541952	-2.742712	0.018981
17.	8.	0.	3.784014	1.280920	2.183419
18.	8.	0.	-2.222091	1.910009	0.166790
19.	6.	0.	6.093882	0.006803	0.248194
20.	6.	0.	6.668167	-1.162473	-0.284930
21.	6.	0.	5.810446	-2.138232	-0.757773
22.	6.	0.	4.408503	-2.007263	-0.702523
23.	8.	0.	3.747356	-3.049224	-1.236893
24.	6.	0.	-7.091661	-0.313254	-0.270193
25.	6.	0.	-6.333661	0.872674	-0.860011
26.	6.	0.	-4.847876	0.541381	-1.008003
27.	6.	0.	-4.297112	0.051671	0.349373
28.	8.	0.	-5.057206	-1.055864	0.819300
29.	6.	0.	-6.438678	-0.756758	1.046955
30.	8.	0.	-8.443758	0.029826	-0.012631
31.	6.	0.	-7.083687	-1.998917	1.639491
32.	8.	0.	-6.973421	1.164896	-2.106138
33.	6.	0.	3.678080	2.469607	2.946520
34.	8.	0.	3.555915	2.226065	-1.322948
35.	6.	0.	2.701384	2.956045	-2.187020
36.	6.	0.	8.165425	-1.332944	-0.334294
37.	1.	0.	6.736624	0.801395	0.619070
38.	1.	0.	-3.243331	-2.460757	0.355796
39.	1.	0.	-0.840236	-3.136815	0.179498
40.	1.	0.	2.547687	2.839206	0.376553
41.	1.	0.	4.968794	2.249326	0.775771
42.	1.	0.	-1.382849	2.447279	0.110272
43.	1.	0.	6.190830	-3.061819	-1.183410
44.	1.	0.	2.847091	-3.089275	-0.832101
45.	1.	0.	-7.027867	-1.149168	-0.987514
46.	1.	0.	-6.457290	1.723078	-0.169312
47.	1.	0.	-4.282199	1.417973	-1.343059
48.	1.	0.	-4.725369	-0.252276	-1.754323
49.	1.	0.	-4.373570	0.881012	1.072423
50.	1.	0.	-6.514793	0.078776	1.763325
51.	1.	0.	-8.759114	0.463352	-0.823646
52.	1.	0.	-6.989059	-2.843163	0.947505
53.	1.	0.	-6.596548	-2.267188	2.581973
54.	1.	0.	-8.145301	-1.814659	1.823526

55.	1.	0.	-6.645709	2.020548	-2.423143
56.	1.	0.	3.474039	2.161483	3.975251
57.	1.	0.	4.616269	3.045954	2.925781
58.	1.	0.	2.860738	3.123409	2.608351
59.	1.	0.	3.290465	3.183461	-3.079373
60.	1.	0.	1.815816	2.377689	-2.484447
61.	1.	0.	2.362829	3.894118	-1.724465
62.	1.	0.	8.637881	-0.514870	-0.892163
63.	1.	0.	8.597018	-1.323005	0.674571
64.	1.	0.	8.446962	-2.275754	-0.812260

**6d**		Standard Orientation (Ångstroms)			
Center number	Atomic number	Atomic Type	X	Y	Z
1.	6.	0.	-2.467904	-1.708579	0.267506
2.	6.	0.	-2.847302	-0.370779	0.262818
3.	6.	0.	-1.841247	0.621453	0.169923
4.	6.	0.	-0.483224	0.245283	0.089651
5.	6.	0.	-0.131891	-1.118280	0.089980
6.	6.	0.	-1.123306	-2.090692	0.177486
7.	6.	0.	0.569517	1.252887	0.009494
8.	6.	0.	1.975375	0.828024	-0.065144
9.	6.	0.	2.368281	-0.489486	-0.123650
10.	6.	0.	1.288597	-1.532693	-0.015137
11.	6.	0.	3.010053	1.937547	-0.039274
12.	6.	0.	4.184119	1.481537	0.828995
13.	6.	0.	4.724602	0.177087	0.291774
14.	6.	0.	3.814515	-0.824505	-0.167050
15.	8.	0.	0.286319	2.473771	0.003613
16.	8.	0.	1.542002	-2.742656	0.019655
17.	8.	0.	3.784202	1.280972	2.183432
18.	8.	0.	-2.222068	1.910061	0.166741
19.	6.	0.	6.093927	0.006796	0.248071
20.	6.	0.	6.668148	-1.162464	-0.285139
21.	6.	0.	5.810376	-2.138211	-0.757935
22.	6.	0.	4.408449	-2.007244	-0.702521
23.	8.	0.	3.747216	-3.049177	-1.236854
24.	6.	0.	-7.091661	-0.313305	-0.270242

25.	6.	0.	-6.333687	0.872585	-0.860157
26.	6.	0.	-4.847907	0.541282	-1.008158
27.	6.	0.	-4.297109	0.051731	0.349261
28.	8.	0.	-5.057182	-1.055774	0.819324
29.	6.	0.	-6.438660	-0.756680	1.046942
30.	8.	0.	-8.443769	0.029765	-0.012684
31.	6.	0.	-7.083635	-1.998808	1.639585
32.	8.	0.	-6.973494	1.164727	-2.106283
33.	6.	0.	3.677894	2.469691	2.946436
34.	8.	0.	3.555991	2.226053	-1.322937
35.	6.	0.	2.701229	2.955557	-2.187180
36.	6.	0.	8.165397	-1.332985	-0.334611
37.	1.	0.	6.736708	0.801373	0.618911
38.	1.	0.	-3.243347	-2.460683	0.355765
39.	1.	0.	-0.840211	-3.136755	0.179659
40.	1.	0.	2.547757	2.839236	0.376526
41.	1.	0.	4.968877	2.249355	0.775701
42.	1.	0.	-1.382807	2.447308	0.110314
43.	1.	0.	6.190720	-3.061780	-1.183647
44.	1.	0.	2.847081	-3.089280	-0.831825
45.	1.	0.	-7.027867	-1.149275	-0.987494
46.	1.	0.	-6.457297	1.723042	-0.169520
47.	1.	0.	-4.282242	1.417838	-1.343326
48.	1.	0.	-4.725412	-0.252463	-1.754386
49.	1.	0.	-4.373579	0.881139	1.072231
50.	1.	0.	-6.514808	0.078915	1.763238
51.	1.	0.	-8.759125	0.463321	-0.823684
52.	1.	0.	-8.145233	-1.814532	1.823697
53.	1.	0.	-6.989071	-2.843089	0.947635
54.	1.	0.	-6.596417	-2.267023	2.582041
55.	1.	0.	-6.645559	2.020216	-2.423501
56.	1.	0.	3.474503	2.161567	3.975295
57.	1.	0.	4.615724	3.046602	2.925200
58.	1.	0.	2.860004	3.122952	2.608546
59.	1.	0.	3.290193	3.182843	-3.079642
60.	1.	0.	1.815803	2.376864	-2.484389
61.	1.	0.	2.362458	3.893685	-1.724893
62.	1.	0.	8.596973	-1.323705	0.674268
63.	1.	0.	8.446876	-2.275510	-0.813171
64.	1.	0.	8.637915	-0.514587	-0.891954

**6e**		Standard Orientation (Ångstroms)			
Center number	Atomic number	Atomic Type	X	Y	Z
1.	6.	0.	-2.493451	-1.680644	0.146493
2.	6.	0.	-2.877764	-0.347987	0.243227
3.	6.	0.	-1.874836	0.652187	0.230054
4.	6.	0.	-0.515270	0.287043	0.126141
5.	6.	0.	-0.159279	-1.071424	0.023123
6.	6.	0.	-1.147289	-2.051030	0.032704
7.	6.	0.	0.533940	1.301696	0.126301
8.	6.	0.	1.942941	0.886825	0.023949
9.	6.	0.	2.336031	-0.419244	-0.149871
10.	6.	0.	1.262946	-1.471275	-0.107728
11.	6.	0.	2.976098	1.994219	0.148955
12.	6.	0.	4.155856	1.458471	0.950524
13.	6.	0.	4.700966	0.218427	0.273041
14.	6.	0.	3.783481	-0.746490	-0.245846
15.	8.	0.	0.248670	2.517633	0.207659
16.	8.	0.	1.525896	-2.679820	-0.145589
17.	8.	0.	3.654363	1.212761	2.262792
18.	8.	0.	-2.261083	1.934035	0.326241
19.	6.	0.	6.069721	0.071743	0.155389
20.	6.	0.	6.632738	-1.035593	-0.508191
21.	6.	0.	5.765088	-1.977679	-1.028022
22.	6.	0.	4.364527	-1.868641	-0.906121
23.	8.	0.	3.689835	-2.867675	-1.502991
24.	6.	0.	-7.112078	-0.293353	-0.283222
25.	6.	0.	-6.371327	0.929103	-0.821677
26.	6.	0.	-4.884613	0.619080	-0.971317
27.	6.	0.	-4.328759	0.063492	0.355458
28.	8.	0.	-5.080612	-1.074817	0.776847
29.	6.	0.	-6.459413	-0.788134	1.019605
30.	8.	0.	-8.470445	0.115454	-0.090178
31.	6.	0.	-7.091526	-2.057313	1.572100
32.	8.	0.	-6.891679	1.324315	-2.080288
33.	6.	0.	4.653016	0.922648	3.222613
34.	8.	0.	3.498314	2.412160	-1.107213
35.	6.	0.	2.638062	3.243990	-1.868344
36.	6.	0.	8.128162	-1.177115	-0.641314
37.	1.	0.	6.720229	0.842222	0.563148
38.	1.	0.	-3.266153	-2.440369	0.172928
39.	1.	0.	-0.860448	-3.093189	-0.045180
40.	1.	0.	2.528220	2.840272	0.680801

41.	1.	0.	4.938868	2.230189	0.983846
42.	1.	0.	-1.426769	2.480362	0.309756
43.	1.	0.	6.135789	-2.855103	-1.549237
44.	1.	0.	2.807801	-2.952033	-1.066050
45.	1.	0.	-7.051175	-1.092543	-1.037862
46.	1.	0.	-6.500472	1.741625	-0.084307
47.	1.	0.	-4.339393	1.522079	-1.260843
48.	1.	0.	-4.749394	-0.128405	-1.762196
49.	1.	0.	-4.407458	0.851099	1.122478
50.	1.	0.	-6.539323	0.018896	1.767719
51.	1.	0.	-9.029488	-0.675296	-0.080512
52.	1.	0.	-7.025865	-2.871375	0.840797
53.	1.	0.	-6.573904	-2.373114	2.482643
54.	1.	0.	-8.144171	-1.885366	1.820892
55.	1.	0.	-7.854056	1.383070	-1.954112
56.	1.	0.	4.139305	0.801496	4.179714
57.	1.	0.	5.198859	-0.001270	2.987960
58.	1.	0.	5.379635	1.746596	3.308590
59.	1.	0.	3.214820	3.560237	-2.741447
60.	1.	0.	1.738542	2.714111	-2.209585
61.	1.	0.	2.322543	4.128990	-1.298302
62.	1.	0.	8.398069	-2.077098	-1.201483
63.	1.	0.	8.561458	-0.312507	-1.159501
64.	1.	0.	8.610055	-1.233942	0.342948

Table S3. Gibbs free energies[a] and equilibrium populations[b] of low-energy conformers of 5*R*, 6*R*-**7**.

Conformers	In methanol	
	ΔG	*P* (%)
**7a**	0.21	22.58
**7b**	0	33.32
**7c**	1.38	8.87
**7d**	1.38	8.87
**7e**	0.19	26.35

[a]B3LYP/6-31+G (d, p), in kcal/mol. [b]From ΔG values at 298.15K.

Table S4**.** Cartesian coordinates for the low-energy reoptimized MMFF conformers of 5*R*, 6*R*-**7** at B3LYP/6-311+G (d, p) level of theory in methanol.

**7a**		Standard Orientation (Ångstroms)			
Center number	Atomic number	Atomic Type	X	Y	Z
1.	6.	0.	-2.493775	1.703531	0.166657
2.	6.	0.	-2.881627	0.422797	-0.211115
3.	6.	0.	-1.879183	-0.545930	-0.460590
4.	6.	0.	-0.516994	-0.206238	-0.316312
5.	6.	0.	-0.156757	1.099088	0.069889
6.	6.	0.	-1.143956	2.050007	0.309079
7.	6.	0.	0.530548	-1.191068	-0.567964
8.	6.	0.	1.941371	-0.807831	-0.396667
9.	6.	0.	2.345624	0.468502	-0.082633
10.	6.	0.	1.270255	1.478072	0.209342
11.	6.	0.	2.961277	-1.923094	-0.557553
12.	6.	0.	4.052598	-1.724273	0.486548
13.	6.	0.	4.658466	-0.345721	0.322537
14.	6.	0.	3.794081	0.764935	0.074098
15.	8.	0.	0.241548	-2.354255	-0.930144
16.	8.	0.	1.532029	2.622822	0.598964
17.	8.	0.	3.420160	-1.918332	1.750143
18.	8.	0.	-2.267996	-1.777043	-0.832335
19.	6.	0.	6.031371	-0.209824	0.393823
20.	6.	0.	6.654231	1.038811	0.201285
21.	6.	0.	5.840037	2.126509	-0.052009
22.	6.	0.	4.434721	2.025449	-0.110749
23.	8.	0.	3.820930	3.186702	-0.399791
24.	6.	0.	-7.094297	0.183686	0.485420
25.	6.	0.	-6.319374	-1.119650	0.656723
26.	6.	0.	-4.825876	-0.837209	0.823728
27.	6.	0.	-4.336674	0.034893	-0.353233
28.	8.	0.	-5.109832	1.227088	-0.438841
29.	6.	0.	-6.504109	1.000226	-0.673593
30.	8.	0.	-8.461389	-0.074330	0.208302
31.	6.	0.	-7.166321	2.358226	-0.839195
32.	8.	0.	-6.899681	-1.774422	1.788917
33.	6.	0.	4.315312	-1.995179	2.843699
34.	8.	0.	3.607813	-1.909439	-1.825153
35.	6.	0.	2.827422	-2.399738	-2.902696
36.	6.	0.	8.154676	1.171202	0.270819
37.	1.	0.	6.640449	-1.091922	0.578291

38.	1.	0.	-3.267365	2.442840	0.339226
39.	1.	0.	-0.853931	3.052498	0.601256
40.	1.	0.	2.465560	-2.884838	-0.388666
41.	1.	0.	4.831428	-2.485650	0.333359
42.	1.	0.	-1.431338	-2.303192	-0.970592
43.	1.	0.	6.258022	3.116379	-0.208126
44.	1.	0.	2.897990	3.147203	-0.049541
45.	1.	0.	-6.985127	0.769708	1.413823
46.	1.	0.	-6.483613	-1.726053	-0.249255
47.	1.	0.	-4.251953	-1.769984	0.857776
48.	1.	0.	-4.661532	-0.298601	1.764315
49.	1.	0.	-4.453367	-0.544502	-1.284278
50.	1.	0.	-6.624265	0.412557	-1.599430
51.	1.	0.	-8.738668	-0.732594	0.867840
52.	1.	0.	-6.727956	2.894491	-1.686437
53.	1.	0.	-8.238262	2.230137	-1.010566
54.	1.	0.	-7.025197	2.962063	0.064111
55.	1.	0.	-6.558764	-2.681403	1.823206
56.	1.	0.	3.706647	-2.184031	3.731672
57.	1.	0.	5.032408	-2.822555	2.719244
58.	1.	0.	4.878833	-1.063025	2.985723
59.	1.	0.	3.491231	-2.428786	-3.770733
60.	1.	0.	2.446347	-3.410347	-2.699823
61.	1.	0.	1.973434	-1.747520	-3.130665
62.	1.	0.	8.533147	0.873788	1.256958
63.	1.	0.	8.642405	0.522425	-0.467437
64.	1.	0.	8.476491	2.199523	0.082223

**7b**		Standard Orientation (Ångstroms)			
Center number	Atomic number	Atomic Type	X	Y	Z
1.	6.	0.	-2.538133	1.703034	0.187635
2.	6.	0.	-2.911565	0.419766	-0.196382
3.	6.	0.	-1.899978	-0.548832	-0.406193
4.	6.	0.	-0.544326	-0.204673	-0.222194
5.	6.	0.	-0.198322	1.103000	0.169375
6.	6.	0.	-1.194290	2.052372	0.374841
7.	6.	0.	0.512332	-1.190967	-0.421534

8.	6.	0.	1.916453	-0.791893	-0.225280
9.	6.	0.	2.310999	0.497958	0.032876
10.	6.	0.	1.222921	1.483428	0.357104
11.	6.	0.	2.920173	-1.907152	-0.390637
12.	6.	0.	4.142566	-1.635848	0.481464
13.	6.	0.	4.678579	-0.246335	0.217893
14.	6.	0.	3.757896	0.836863	0.077578
15.	8.	0.	0.242265	-2.365354	-0.757112
16.	8.	0.	1.471598	2.604751	0.817009
17.	8.	0.	3.689472	-1.816755	1.823526
18.	8.	0.	-2.273023	-1.785105	-0.777549
19.	6.	0.	6.046138	-0.069716	0.117678
20.	6.	0.	6.603813	1.196608	-0.141140
21.	6.	0.	5.733038	2.262398	-0.271923
22.	6.	0.	4.335290	2.119665	-0.157674
23.	8.	0.	3.658456	3.267789	-0.341806
24.	6.	0.	-7.143158	0.170358	0.371240
25.	6.	0.	-6.370250	-1.130546	0.566820
26.	6.	0.	-4.883685	-0.843358	0.780224
27.	6.	0.	-4.360765	0.028248	-0.382505
28.	8.	0.	-5.133885	1.218441	-0.493160
29.	6.	0.	-6.519987	0.988605	-0.769362
30.	8.	0.	-8.500422	-0.091531	0.052256
31.	6.	0.	-7.179919	2.345211	-0.954223
32.	8.	0.	-6.983831	-1.786993	1.680460
33.	6.	0.	4.715009	-1.808271	2.798973
34.	8.	0.	3.274051	-1.992746	-1.768522
35.	6.	0.	3.369532	-3.322098	-2.254281
36.	6.	0.	8.096472	1.373673	-0.264451
37.	1.	0.	6.699134	-0.932841	0.227232
38.	1.	0.	-3.318208	2.441890	0.330314
39.	1.	0.	-0.916040	3.056097	0.674298
40.	1.	0.	2.455171	-2.838544	-0.053277
41.	1.	0.	4.924669	-2.376132	0.250635
42.	1.	0.	-1.431626	-2.312148	-0.876580
43.	1.	0.	6.099814	3.266428	-0.462609
44.	1.	0.	2.785407	3.194580	0.113841
45.	1.	0.	-7.064374	0.756814	1.302468
46.	1.	0.	-6.504088	-1.737679	-0.343676
47.	1.	0.	-4.308292	-1.774243	0.833912
48.	1.	0.	-4.750403	-0.302300	1.724323
49.	1.	0.	-4.447780	-0.552639	-1.315909
50.	1.	0.	-6.611384	0.401016	-1.698541
51.	1.	0.	-8.795598	-0.751316	0.702457

52.	1.	0.	-6.718060	2.882578	-1.788189
53.	1.	0.	-8.246098	2.214861	-1.156984
54.	1.	0.	-7.066658	2.949238	-0.047124
55.	1.	0.	-6.640841	-2.692705	1.725798
56.	1.	0.	4.233327	-2.014536	3.758228
57.	1.	0.	5.465452	-2.590553	2.600384
58.	1.	0.	5.227836	-0.838771	2.855305
59.	1.	0.	3.655230	-3.248776	-3.306858
60.	1.	0.	4.136513	-3.904348	-1.720756
61.	1.	0.	2.406147	-3.843941	-2.174686
62.	1.	0.	8.611568	1.052144	0.649428
63.	1.	0.	8.498219	0.768975	-1.087282
64.	1.	0.	8.362524	2.417877	-0.452499

**7c**		Standard Orientation (Ångstroms)			
Center number	Atomic number	Atomic Type	X	Y	Z
1.	6.	0.	2.470708	1.728892	-0.202801
2.	6.	0.	2.850937	0.453470	0.199968
3.	6.	0.	1.842895	-0.505721	0.462665
4.	6.	0.	0.482726	-0.161542	0.309202
5.	6.	0.	0.130288	1.138096	-0.102208
6.	6.	0.	1.123192	2.079234	-0.356320
7.	6.	0.	-0.571103	-1.135196	0.576937
8.	6.	0.	-1.978819	-0.745698	0.403679
9.	6.	0.	-2.377596	0.523097	0.050753
10.	6.	0.	-1.294346	1.523007	-0.252795
11.	6.	0.	-3.005036	-1.845178	0.604282
12.	6.	0.	-4.094548	-1.687113	-0.457801
13.	6.	0.	-4.683854	-0.299061	-0.366440
14.	6.	0.	-3.821951	0.816357	-0.132338
15.	8.	0.	-0.287374	-2.296745	0.952607
16.	8.	0.	-1.545603	2.664576	-0.656613
17.	8.	0.	-3.570664	-1.870714	-1.772225
18.	8.	0.	2.224182	-1.732388	0.856893
19.	6.	0.	-6.051262	-0.161265	-0.497682
20.	6.	0.	-6.674123	1.094988	-0.374654
21.	6.	0.	-5.865660	2.188130	-0.123988

22.	6.	0.	-4.464786	2.085559	-0.012064
23.	8.	0.	-3.858040	3.254258	0.261051
24.	6.	0.	7.066336	0.187572	-0.470314
25.	6.	0.	6.287852	-1.115981	-0.623678
26.	6.	0.	4.796027	-0.831708	-0.803050
27.	6.	0.	4.303701	0.062215	0.356111
28.	8.	0.	5.080751	1.252849	0.425413
29.	6.	0.	6.472891	1.025236	0.671690
30.	8.	0.	8.431035	-0.070345	-0.181731
31.	6.	0.	7.138918	2.383580	0.818358
32.	8.	0.	6.871630	-1.791735	-1.741706
33.	6.	0.	-3.398991	-3.224810	-2.150755
34.	8.	0.	-3.666394	-1.772978	1.863651
35.	6.	0.	-2.887473	-2.186538	2.973895
36.	6.	0.	-8.169662	1.230625	-0.509913
37.	1.	0.	-6.655595	-1.045799	-0.683326
38.	1.	0.	3.248461	2.461143	-0.386310
39.	1.	0.	0.839286	3.077385	-0.668566
40.	1.	0.	-2.504901	-2.814725	0.504649
41.	1.	0.	-4.880520	-2.428797	-0.256662
42.	1.	0.	1.383790	-2.251241	1.000837
43.	1.	0.	-6.285075	3.183728	-0.015971
44.	1.	0.	-2.924153	3.203617	-0.056692
45.	1.	0.	6.963856	0.758306	-1.408918
46.	1.	0.	6.445568	-1.707396	0.293288
47.	1.	0.	4.219256	-1.763159	-0.823805
48.	1.	0.	4.638173	-0.309077	-1.753717
49.	1.	0.	4.413246	-0.501581	1.297565
50.	1.	0.	6.585871	0.452685	1.607836
51.	1.	0.	8.709784	-0.739918	-0.829150
52.	1.	0.	6.697994	2.935274	1.654281
53.	1.	0.	8.209497	2.254704	0.997448
54.	1.	0.	7.004625	2.972883	-0.095520
55.	1.	0.	6.530192	-2.698951	-1.760623
56.	1.	0.	-3.092188	-3.218069	-3.199910
57.	1.	0.	-2.624260	-3.737867	-1.562270
58.	1.	0.	-4.339007	-3.790734	-2.057131
59.	1.	0.	-3.555804	-2.172094	3.838799
60.	1.	0.	-2.491597	-3.202624	2.835160
61.	1.	0.	-2.043869	-1.509472	3.166220
62.	1.	0.	-8.508743	0.894916	-1.497980
63.	1.	0.	-8.690631	0.614123	0.233332
64.	1.	0.	-8.493028	2.267027	-0.376027

**7d**		Standard Orientation (Ångstroms)			
Center number	Atomic number	Atomic Type	X	Y	Z
1.	6.	0.	2.470675	1.728924	-0.202729
2.	6.	0.	2.850922	0.453507	0.200037
3.	6.	0.	1.842886	-0.505655	0.462884
4.	6.	0.	0.482708	-0.161462	0.309537
5.	6.	0.	0.130253	1.138170	-0.101876
6.	6.	0.	1.123148	2.079287	-0.356107
7.	6.	0.	-0.571111	-1.135109	0.577356
8.	6.	0.	-1.978827	-0.745635	0.404078
9.	6.	0.	-2.377629	0.523133	0.051092
10.	6.	0.	-1.294388	1.523123	-0.252288
11.	6.	0.	-3.005024	-1.845150	0.604582
12.	6.	0.	-4.094320	-1.687210	-0.457751
13.	6.	0.	-4.683733	-0.299184	-0.366622
14.	6.	0.	-3.821967	0.816297	-0.132249
15.	8.	0.	-0.287370	-2.296630	0.953087
16.	8.	0.	-1.545658	2.664784	-0.655824
17.	8.	0.	-3.570122	-1.870900	-1.772035
18.	8.	0.	2.224202	-1.732300	0.857161
19.	6.	0.	-6.051100	-0.161463	-0.498342
20.	6.	0.	-6.674078	1.094764	-0.375558
21.	6.	0.	-5.865768	2.187944	-0.124590
22.	6.	0.	-4.464929	2.085437	-0.012113
23.	8.	0.	-3.858368	3.254141	0.261410
24.	6.	0.	7.066266	0.187510	-0.470624
25.	6.	0.	6.287741	-1.116038	-0.623885
26.	6.	0.	4.795902	-0.831742	-0.803119
27.	6.	0.	4.303699	0.062233	0.356060
28.	8.	0.	5.080782	1.252845	0.425258
29.	6.	0.	6.472935	1.025201	0.671418
30.	8.	0.	8.430981	-0.070423	-0.182162
31.	6.	0.	7.139008	2.383532	0.818004
32.	8.	0.	6.871391	-1.791819	-1.741949
33.	6.	0.	-3.398436	-3.225017	-2.150468
34.	8.	0.	-3.666673	-1.772934	1.863788
35.	6.	0.	-2.887677	-2.185585	2.974304
36.	6.	0.	-8.169575	1.230283	-0.511334
37.	1.	0.	-6.655329	-1.046030	-0.684169
38.	1.	0.	3.248421	2.461155	-0.386349
39.	1.	0.	0.839226	3.077438	-0.668342
40.	1.	0.	-2.504834	-2.814691	0.505103

41.	1.	0.	-4.880299	-2.428920	-0.256731
42.	1.	0.	1.383835	-2.251139	1.001238
43.	1.	0.	-6.285274	3.183520	-0.016714
44.	1.	0.	-2.924377	3.203622	-0.056023
45.	1.	0.	6.963707	0.758220	-1.409234
46.	1.	0.	6.445532	-1.707437	0.293080
47.	1.	0.	4.219115	-1.763184	-0.823779
48.	1.	0.	4.637960	-0.309145	-1.753790
49.	1.	0.	4.413317	-0.501530	1.297525
50.	1.	0.	6.585985	0.452670	1.607567
51.	1.	0.	8.709683	-0.739971	-0.829626
52.	1.	0.	6.698134	2.935264	1.653926
53.	1.	0.	8.209593	2.254640	0.997053
54.	1.	0.	7.004694	2.972812	-0.095885
55.	1.	0.	6.529813	-2.698982	-1.760905
56.	1.	0.	-3.091568	-3.218364	-3.199606
57.	1.	0.	-2.623744	-3.738033	-1.561894
58.	1.	0.	-4.338459	-3.790931	-2.056865
59.	1.	0.	-3.556090	-2.171013	3.839141
60.	1.	0.	-2.491316	-3.201559	2.836124
61.	1.	0.	-2.044418	-1.508017	3.166389
62.	1.	0.	-8.508348	0.894145	-1.499359
63.	1.	0.	-8.690731	0.614055	0.232012
64.	1.	0.	-8.493035	2.266720	-0.377952

**7e**		Standard Orientation (Ångstroms)			
Center number	Atomic number	Atomic Type	X	Y	Z
1.	6.	0.	-2.496895	1.688530	0.172106
2.	6.	0.	-2.882032	0.408096	-0.208747
3.	6.	0.	-1.877177	-0.557760	-0.461359
4.	6.	0.	-0.515705	-0.214669	-0.315877
5.	6.	0.	-0.158618	1.090722	0.072854
6.	6.	0.	-1.148006	2.038631	0.314451
7.	6.	0.	0.534362	-1.196327	-0.569152
8.	6.	0.	1.944526	-0.809648	-0.395536
9.	6.	0.	2.345222	0.467586	-0.081728
10.	6.	0.	1.267500	1.473745	0.211448
11.	6.	0.	2.967191	-1.922739	-0.554514
12.	6.	0.	4.058129	-1.719494	0.489284
13.	6.	0.	4.660606	-0.339676	0.323268
14.	6.	0.	3.793253	0.768140	0.074029

15.	8.	0.	0.249450	-2.358958	-0.934760
16.	8.	0.	1.526392	2.619227	0.601302
17.	8.	0.	3.426117	-1.912882	1.753015
18.	8.	0.	-2.263845	-1.786742	-0.837847
19.	6.	0.	6.033338	-0.199902	0.393207
20.	6.	0.	6.652400	1.050090	0.198439
21.	6.	0.	5.834880	2.135270	-0.055710
22.	6.	0.	4.429935	2.030008	-0.113256
23.	8.	0.	3.811783	3.188961	-0.403762
24.	6.	0.	-7.084727	0.181717	0.487028
25.	6.	0.	-6.317580	-1.122889	0.690006
26.	6.	0.	-4.826866	-0.835579	0.839301
27.	6.	0.	-4.335761	0.014827	-0.349347
28.	8.	0.	-5.108636	1.210403	-0.464743
29.	6.	0.	-6.498996	0.972710	-0.696450
30.	8.	0.	-8.451198	-0.187434	0.271479
31.	6.	0.	-7.157069	2.328667	-0.905202
32.	8.	0.	-6.776816	-1.806851	1.844157
33.	6.	0.	4.321137	-1.986824	2.846813
34.	8.	0.	3.613127	-1.909623	-1.822492
35.	6.	0.	2.837628	-2.414380	-2.896945
36.	6.	0.	8.152553	1.187016	0.266870
37.	1.	0.	6.644968	-1.080094	0.578380
38.	1.	0.	-3.271567	2.426021	0.347832
39.	1.	0.	-0.860301	3.041115	0.608925
40.	1.	0.	2.473807	-2.885313	-0.383667
41.	1.	0.	4.838775	-2.479258	0.337207
42.	1.	0.	-1.428368	-2.314309	-0.973734
43.	1.	0.	6.250053	3.126066	-0.213585
44.	1.	0.	2.890008	3.146974	-0.050721
45.	1.	0.	-6.986125	0.784933	1.402615
46.	1.	0.	-6.480084	-1.742614	-0.210019
47.	1.	0.	-4.266350	-1.773394	0.890784
48.	1.	0.	-4.656985	-0.285521	1.772528
49.	1.	0.	-4.449096	-0.577916	-1.271576
50.	1.	0.	-6.619323	0.360668	-1.606562
51.	1.	0.	-9.010521	0.576491	0.475125
52.	1.	0.	-6.689900	2.852205	-1.744550
53.	1.	0.	-8.222245	2.207797	-1.129561
54.	1.	0.	-7.049095	2.951806	-0.009772
55.	1.	0.	-7.744387	-1.842256	1.753895
56.	1.	0.	3.712561	-2.175753	3.734801
57.	1.	0.	5.039730	-2.813074	2.723463
58.	1.	0.	4.882976	-1.053456	2.987814

59.	1.	0.	3.501537	-2.441243	-3.765014
60.	1.	0.	2.467605	-3.427875	-2.688179
61.	1.	0.	1.976400	-1.773028	-3.128225
62.	1.	0.	8.532112	0.895882	1.254486
63.	1.	0.	8.642024	0.535825	-0.468083
64.	1.	0.	8.471430	2.215282	0.072920

Figure S1. ^{1}H NMR (500 MHz) spectrum of compound **1** in $CDCl_3/CD_3OD$.

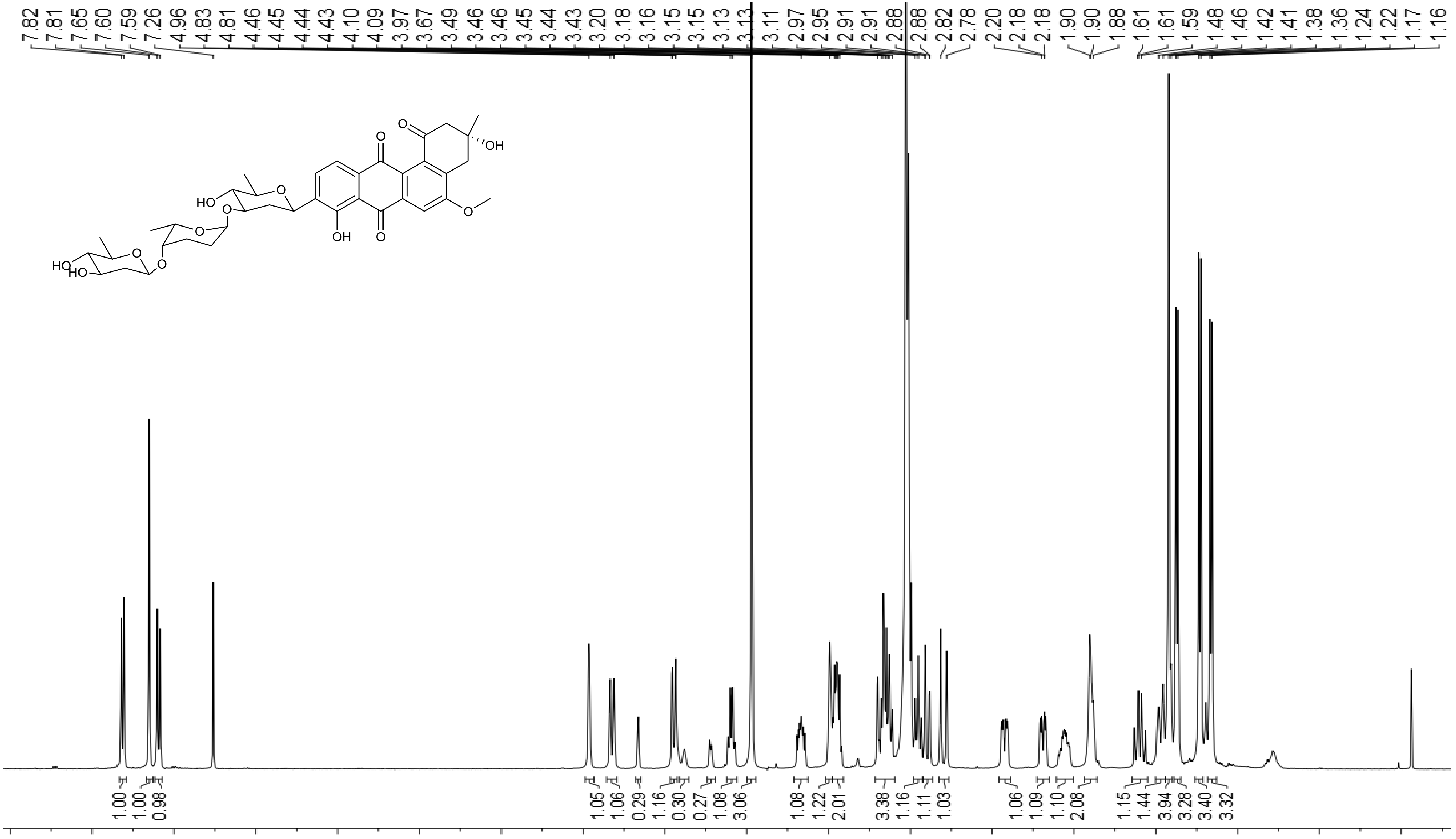

Figure S2. ^{13}C NMR (125 MHz) spectrum of compound **1** in $CDCl_3/CD_3OD$.

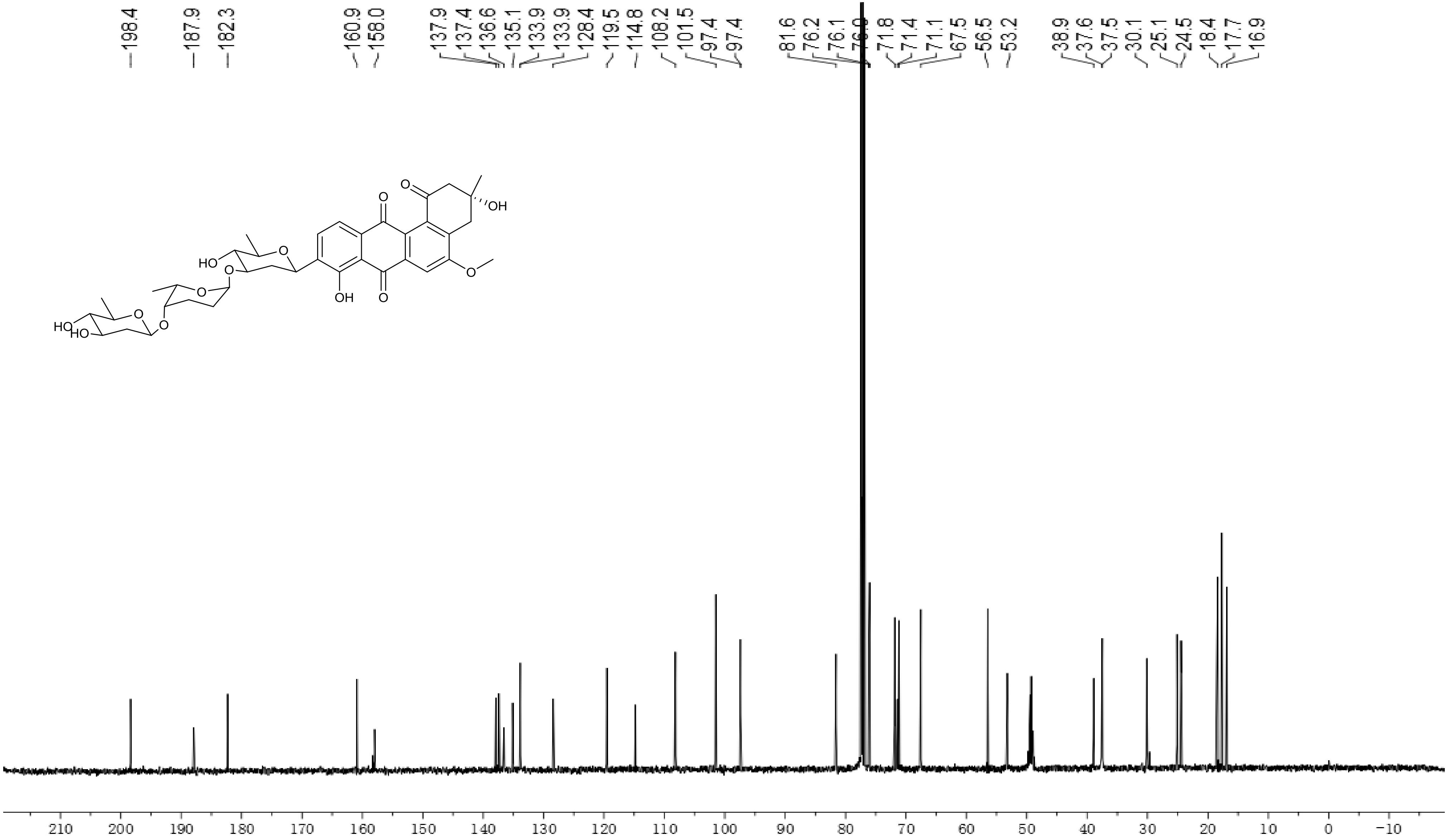

Figure S3. ^{13}C DEPT spectrum of compound **1** in $CDCl_3/CD_3OD$.

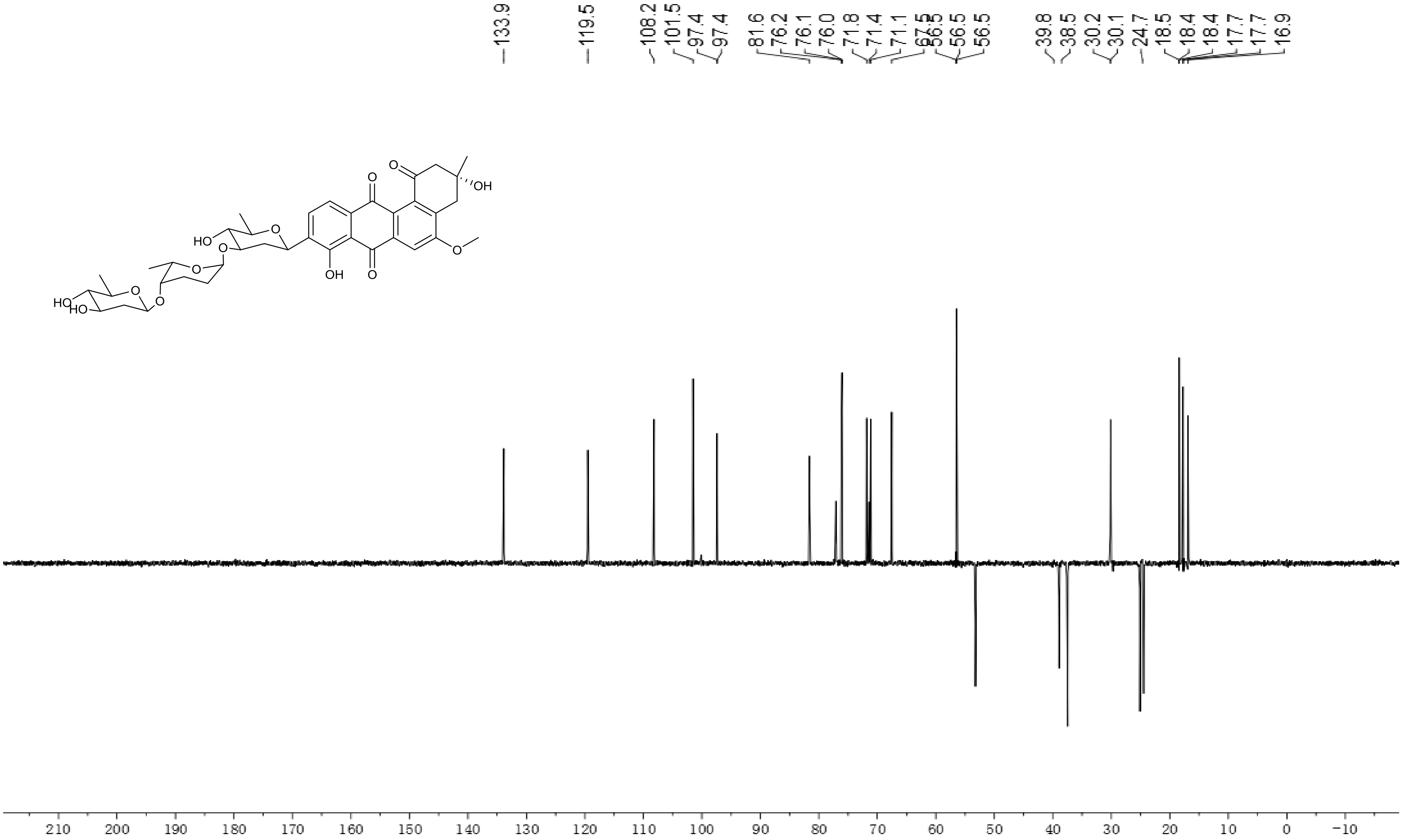

Figure S4. ^1H-^1H COSY spectrum of compound **1** in $CDCl_3/CD_3OD$.

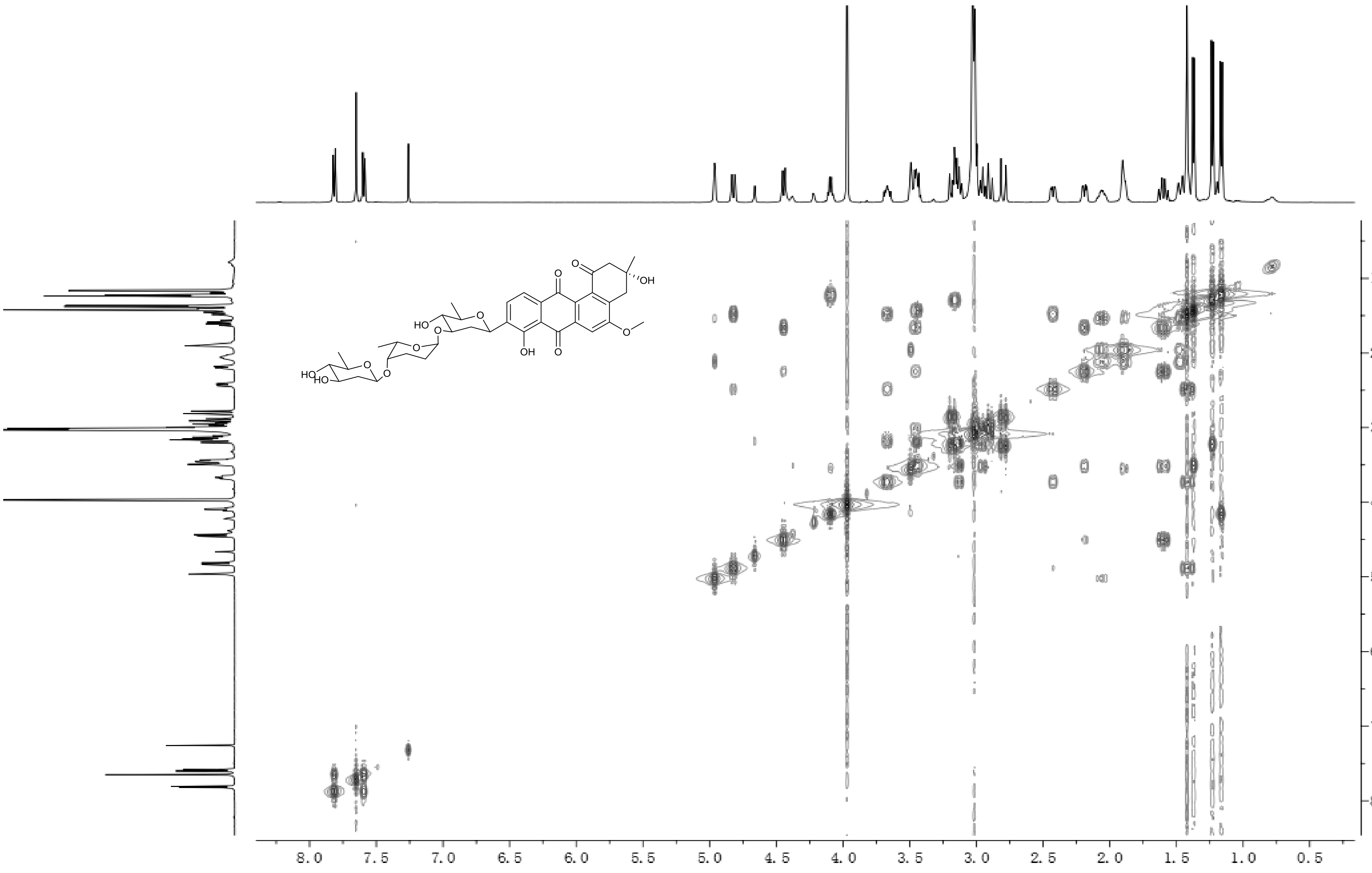

Figure S5. HSQC spectrum of compound **1** in $CDCl_3/CD_3OD$.

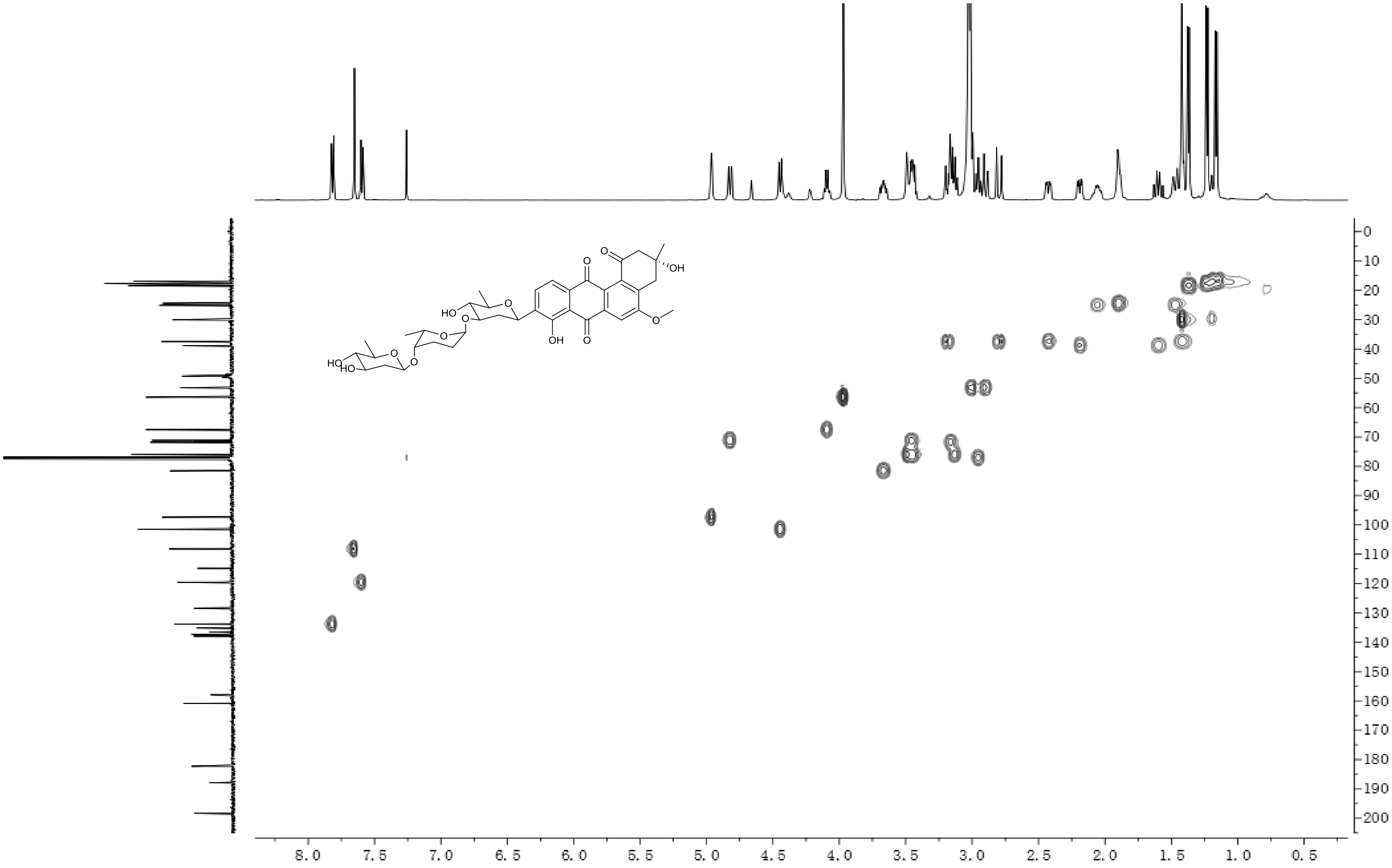

Figure S6. HMBC spectrum of compound **1** in $CDCl_3/CD_3OD$.

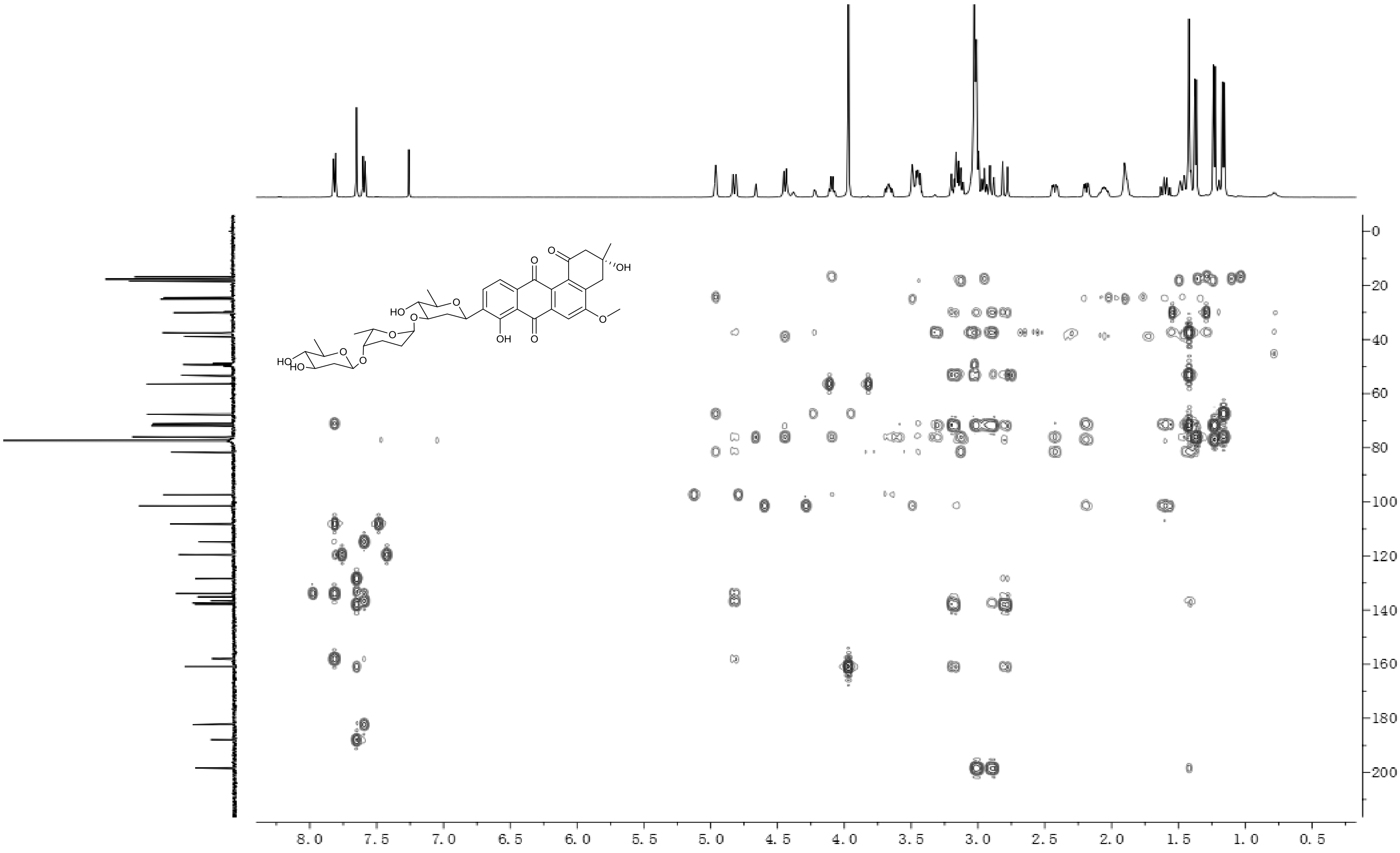

Figure S7. NOESY spectrum of compound **1** in $CDCl_3/CD_3OD$.

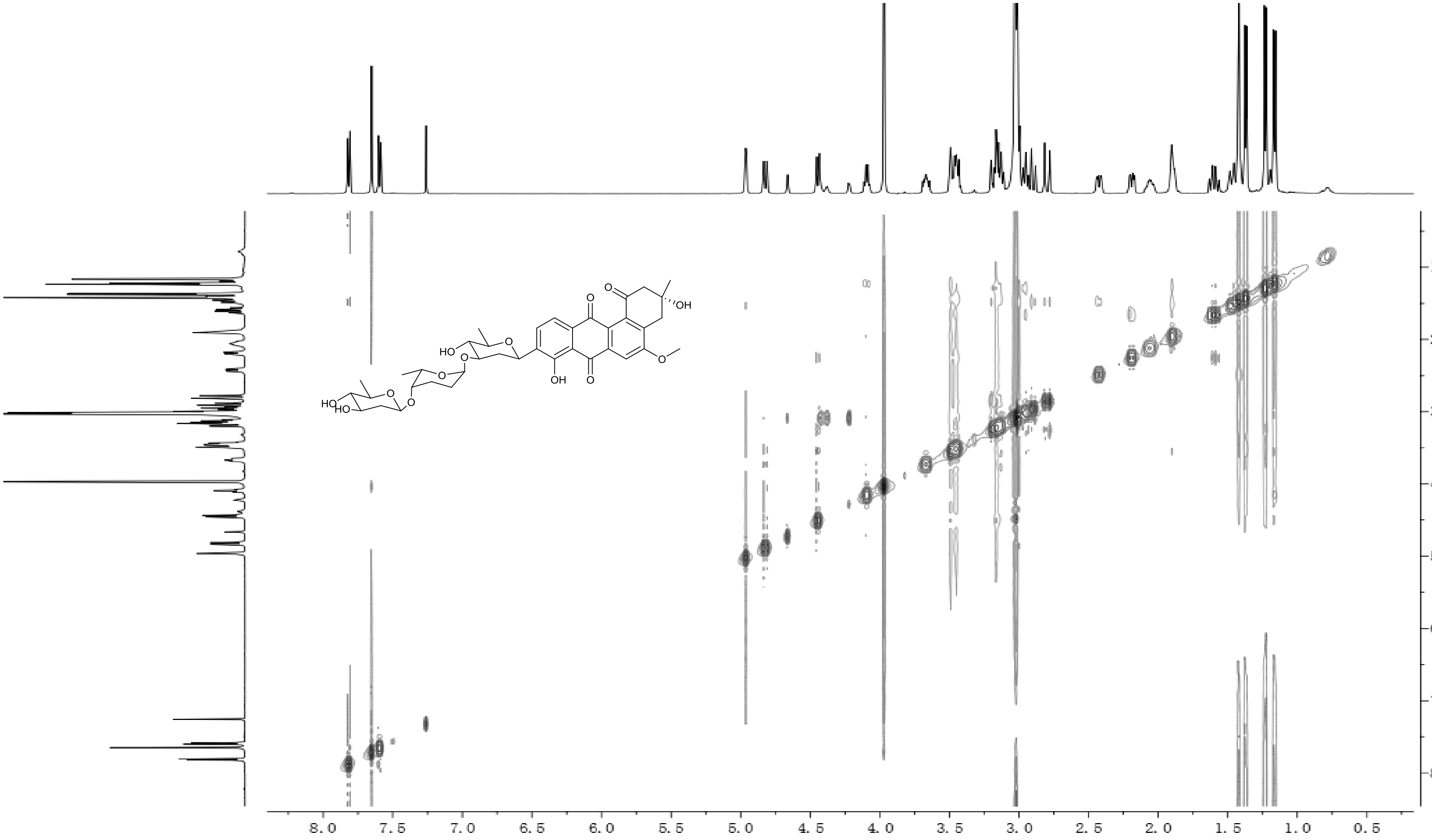

Figure S8. ^{1}H NMR (700 MHz) spectrum of compound **2** in DMSO-d_6.

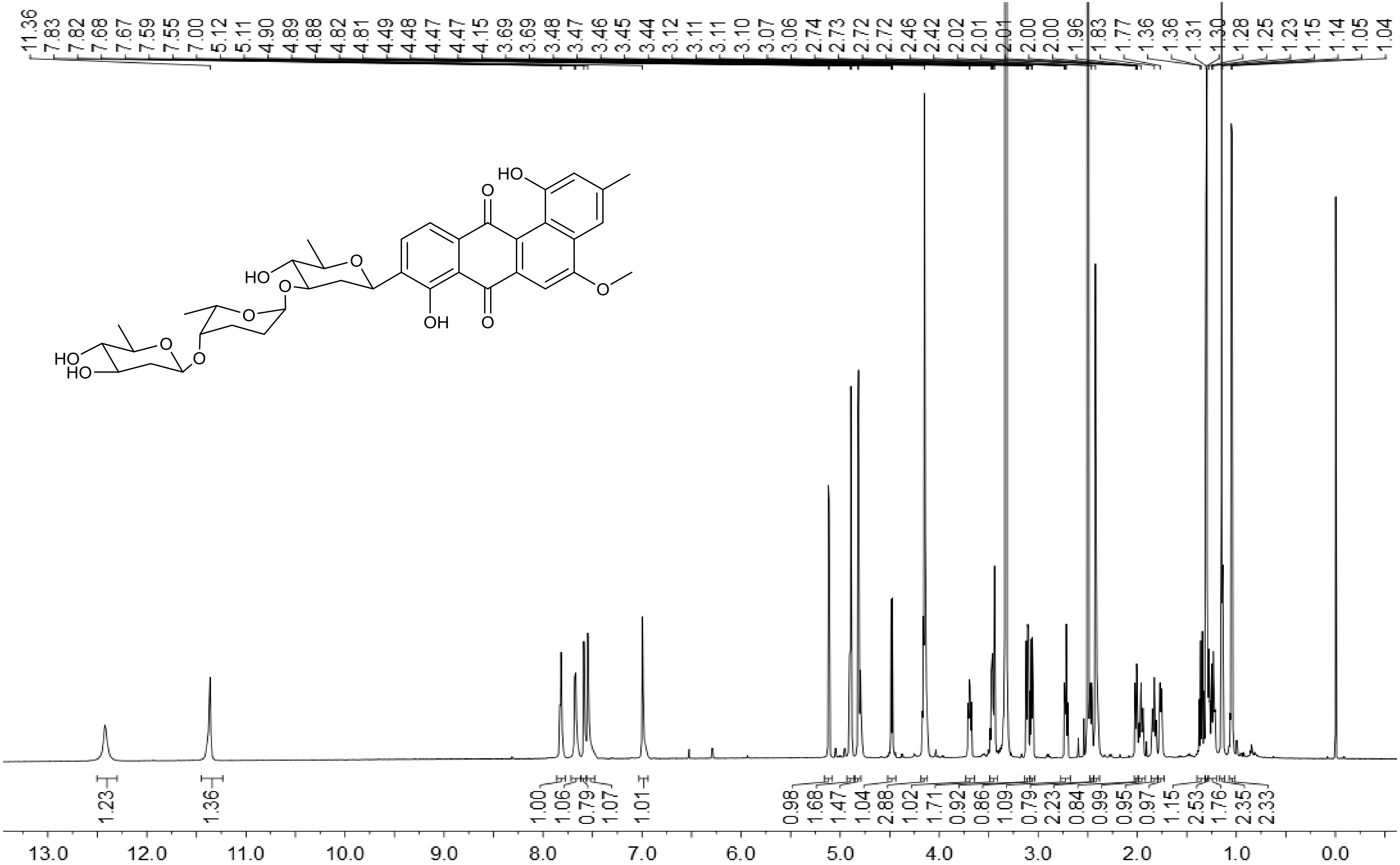

Figure S9. ^{13}C NMR (176 MHz) spectrum of compound **2** in DMSO-d_6.

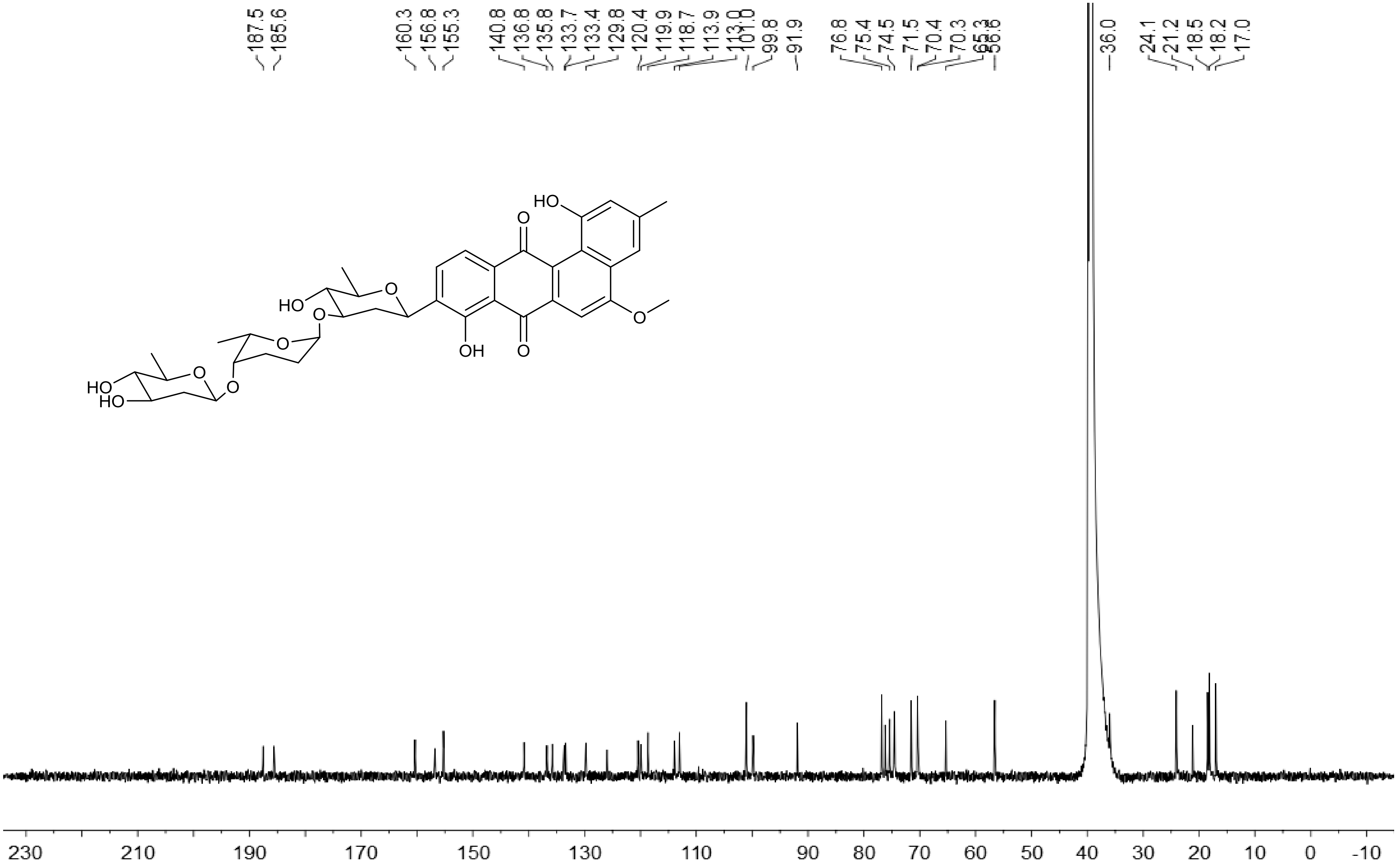

Figure S10. ^{13}C DEPT spectrum of compound **2** in DMSO-d_6..

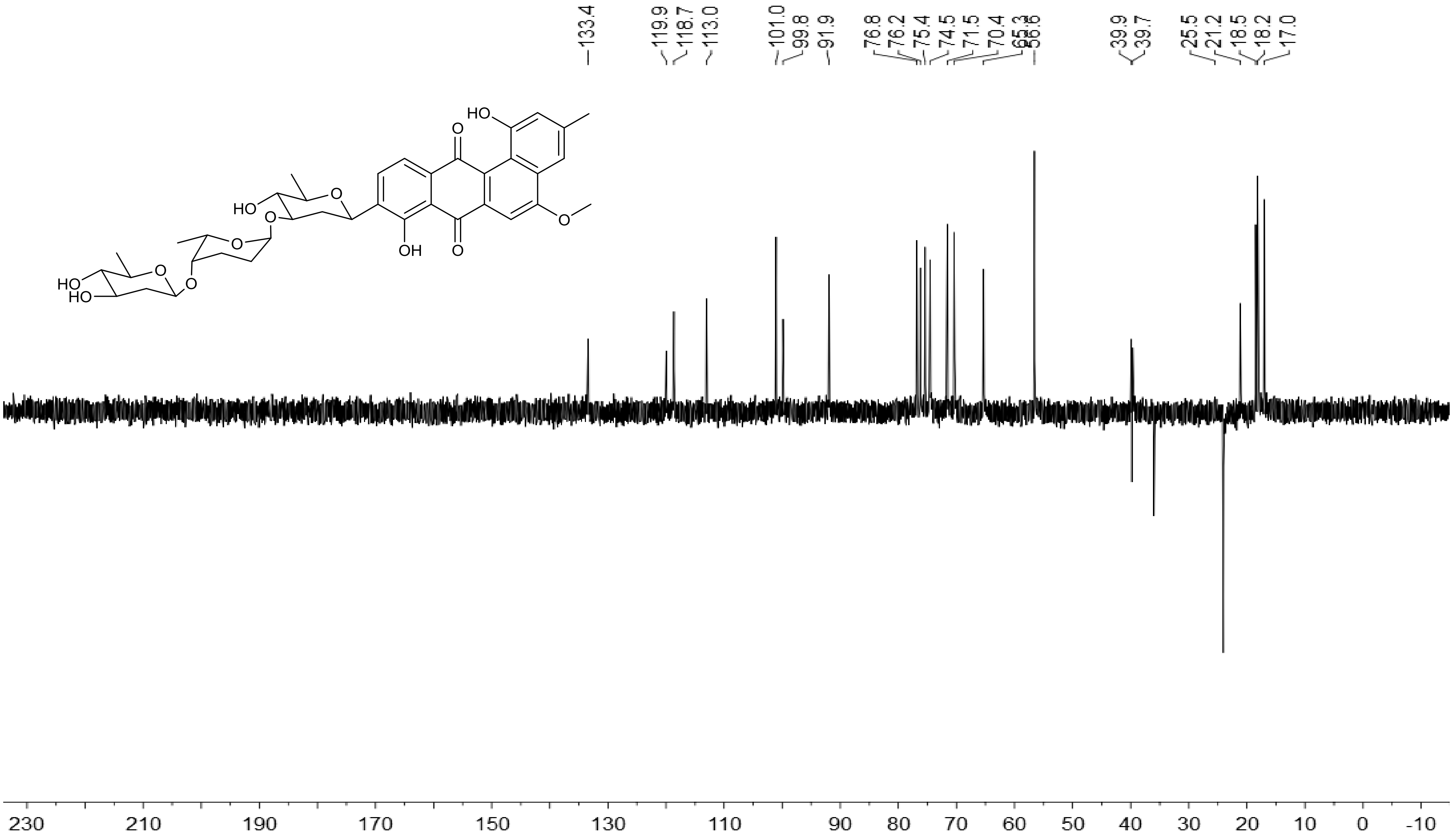

Figure S11. ^{1}H-^{1}H COSY spectrum of compound **2** in DMSO-d_6.

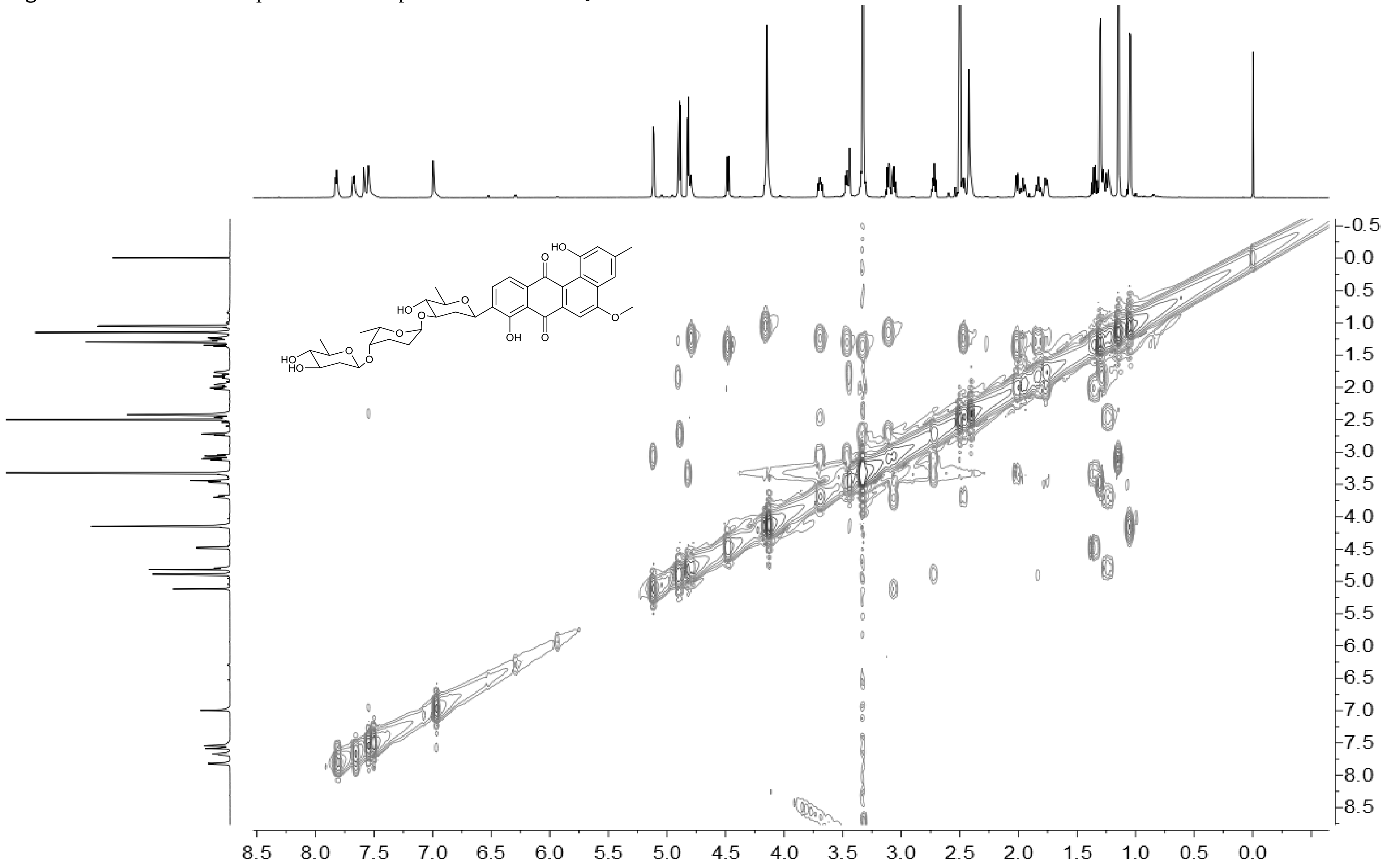

Figure S12. HSQC spectrum of compound **2** in DMSO-d_6.

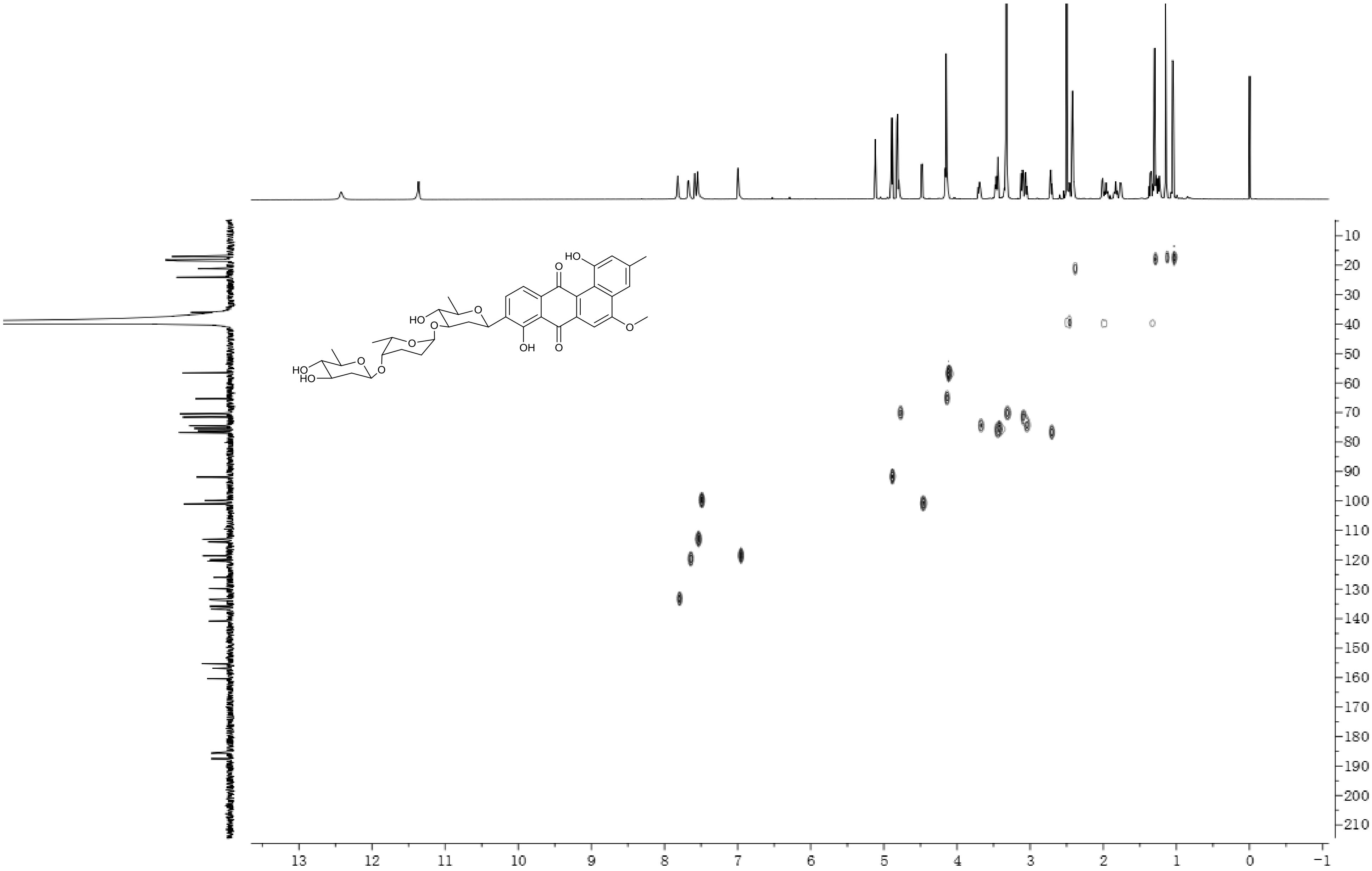

Figure S13. HMBC spectrum of compound **2** in DMSO-d_6.

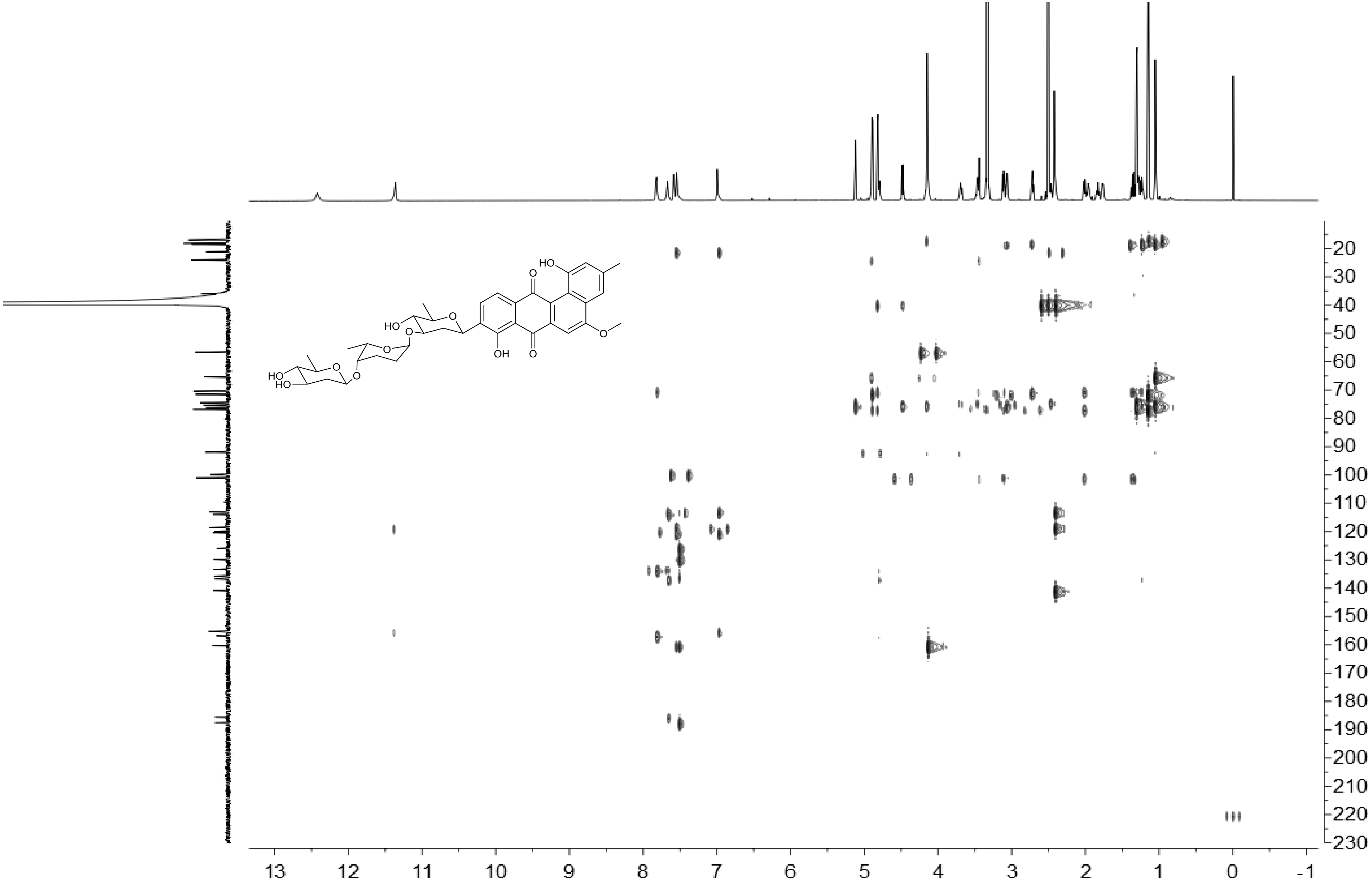

Figure S14. NOESY spectrum of compound **2** in DMSO-d_6.

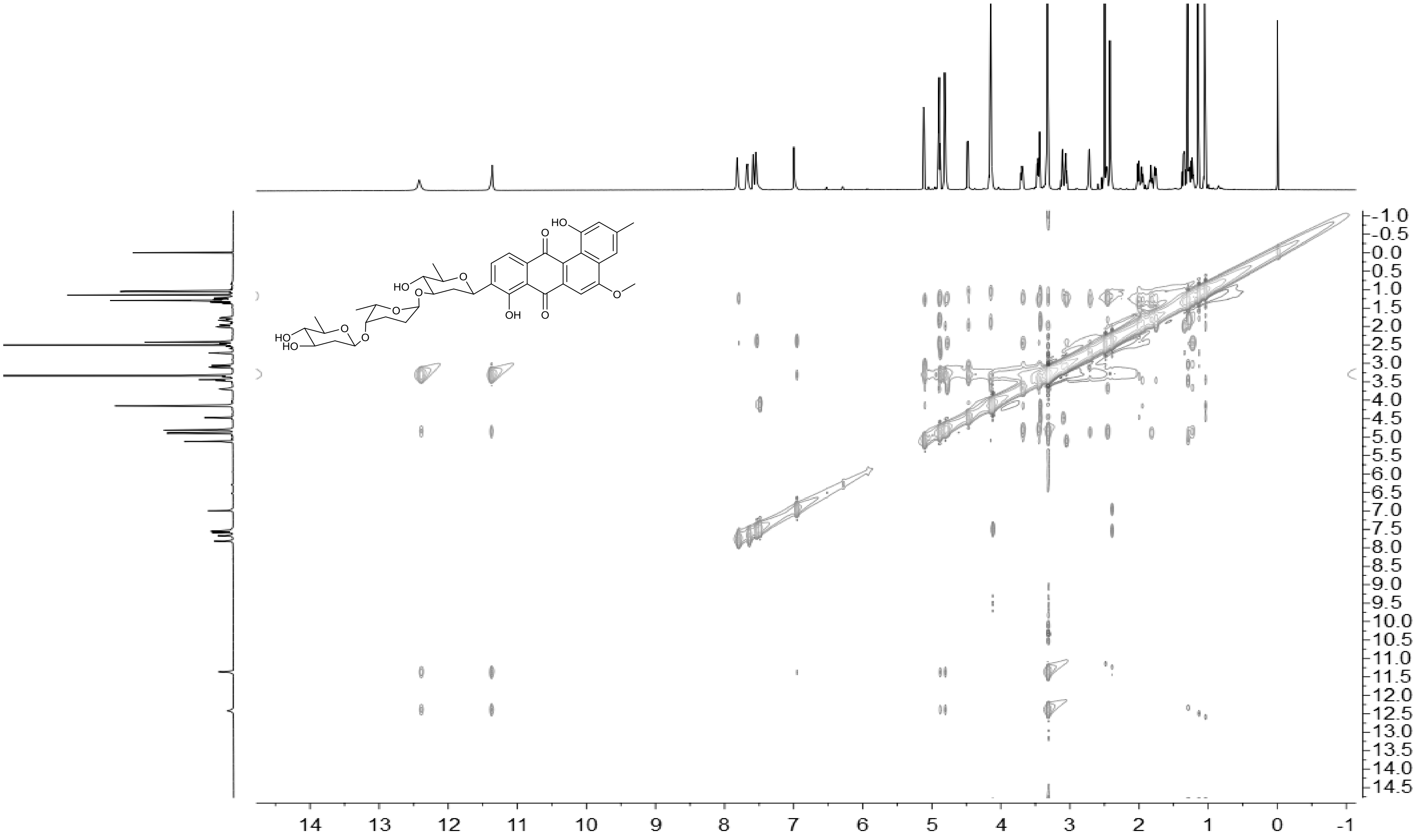

Figure S15. ^{1}H NMR (700 MHz) spectrum of compound **3** in DMSO-d_6/CD_3OD.

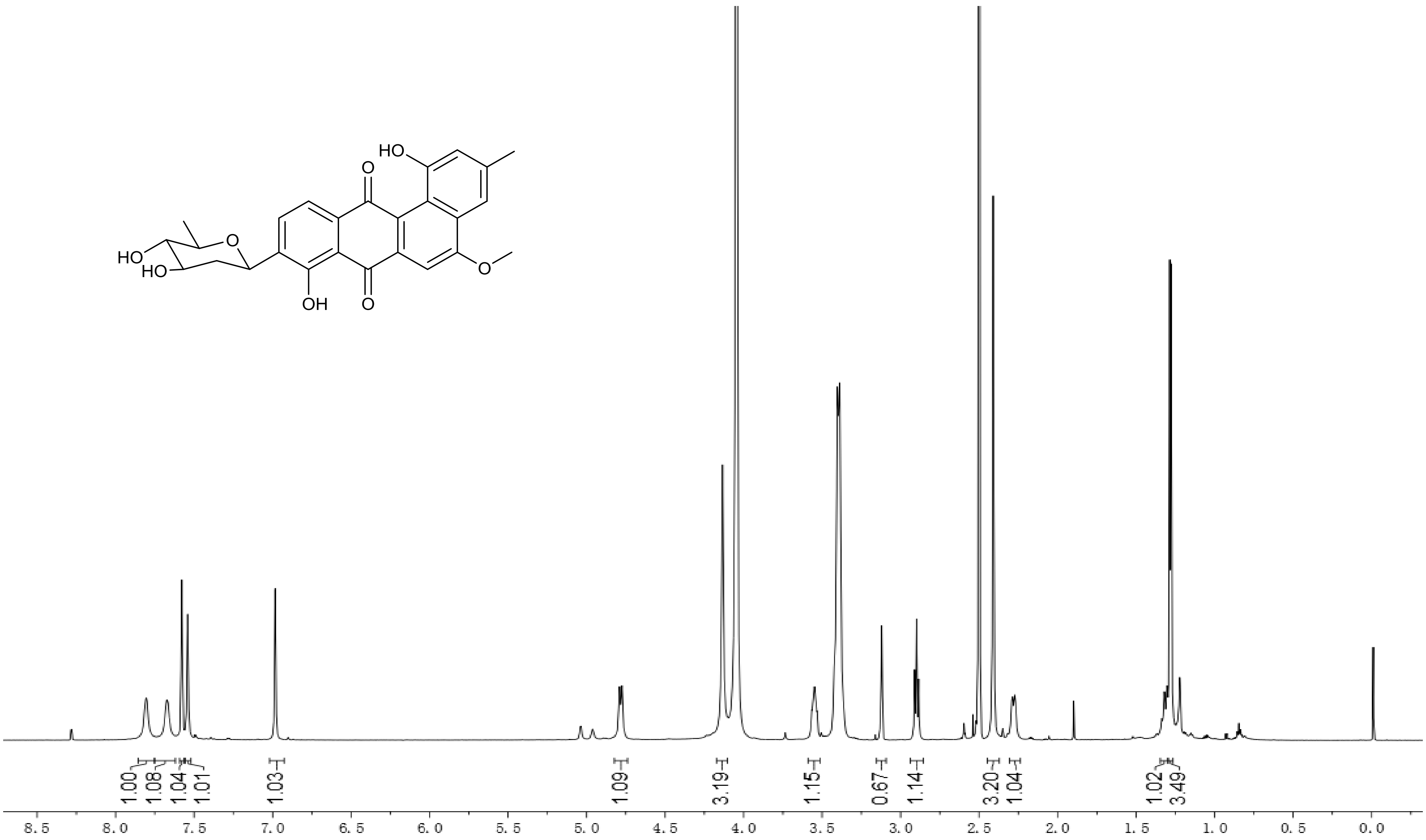

Figure S16. ^{13}C NMR (176 MHz) spectrum of compound **3** in DMSO-d_6/CD_3OD.

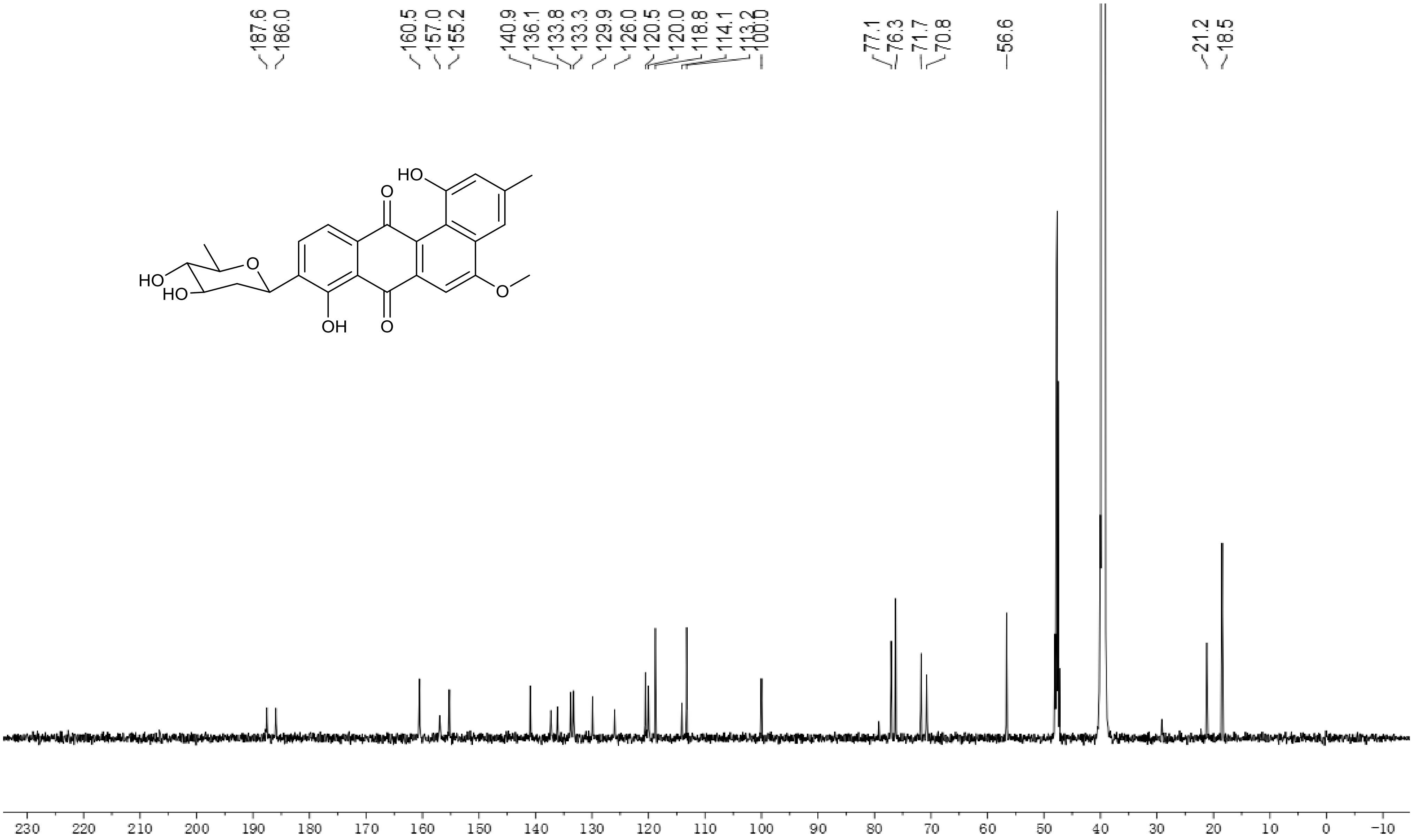

Figure S17. ^{13}C DEPT spectrum of compound **3** in DMSO-d_6/CD_3OD.

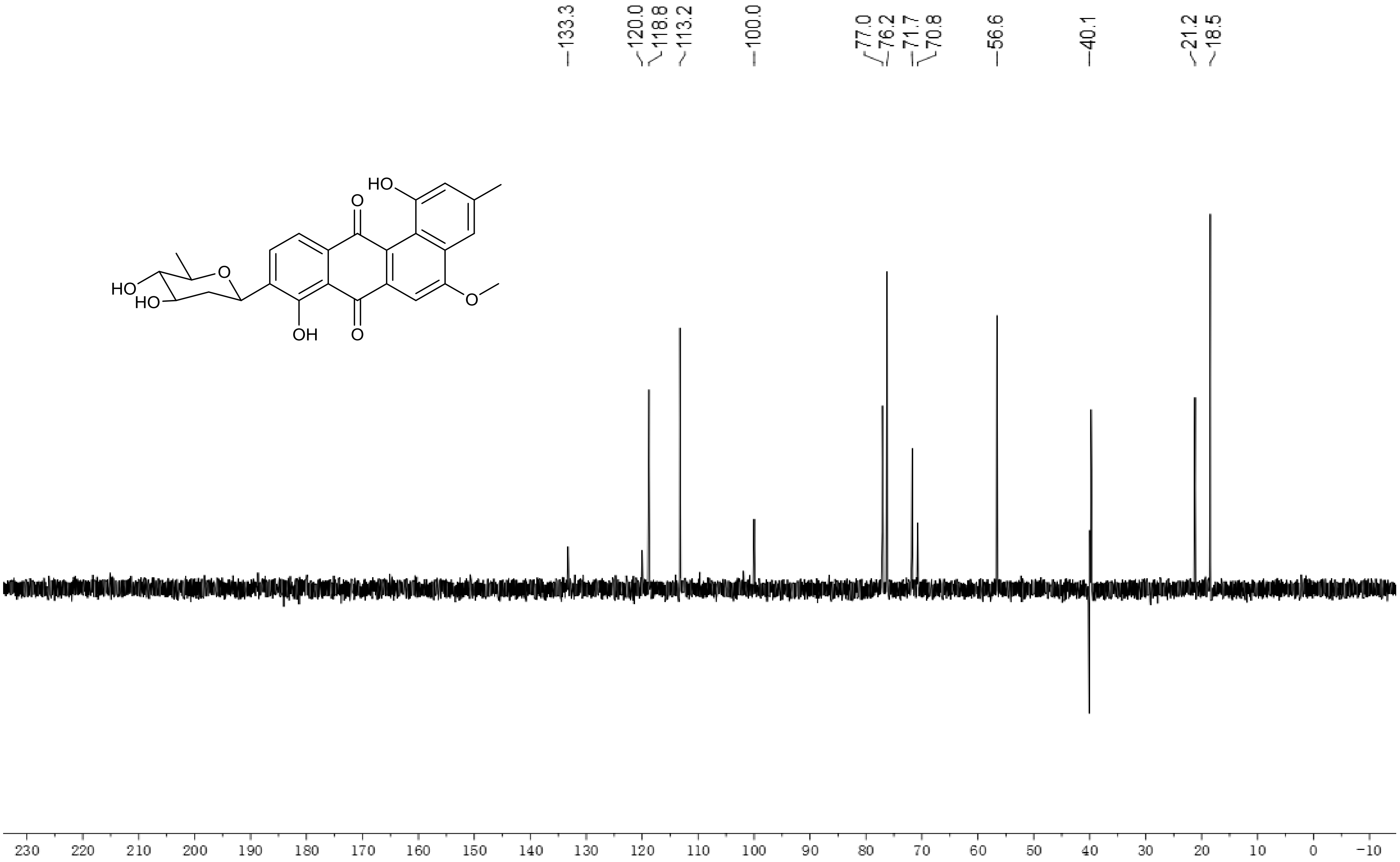

Figure S18. ^{1}H-^{1}H COSY spectrum of compound **3** in DMSO-d_6/CD_3OD.

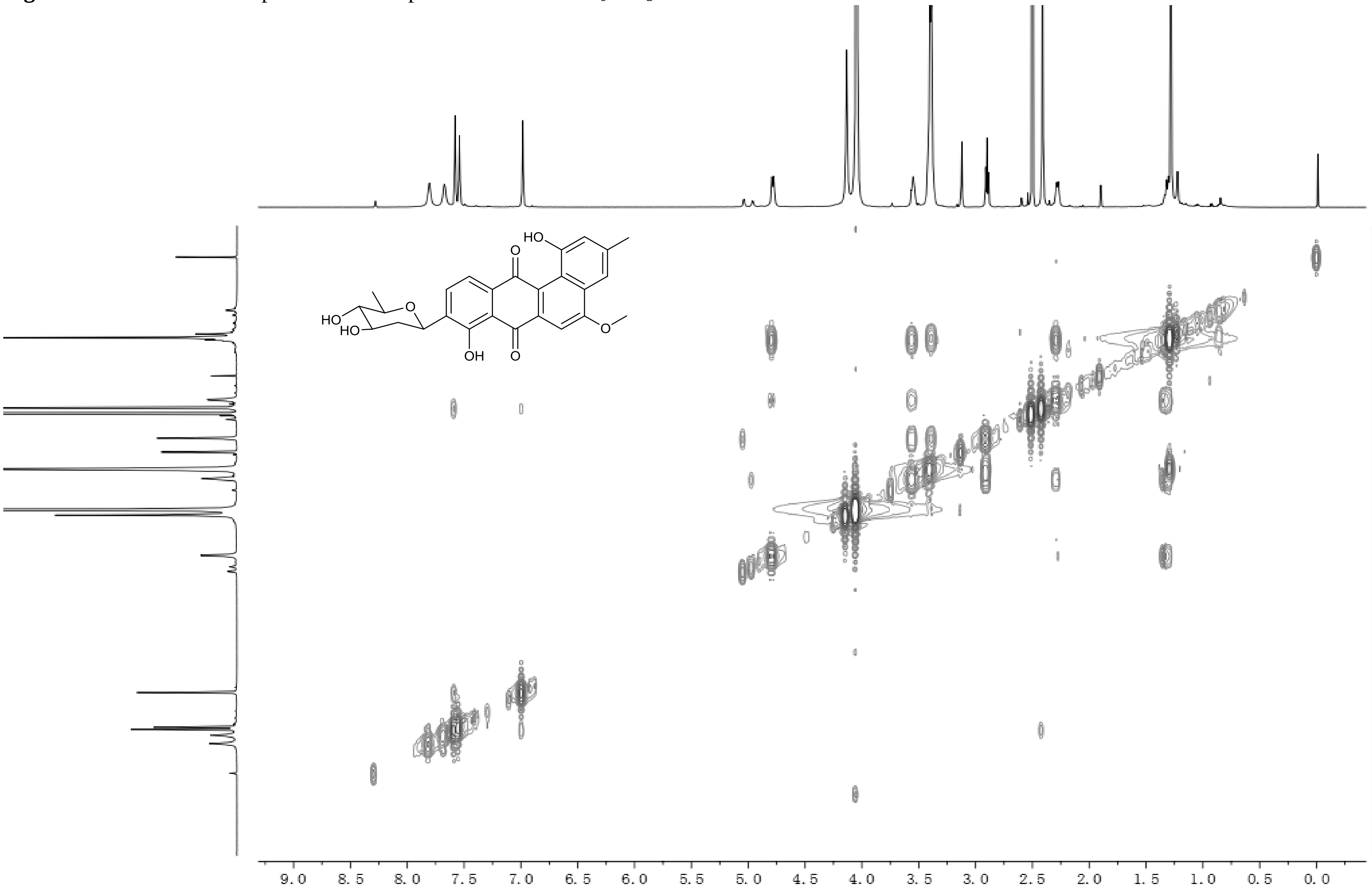

Figure S19. HSQC spectrum of compound **3** in DMSO-d_6/CD_3OD.

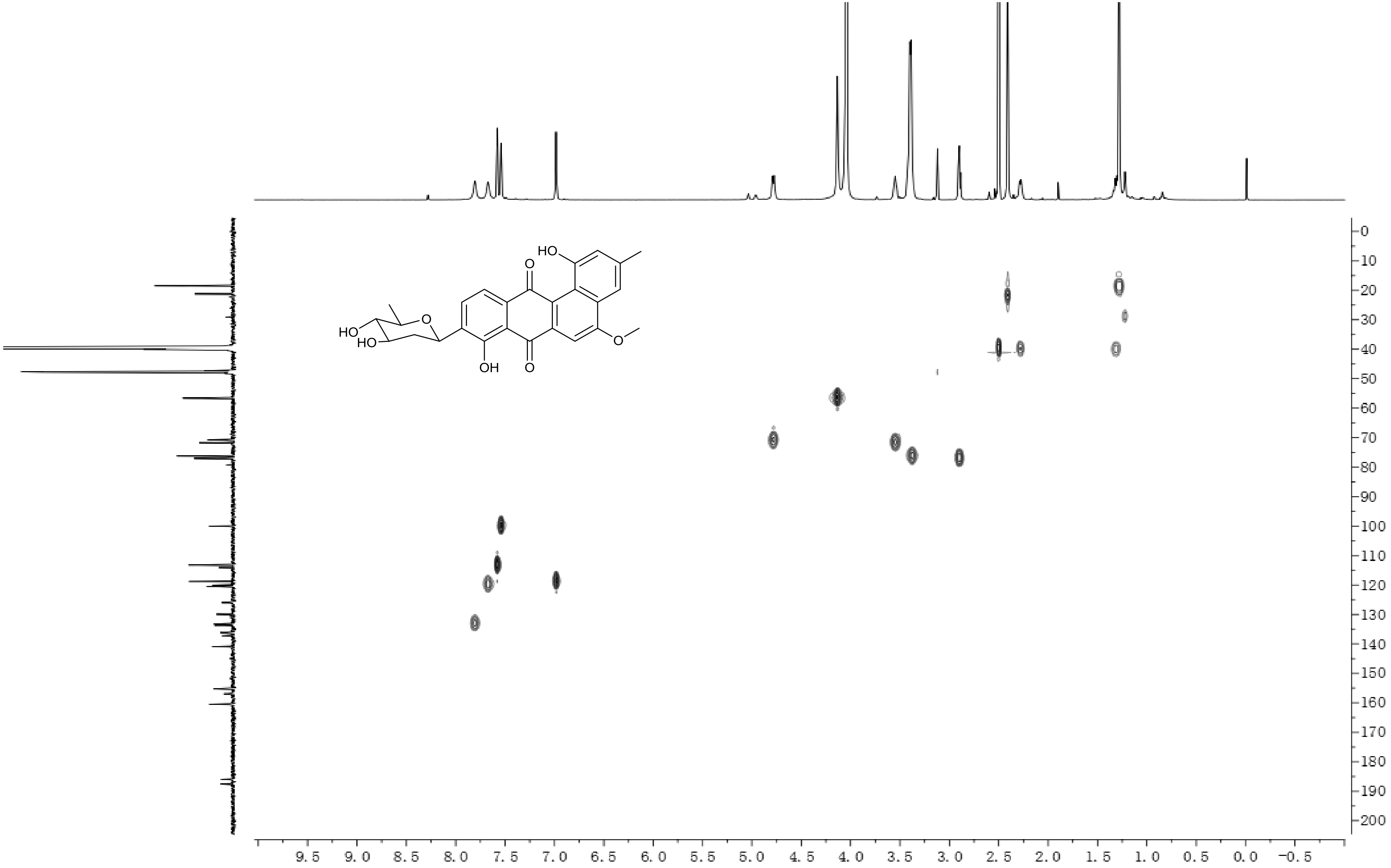

Figure S20. HMBC spectrum of compound **3** in DMSO-d_6/CD_3OD.

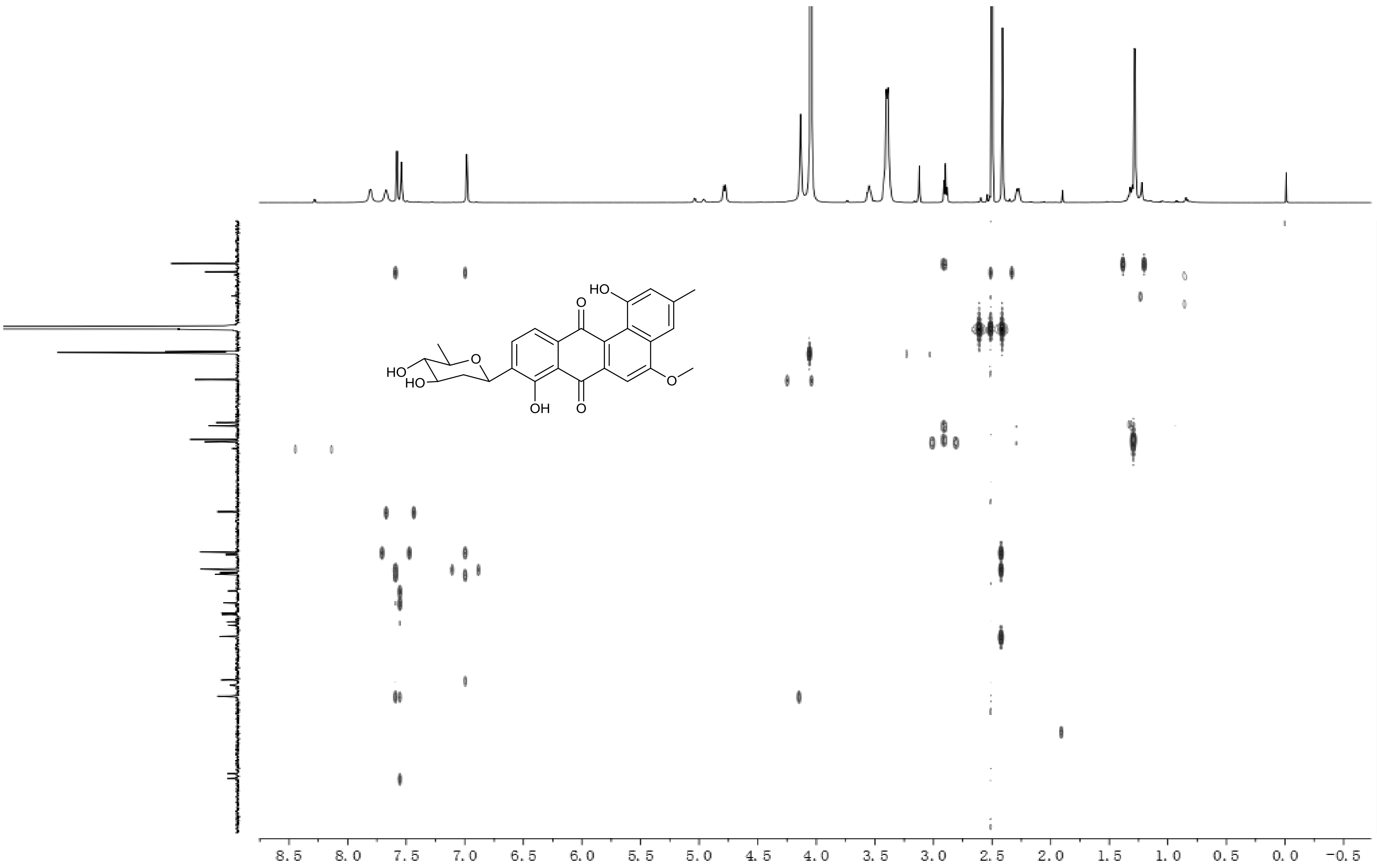

Figure S21. NOESY spectrum of compound **3** in DMSO-d_6/CD_3OD.

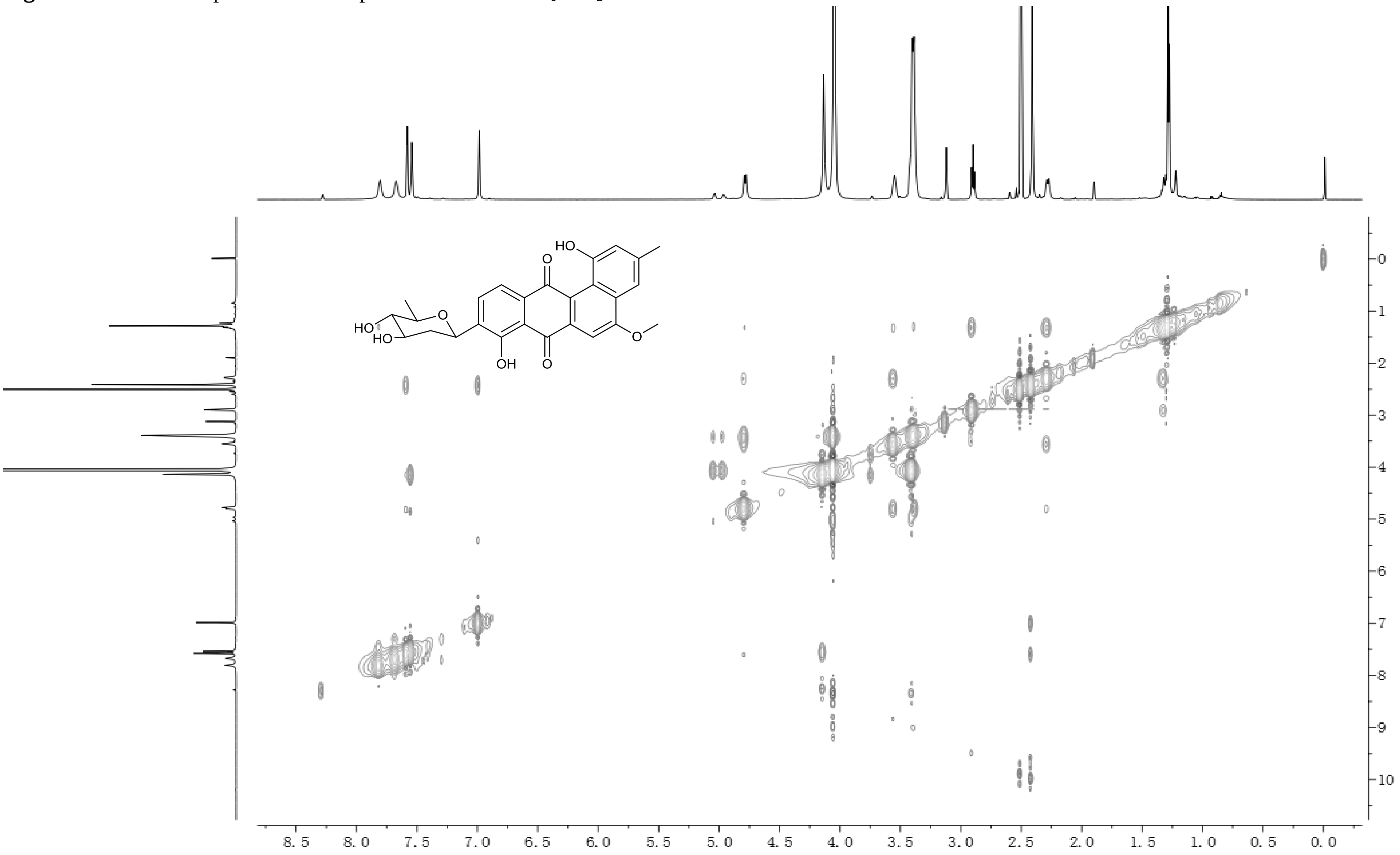

Figure S22. ^{1}H NMR (700 MHz) spectrum of compound **4** in DMSO-d_6/CD_3OD.

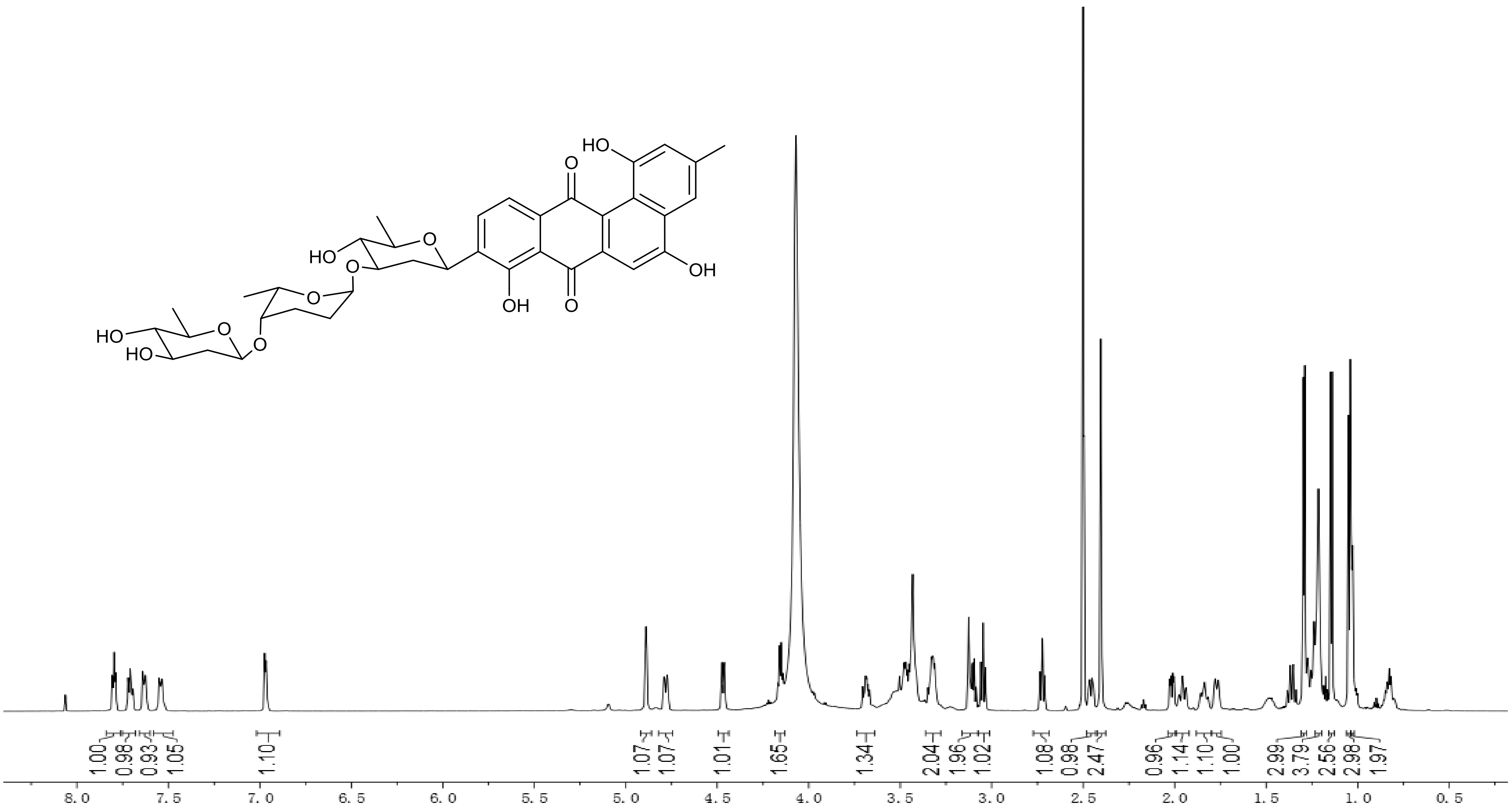

Figure S23. ^{13}C NMR (176 MHz) spectrum of compound **4** in DMSO-d_6/CD_3OD.

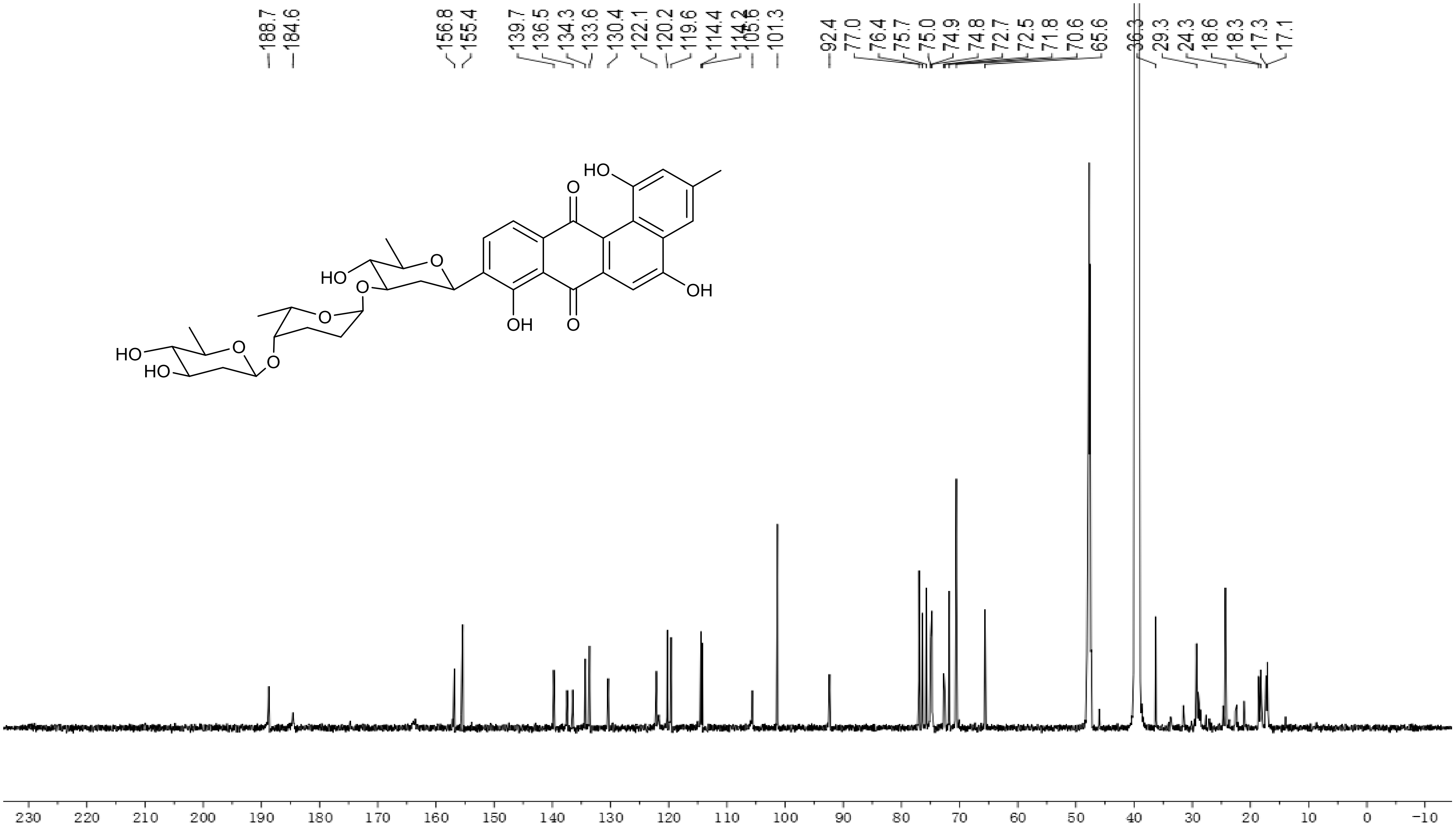

Figure S24. ^{13}C DEPT spectrum of compound **4** in DMSO-d_6/CD_3OD.

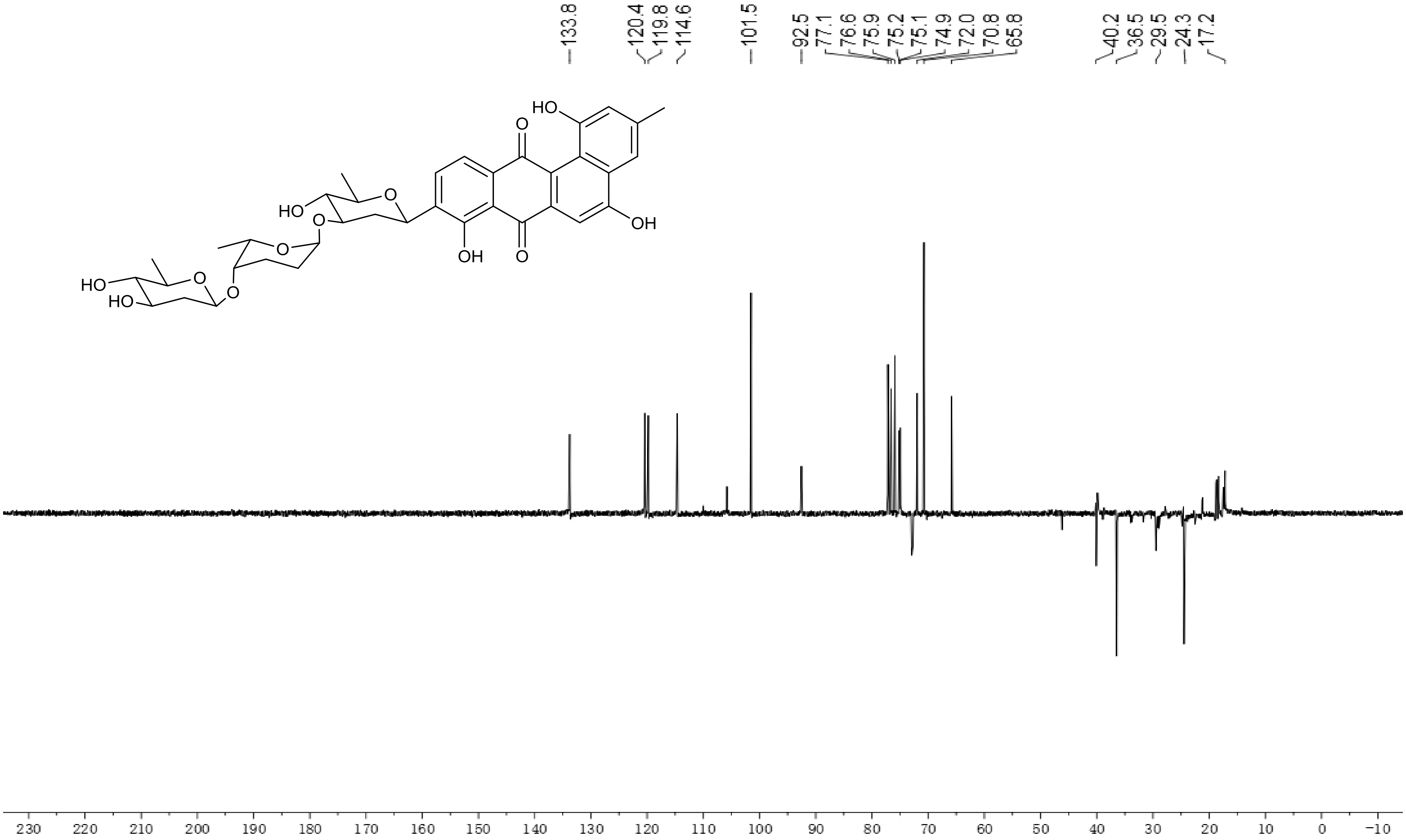

Figure S25. ^{1}H-^{1}H COSY spectrum of compound **4** in DMSO-d_6/CD_3OD.

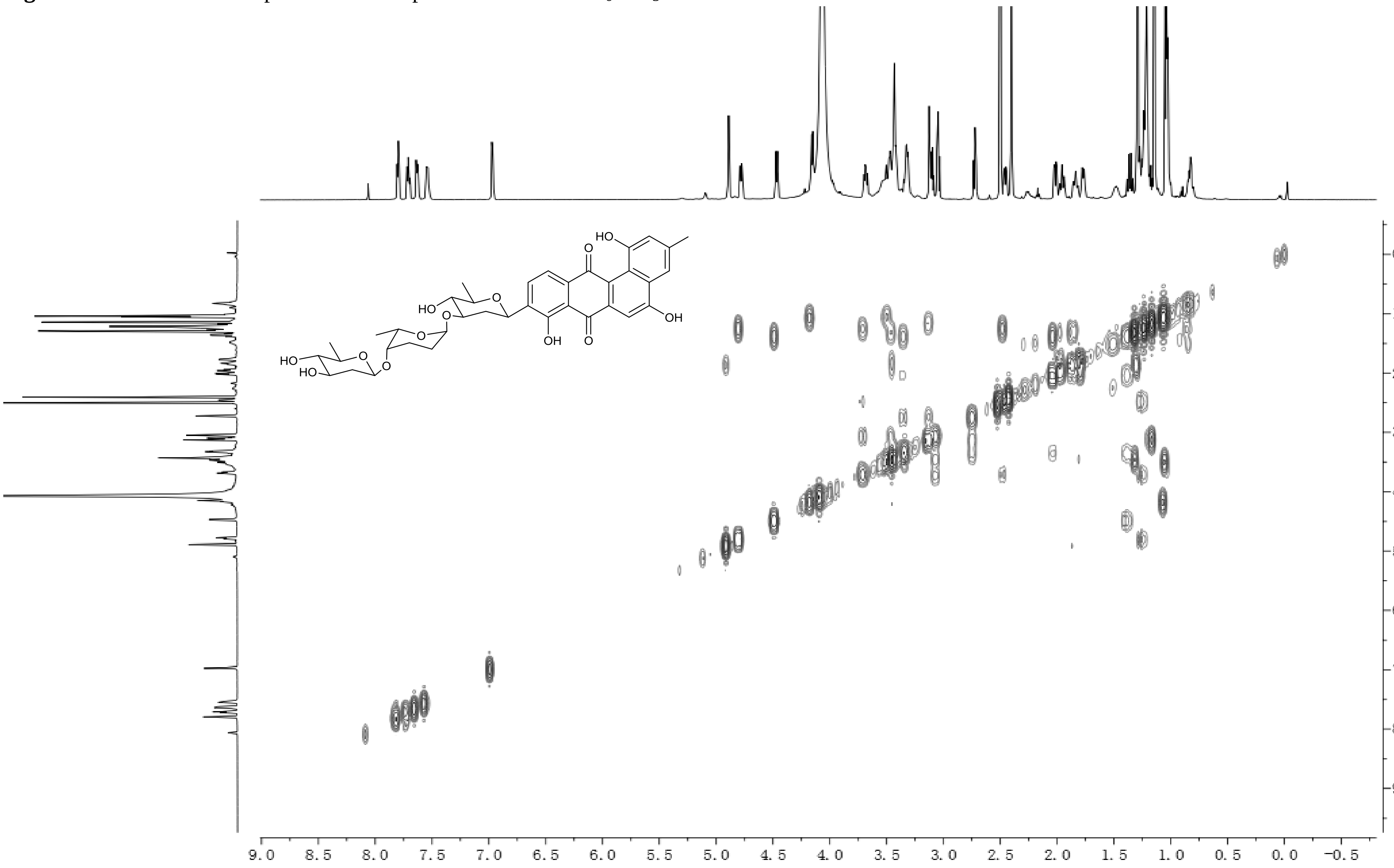

Figure S26. HSQC spectrum of compound **4** in DMSO-d_6/CD_3OD.

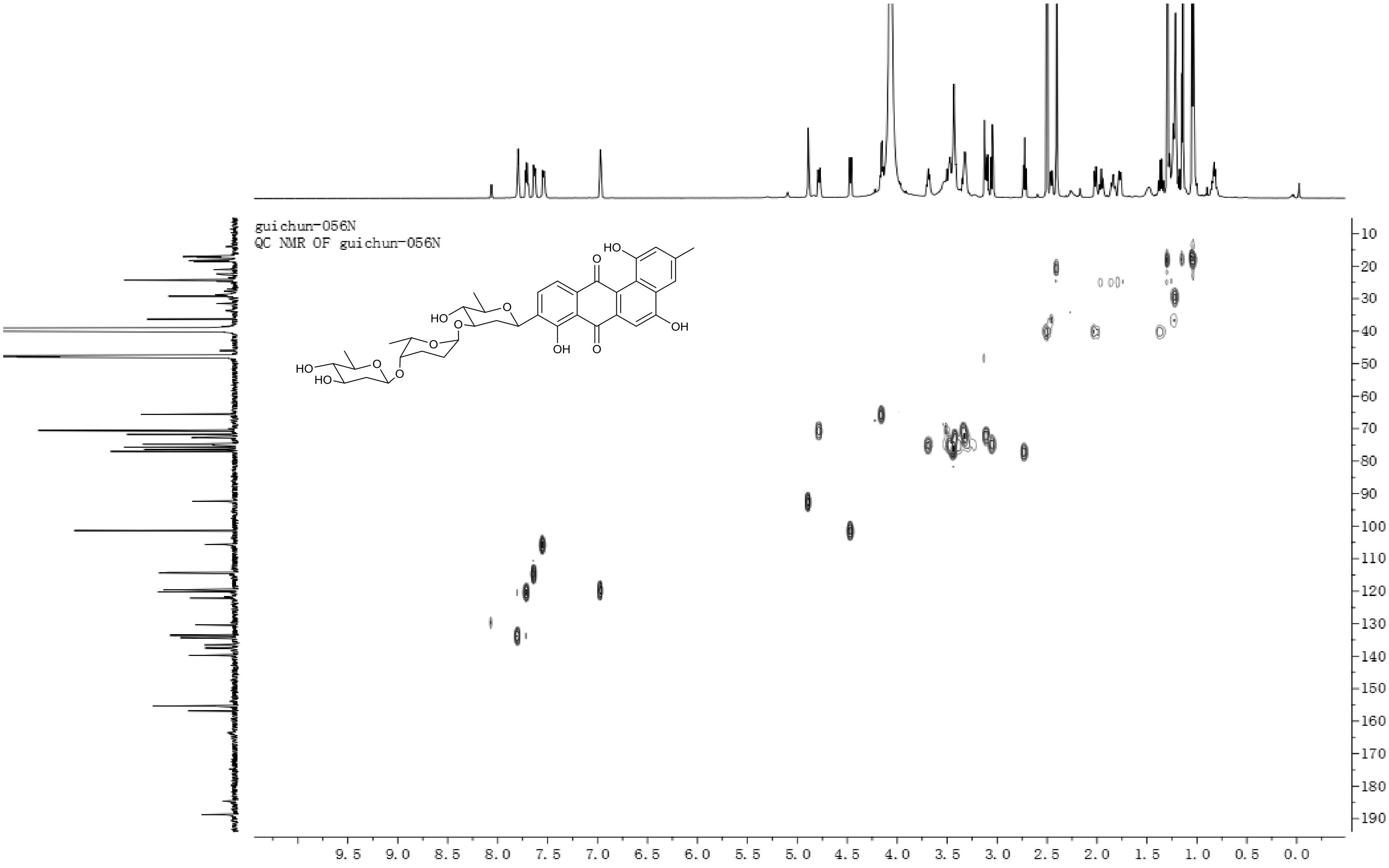

Figure S27. HMBC spectrum of compound **4** in DMSO-d_6/CD_3OD.

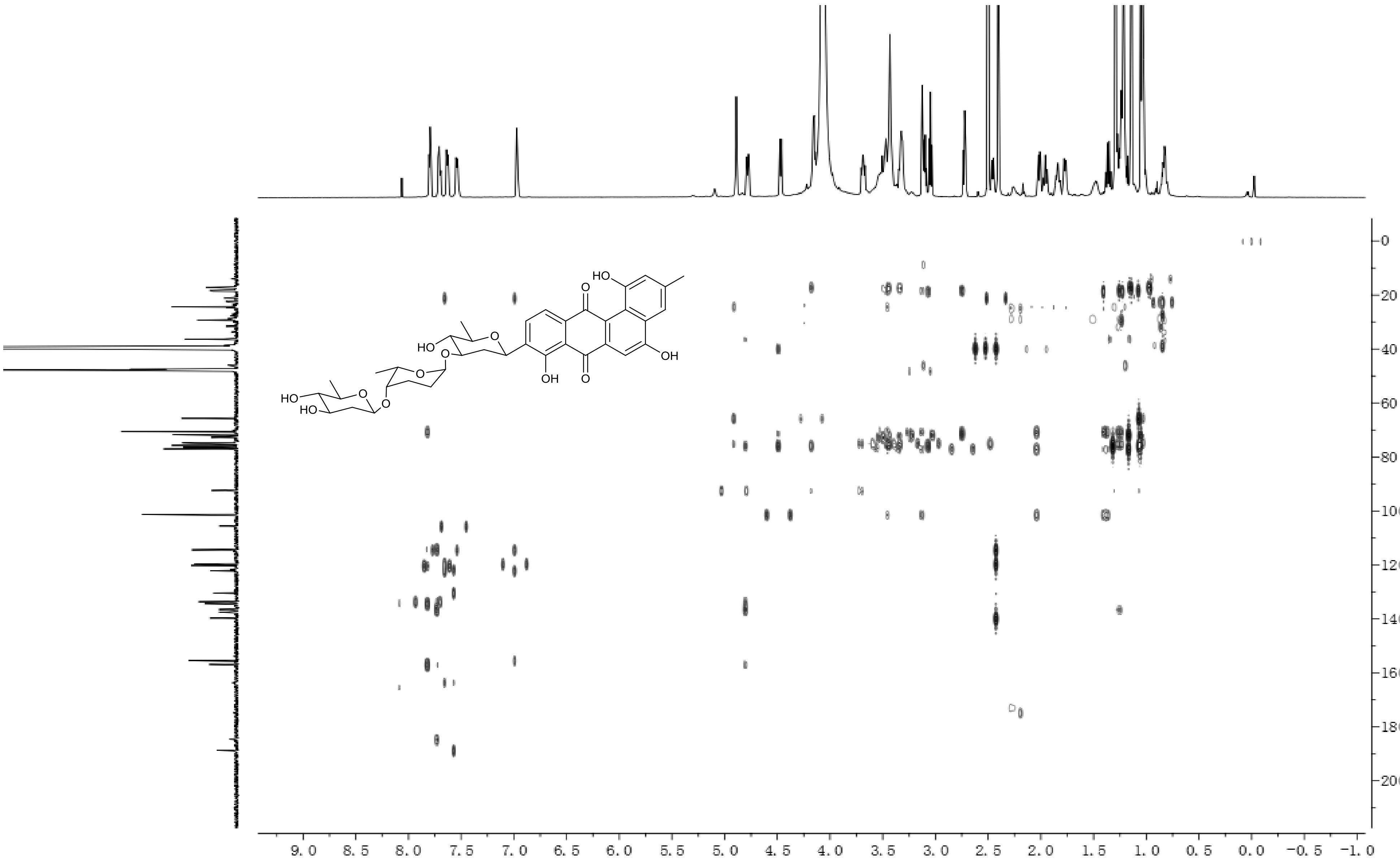

Figure S28. NOESY spectrum of compound **4** in DMSO-d_6/CD_3OD.

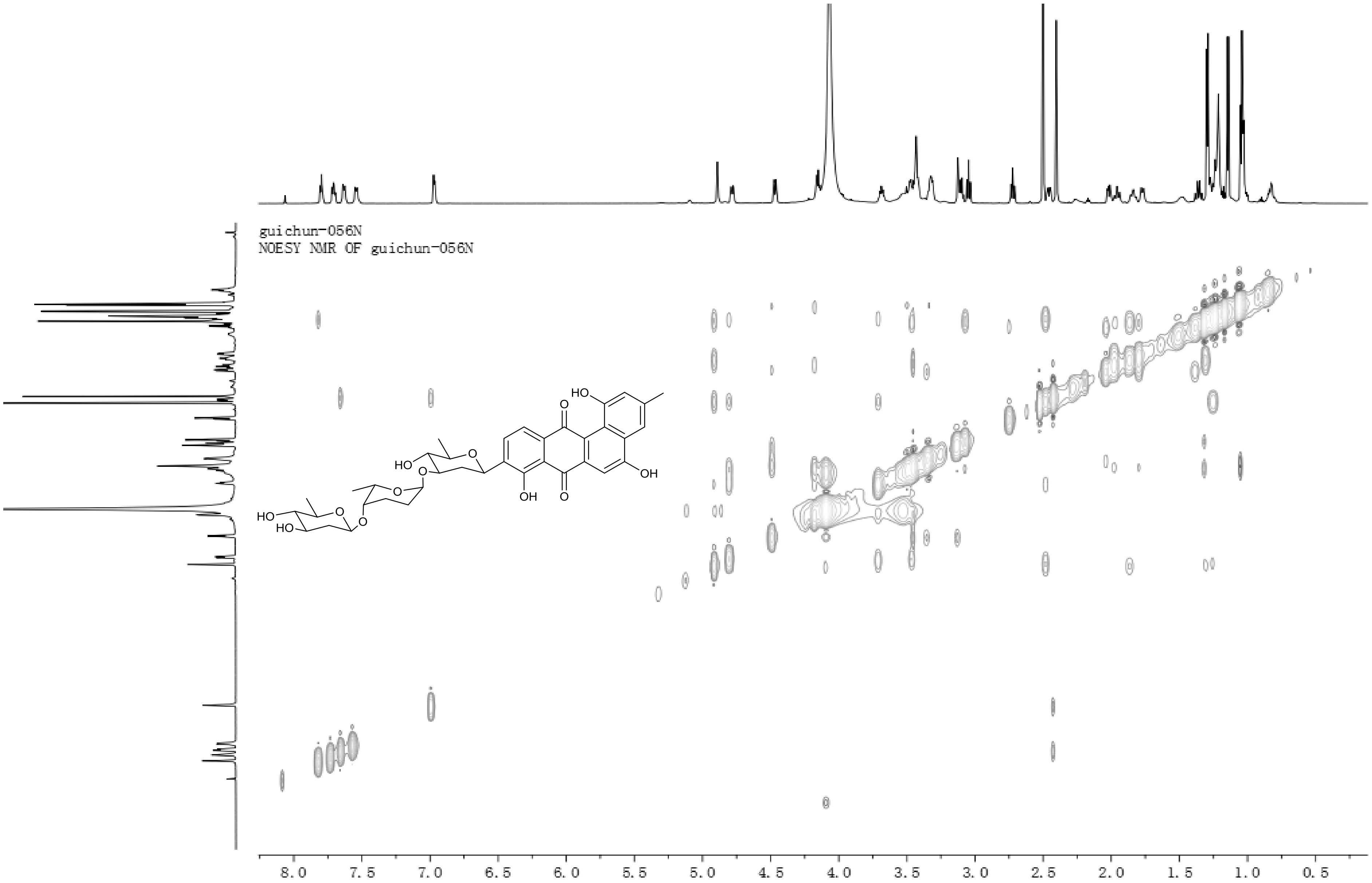

Figure S29. ^{1}H NMR (700 MHz) spectrum of compound **5** in DMSO-d_6/CD_3OD.

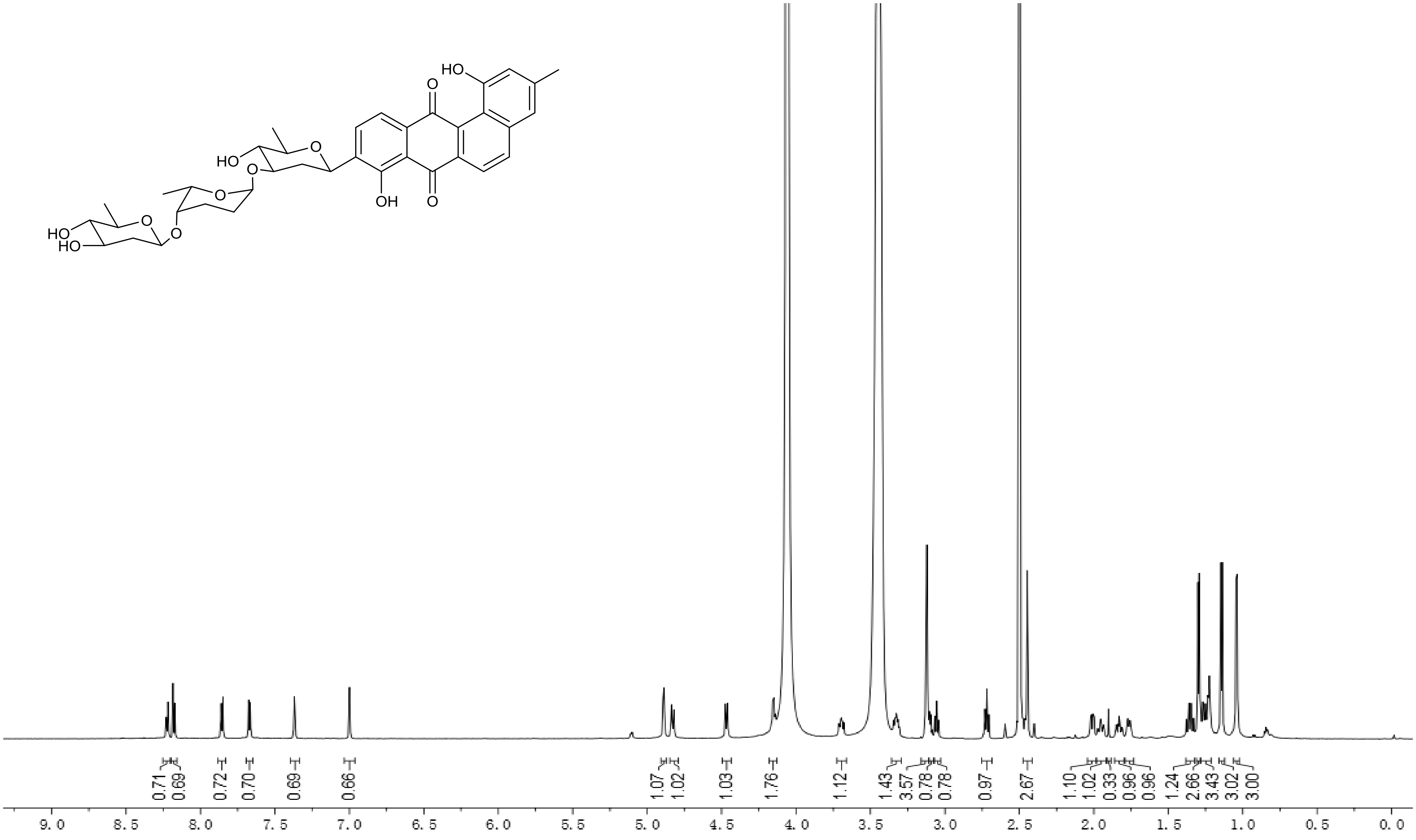

Figure S30. ^{13}C NMR (176 MHz) spectrum of compound **5** in DMSO-d_6/CD_3OD.

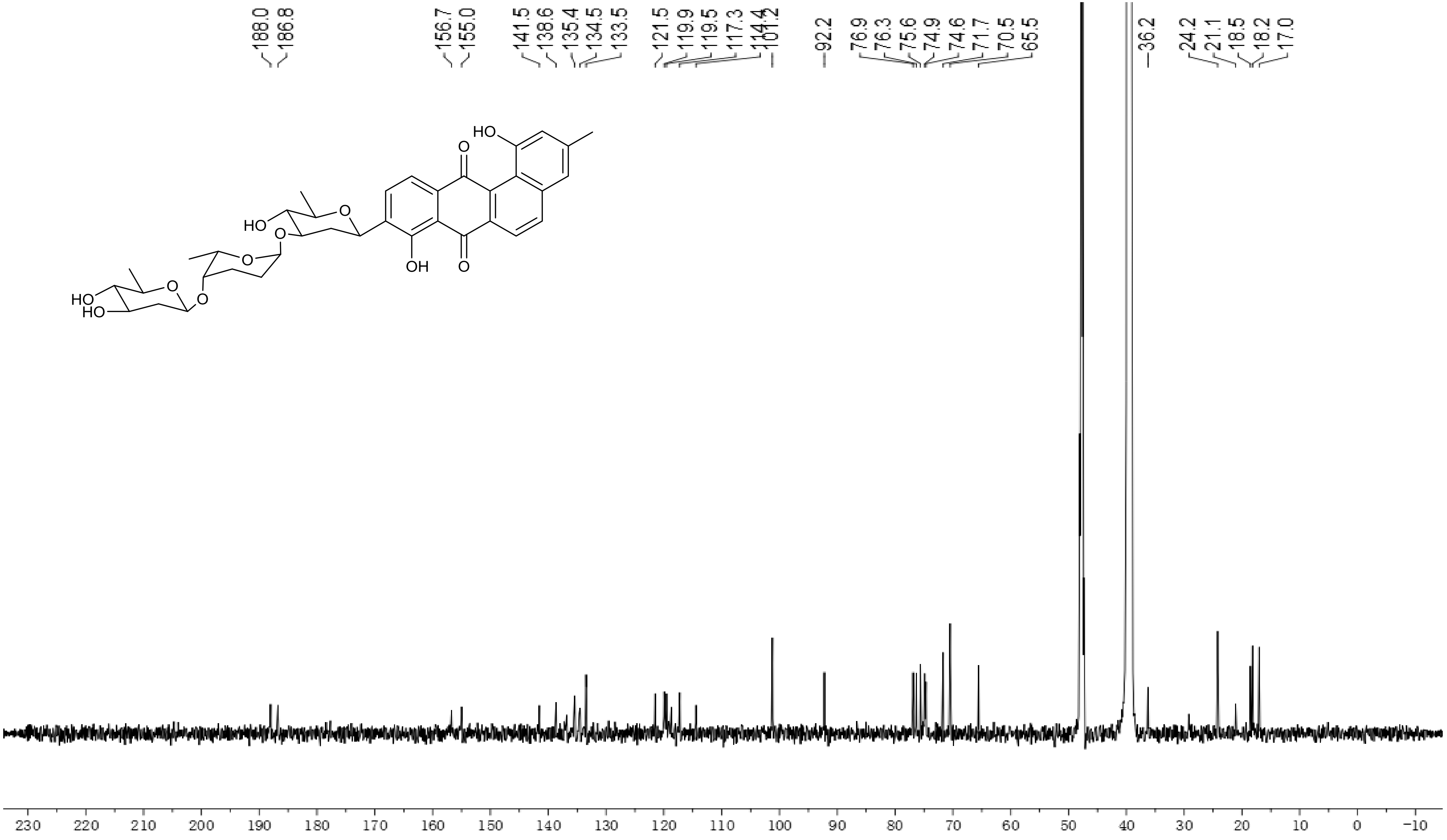

Figure S31. ^{13}C DEPT spectrum of compound **5** in DMSO-d_6/CD_3OD.

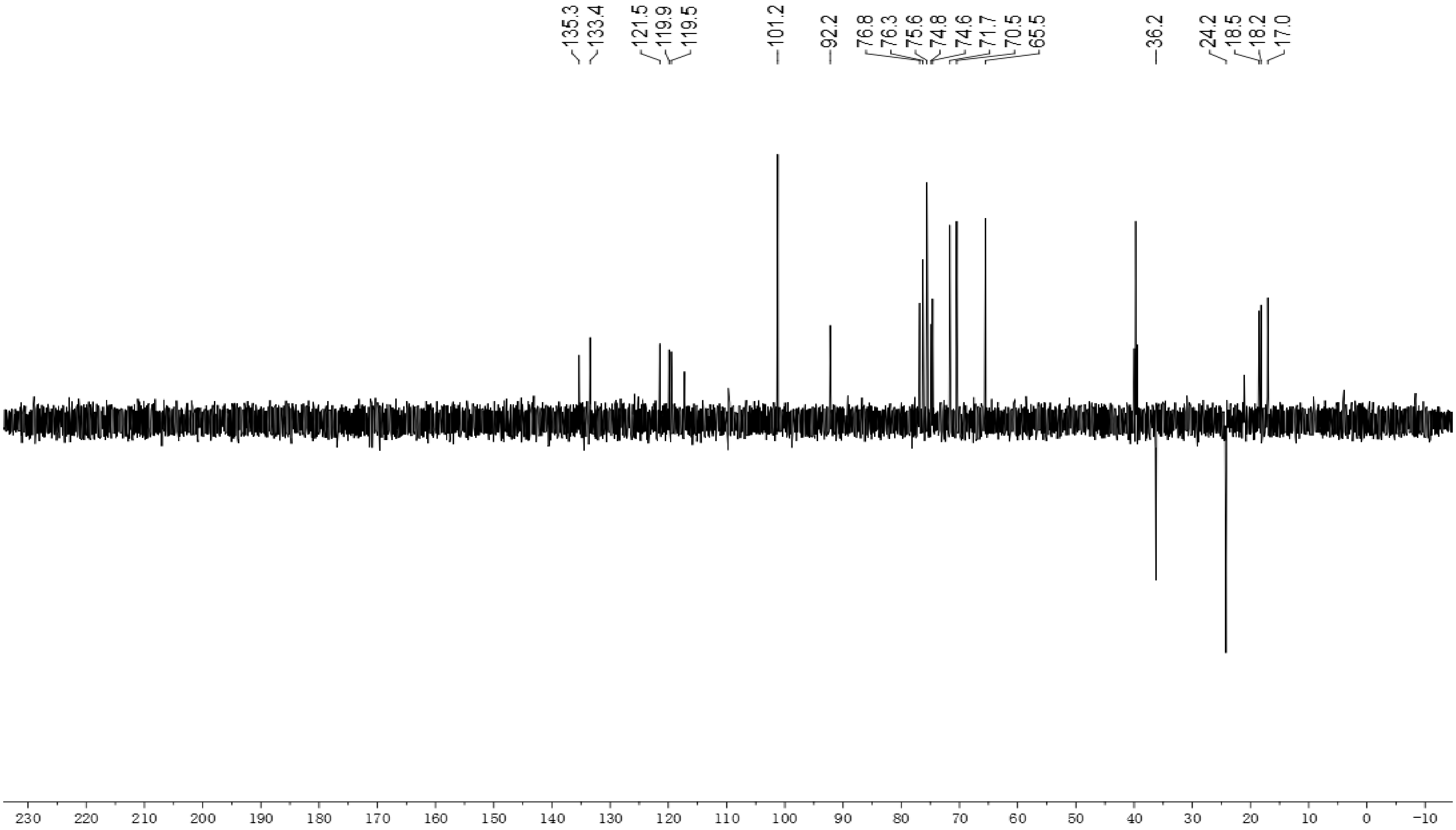

Figure S32. ^{1}H-^{1}H COSY spectrum of compound **5** in DMSO-d_6/CD_3OD.

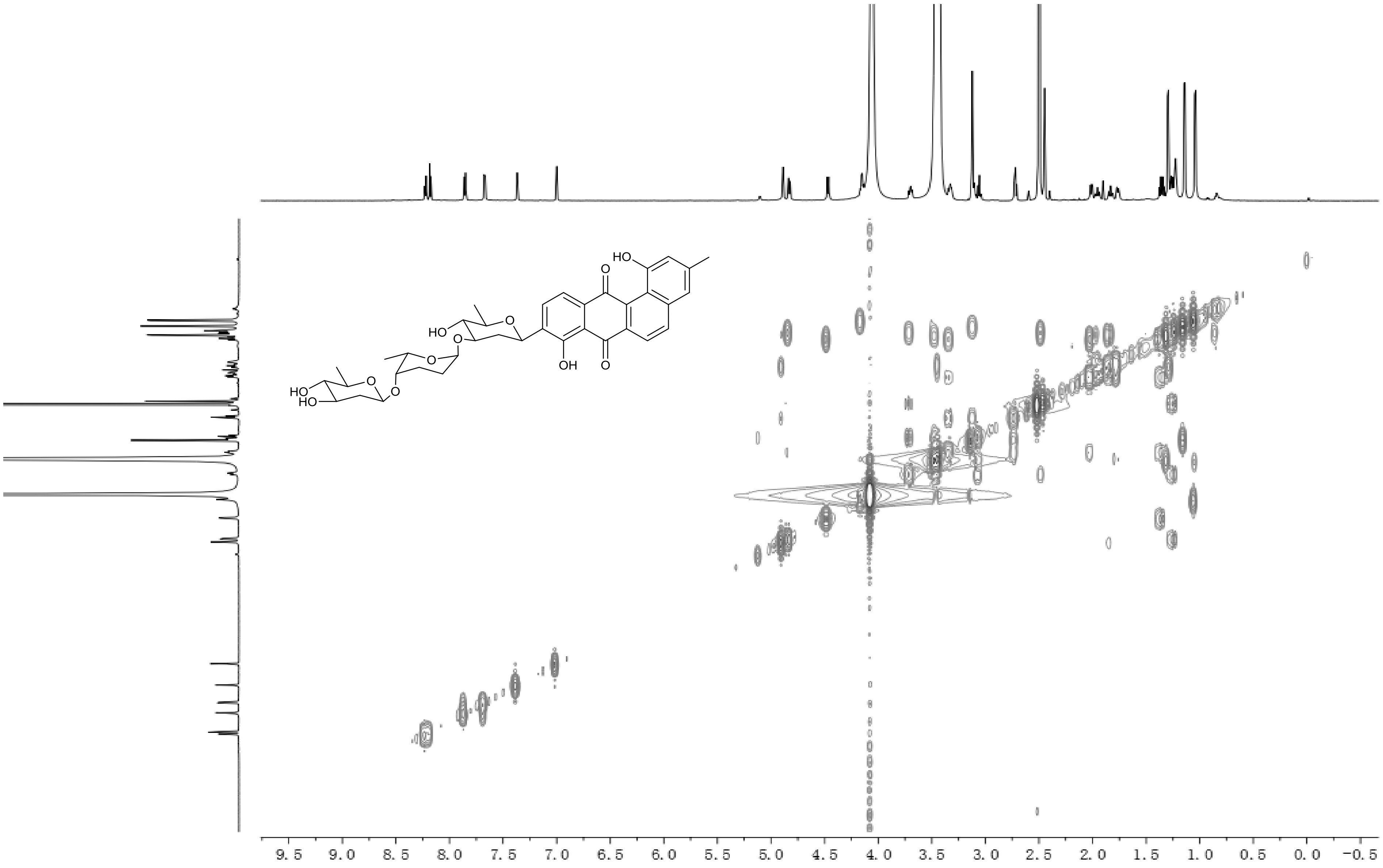

Figure S33. HSQC spectrum of compound **5** in DMSO-d_6/CD_3OD.

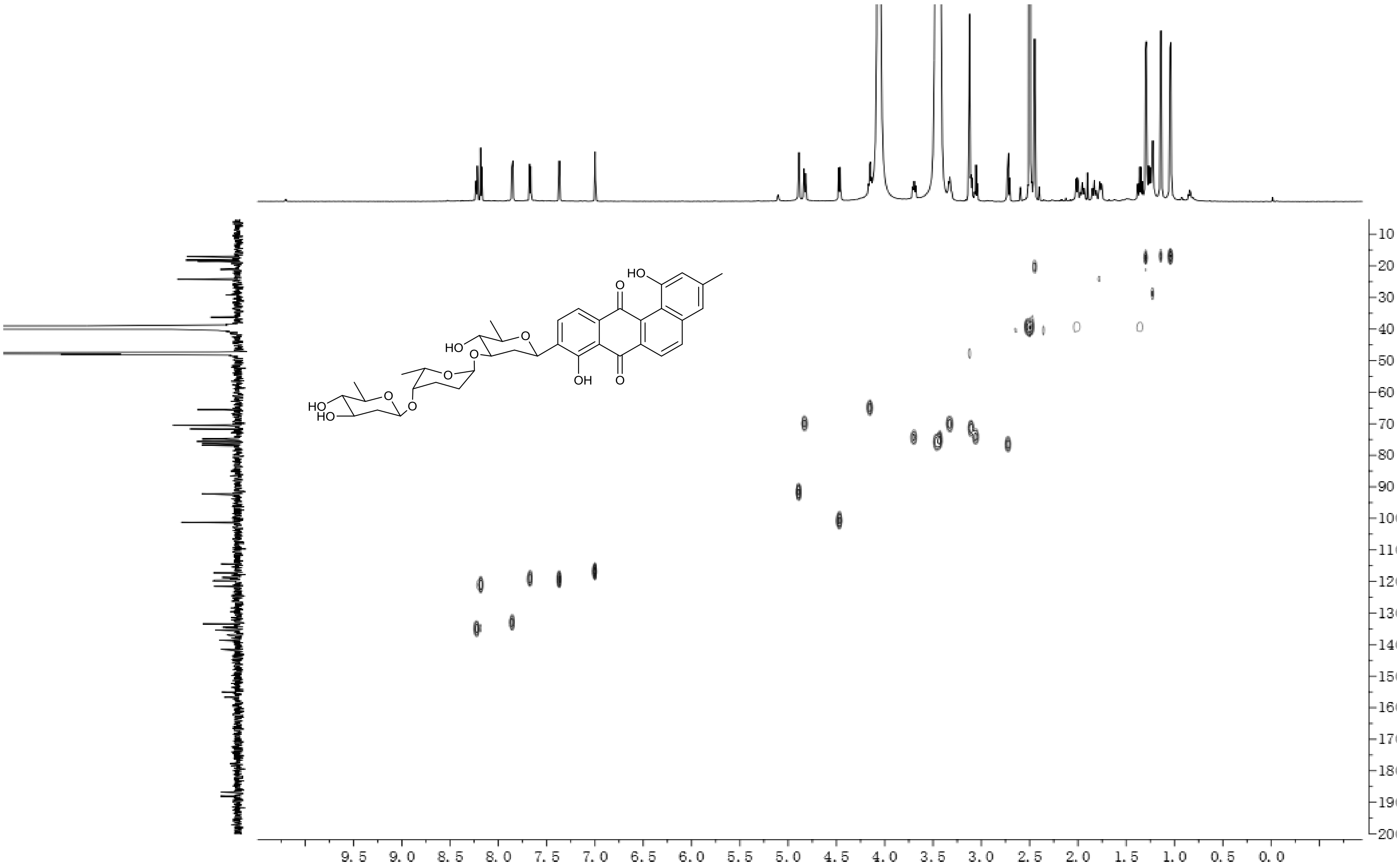

Figure S34. HMBC spectrum of compound **5** in DMSO-d_6/CD_3OD.

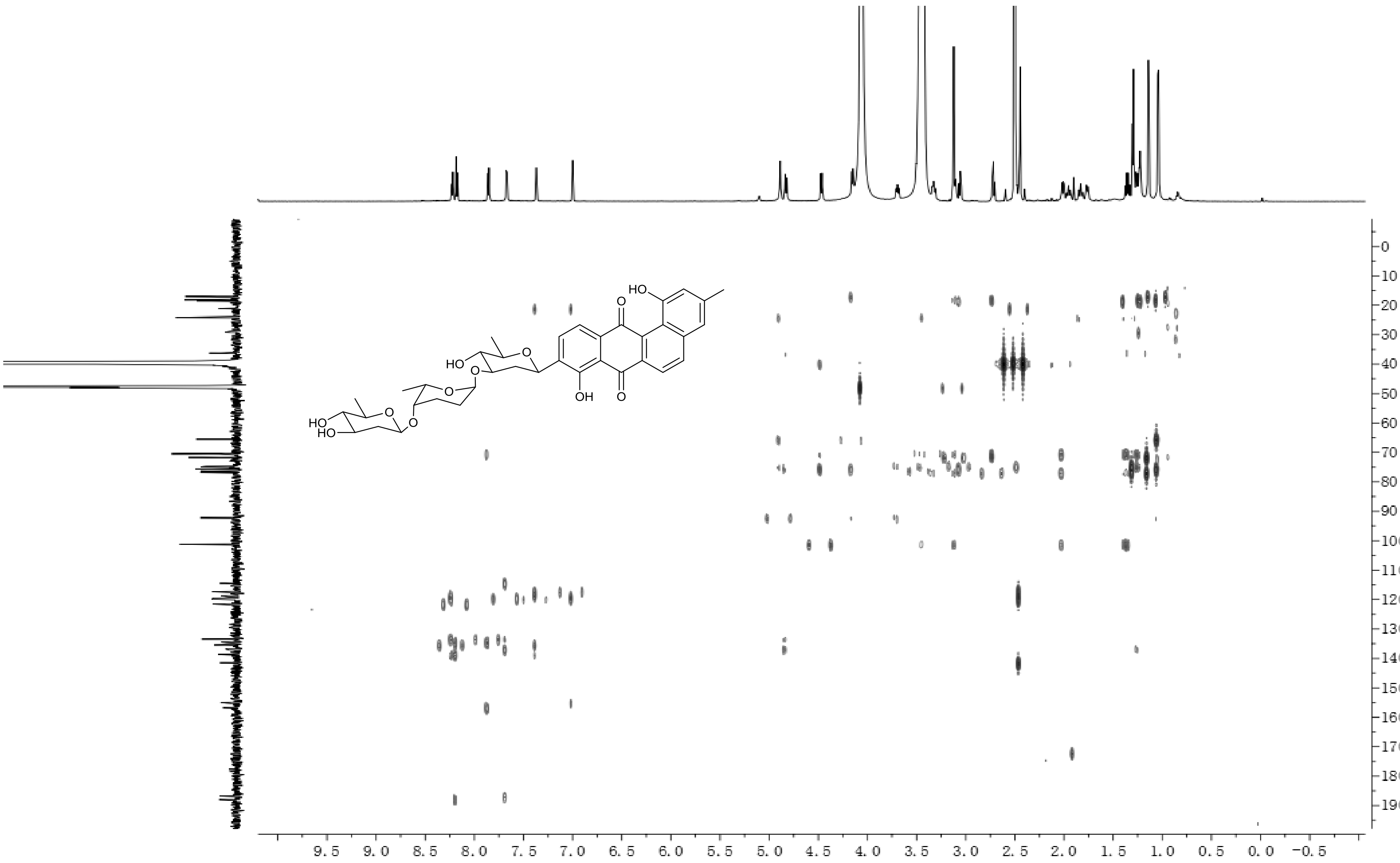

Figure S35. NOESY spectrum of compound **5** in DMSO-d_6/CD_3OD.

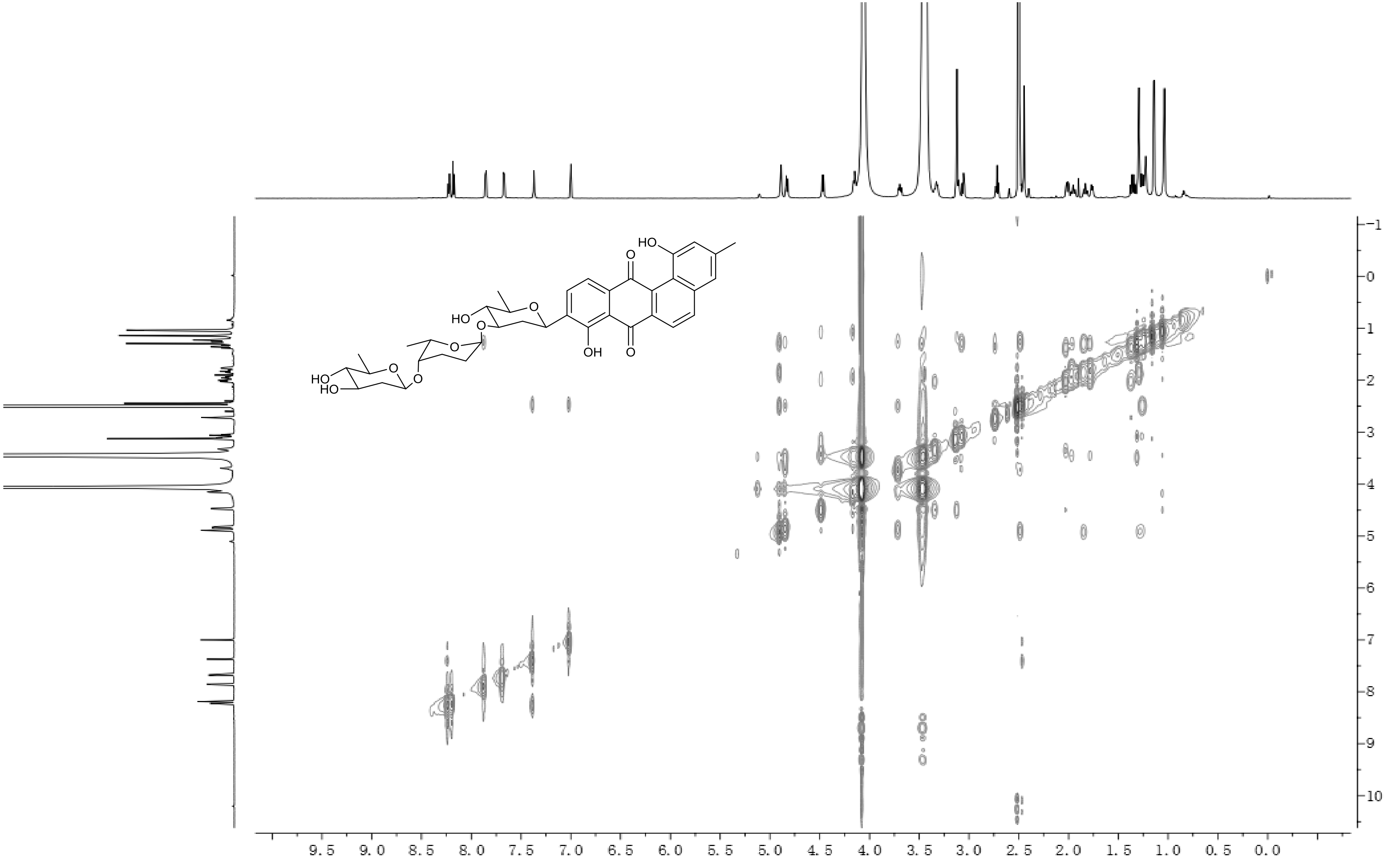

Figure S36.^{1}H NMR (700 MHz) spectrum of compound **6** in $CDCl_3$.

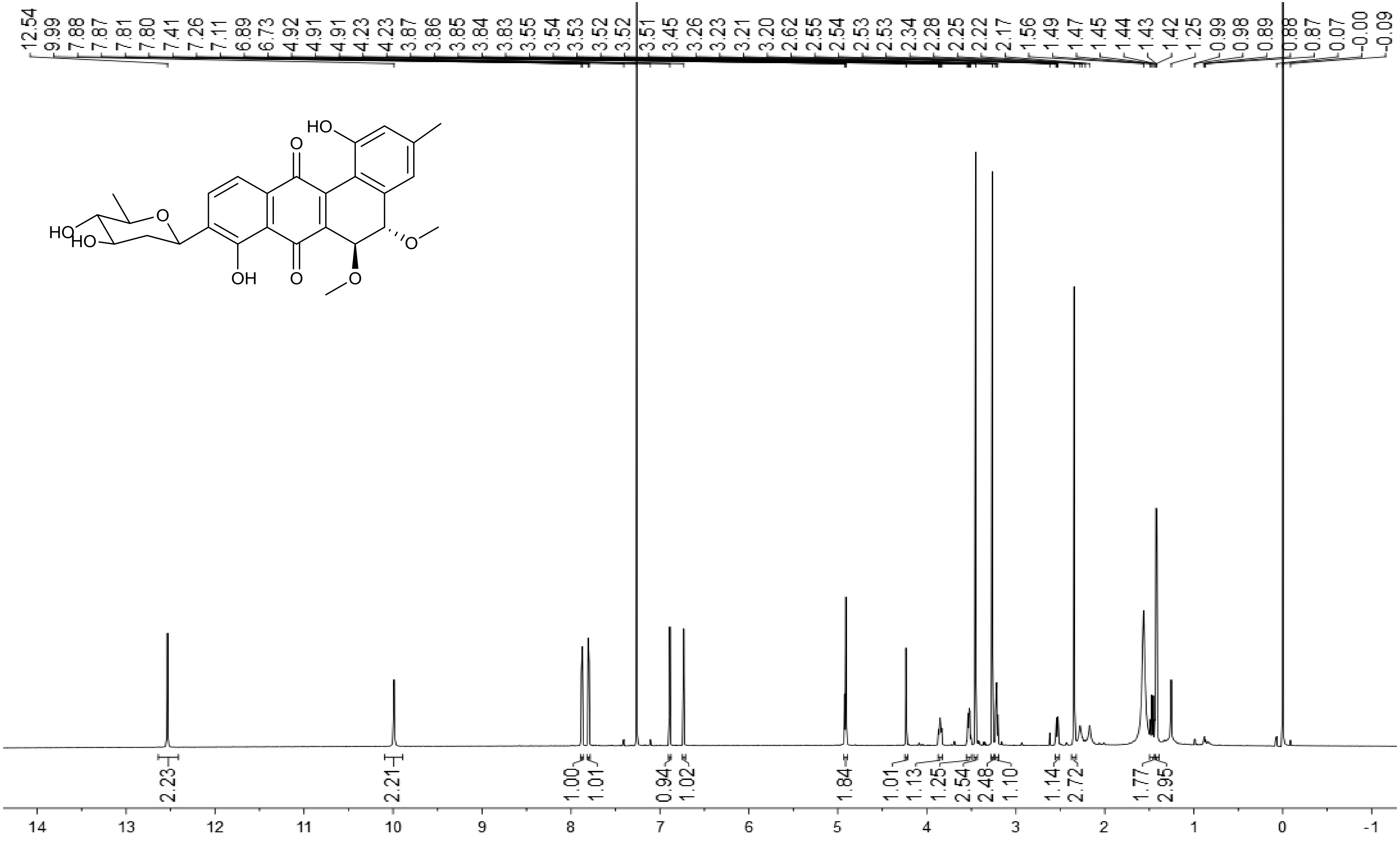

Figure S37. ^{13}C NMR (176 MHz) spectrum of compound **6** in $CDCl_3$.

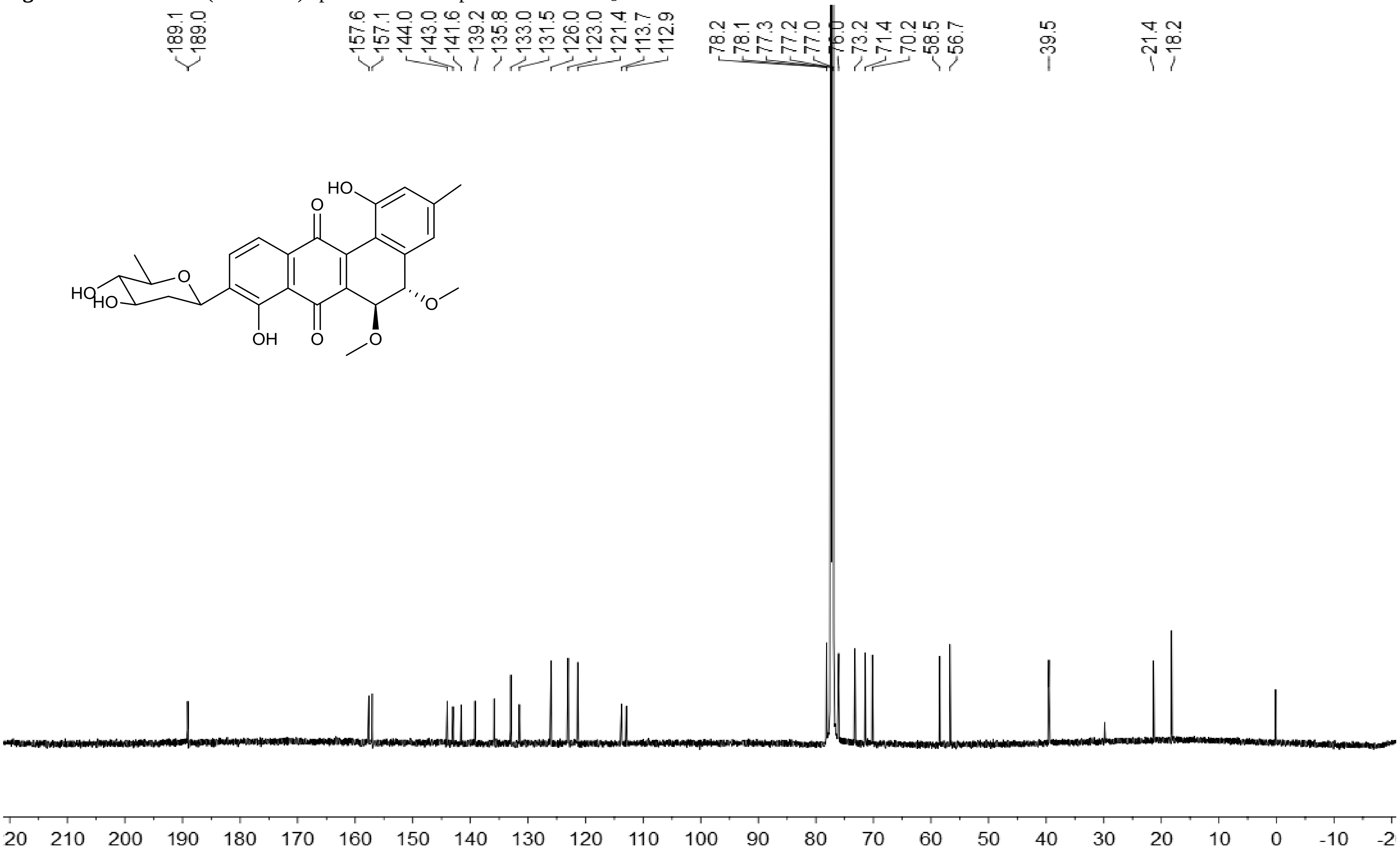

Figure S38. ^{13}C DEPT spectrum of compound **6** in $CDCl_3$.

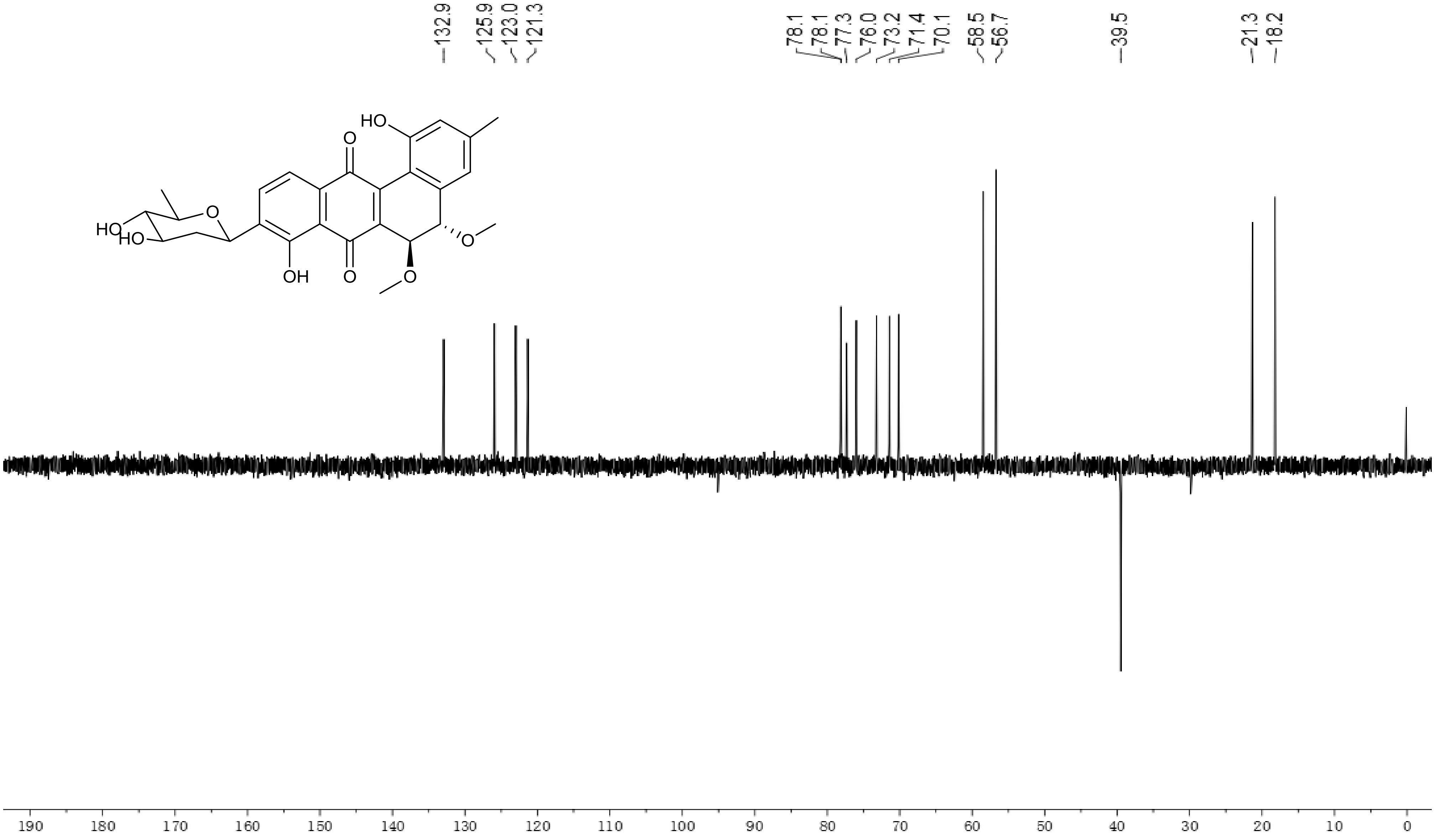

Figure S39. ^1H-^1H COSY spectrum of compound **6** in $CDCl_3$.

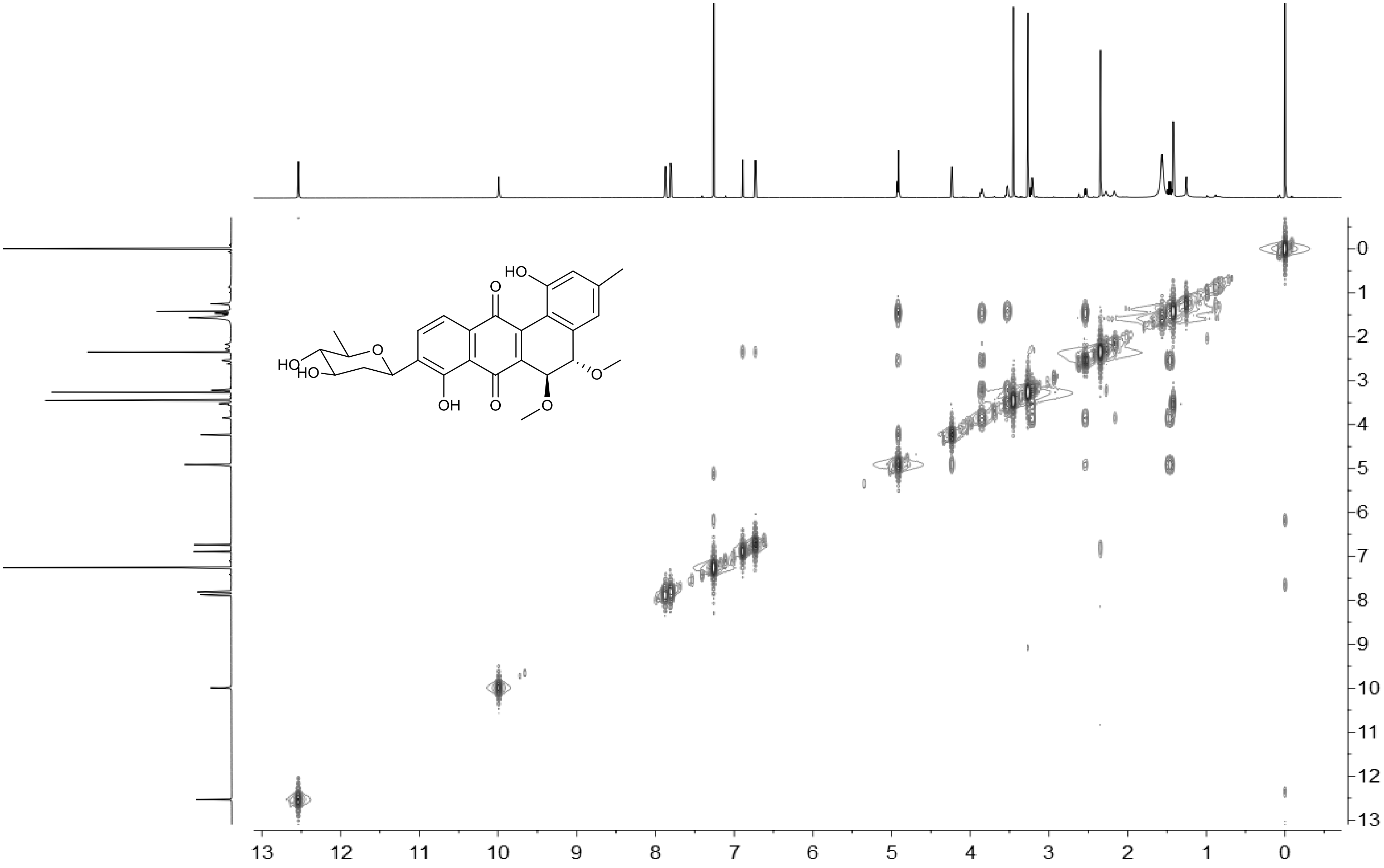

Figure S40. HSQC spectrum of compound **6** in $CDCl_3$.

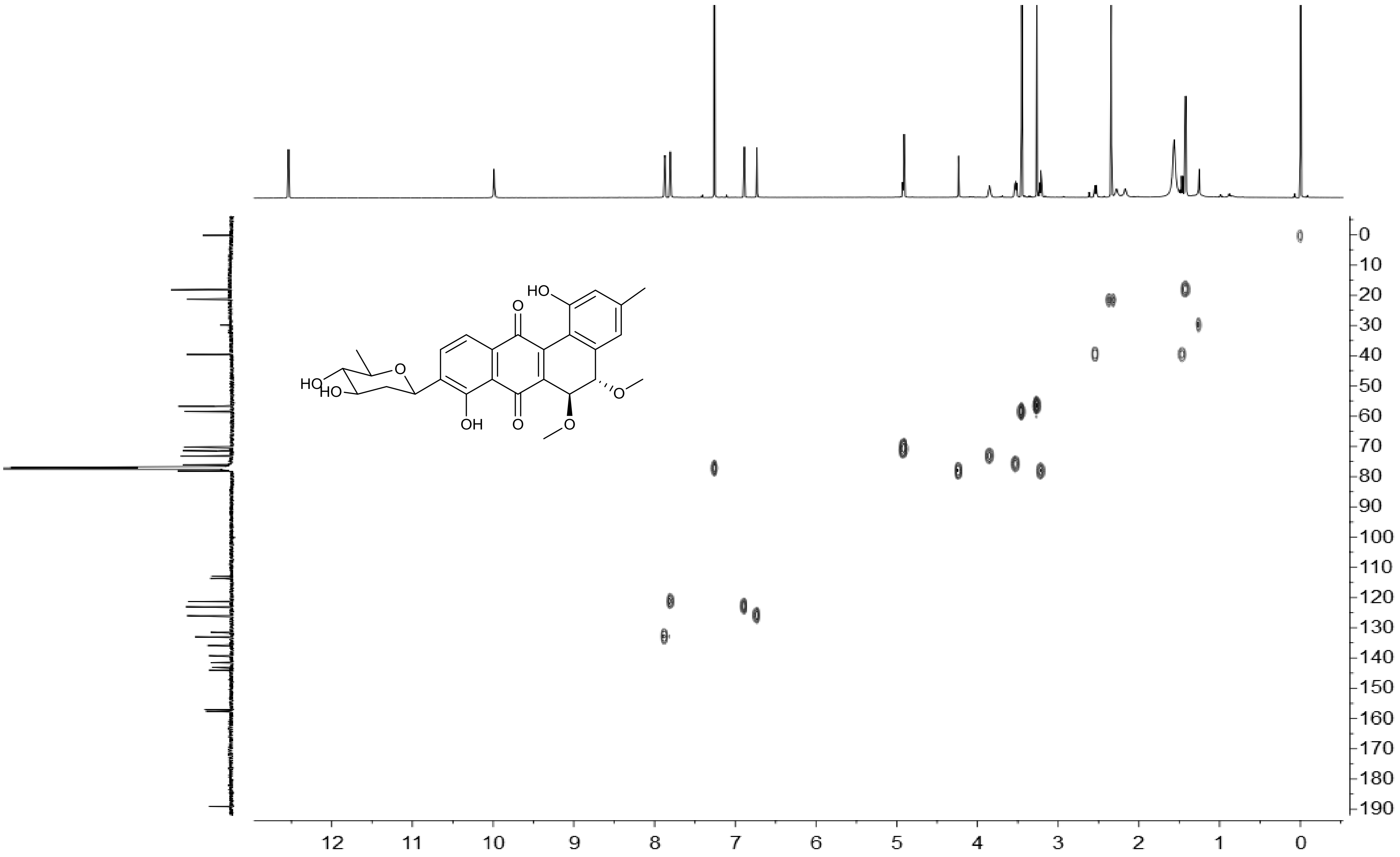

Figure S41. HMBC spectrum of compound **6** in $CDCl_3$.

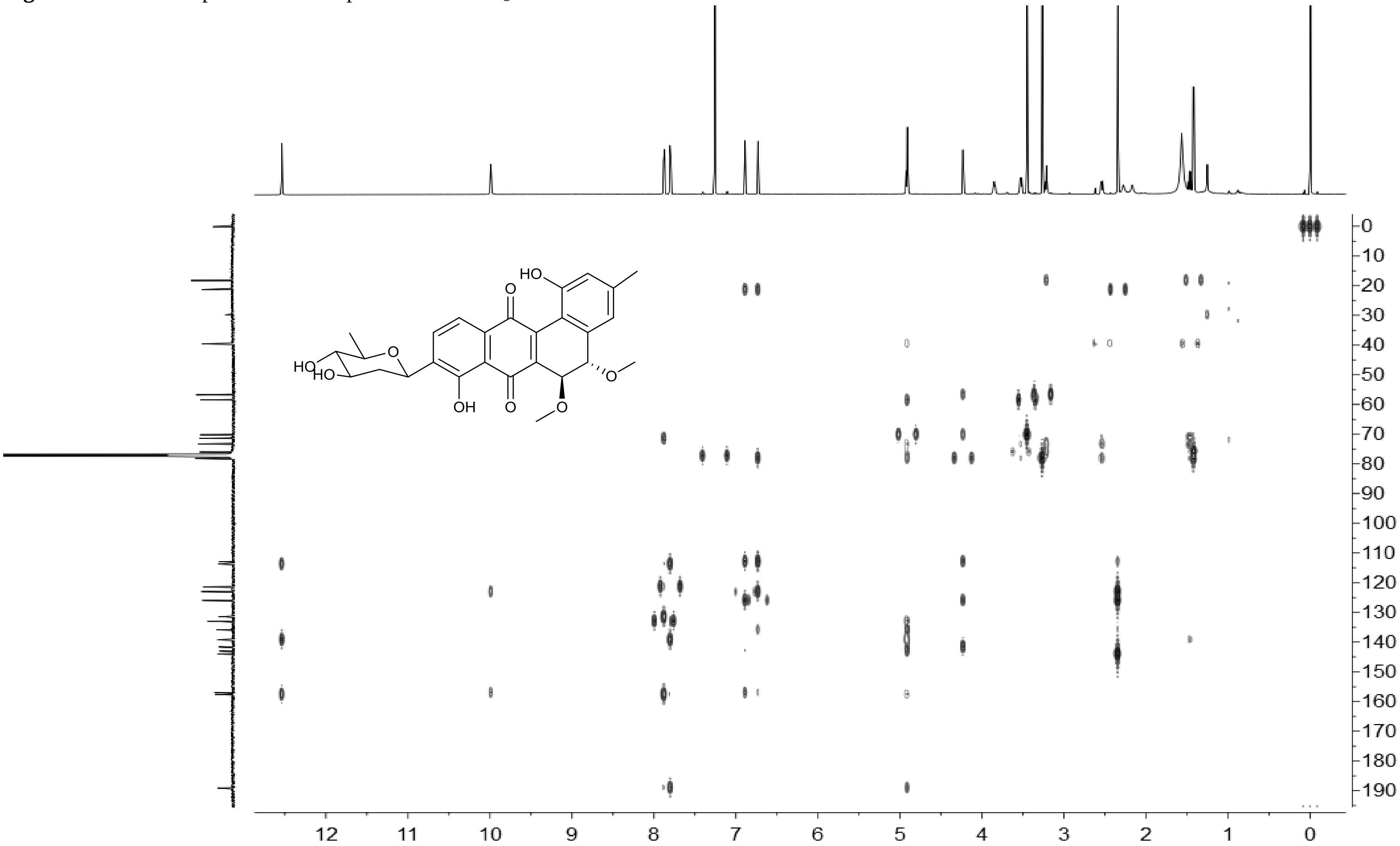

Figure S42. NOESY spectrum of compound **6** in $CDCl_3$.

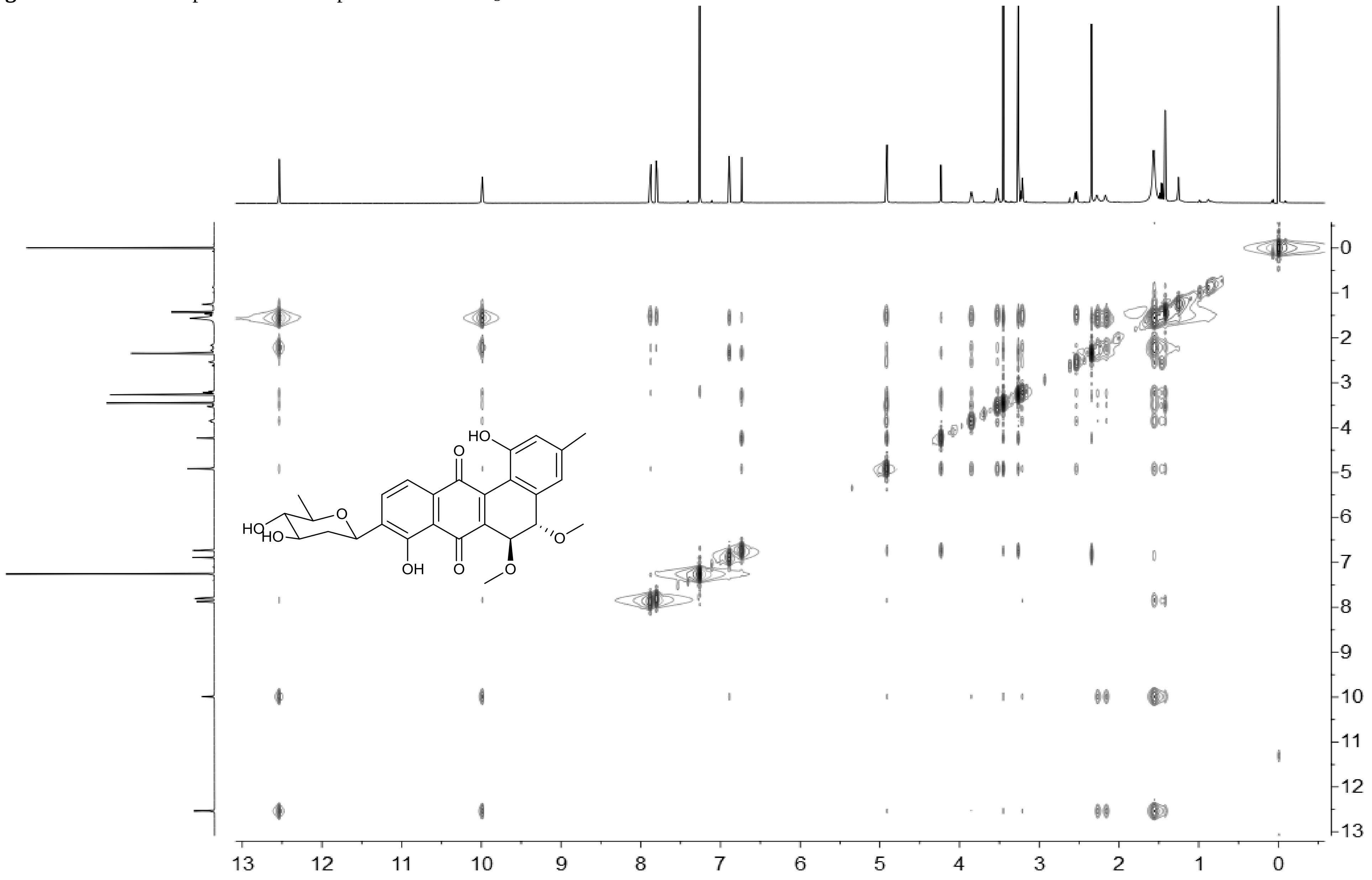

Figure S43. ^{1}H NMR (700 MHz) spectrum of **7** in $CDCl_3$.

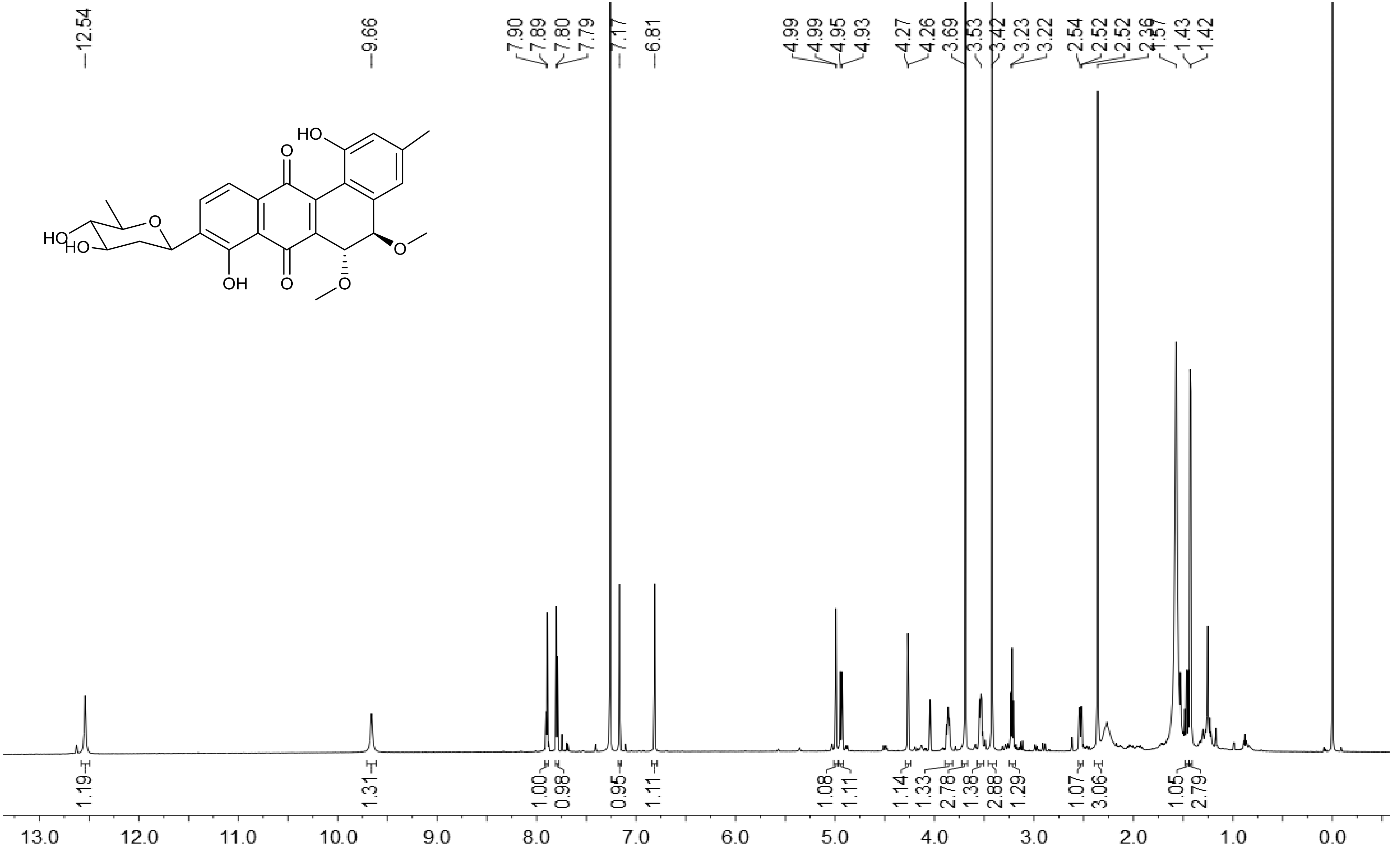

Figure S44. ^{13}C NMR (176 MHz) spectrum of compound **7** in $CDCl_3$.

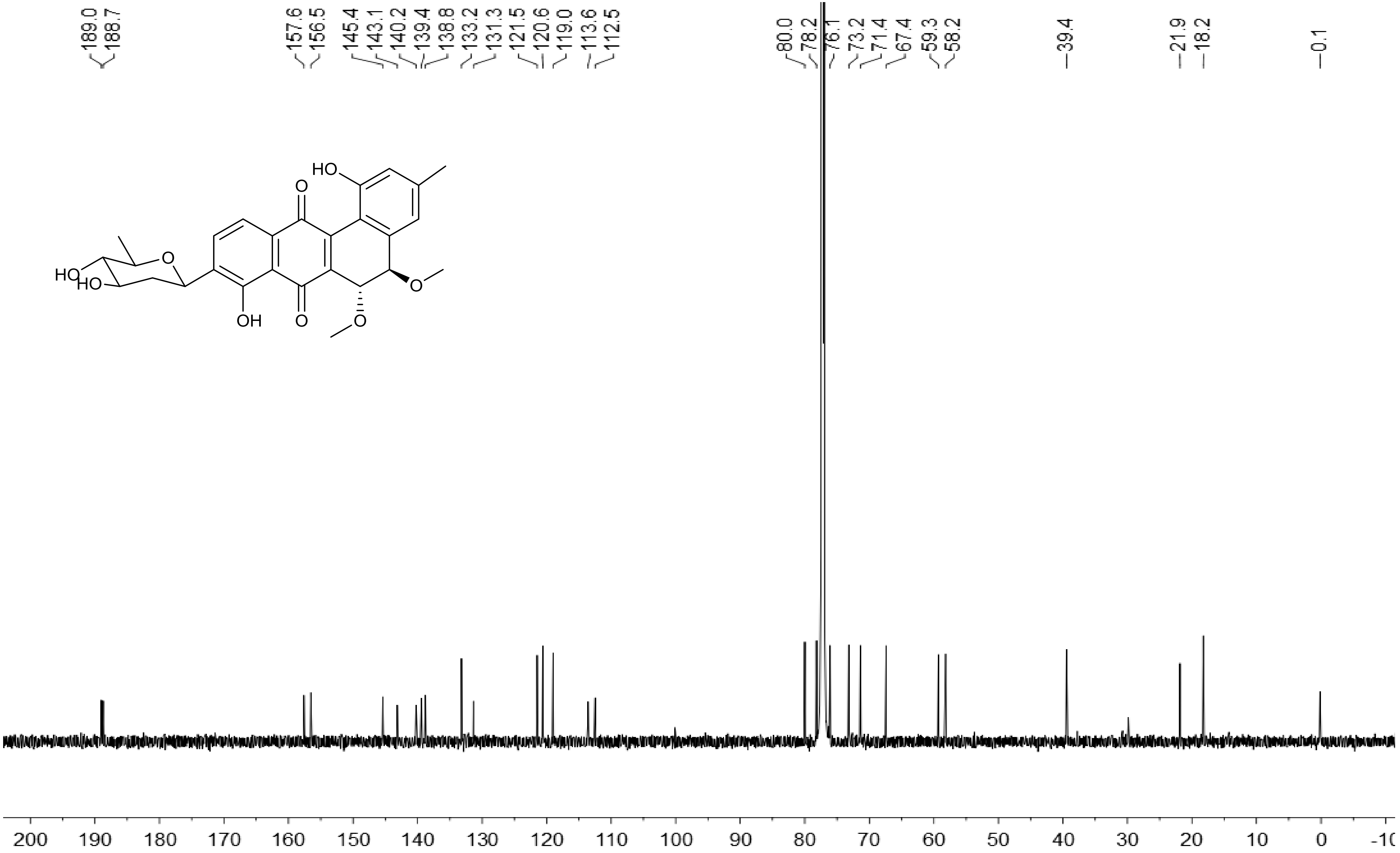

Figure S45. ^{13}C DEPT spectrum of compound **7** in $CDCl_3$.

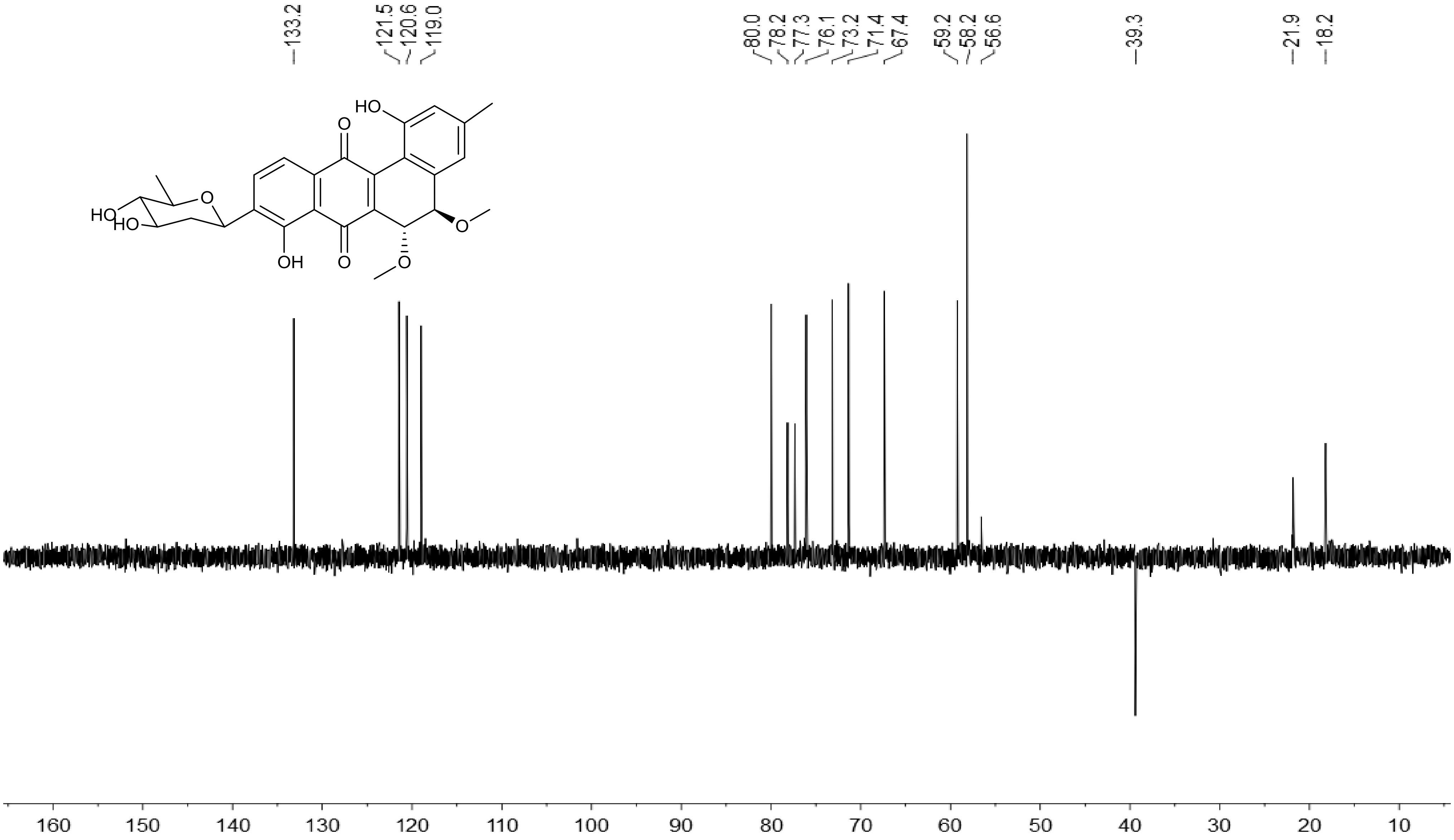

Figure S46. ^{1}H-^{1}H COSY spectrum of compound **7** in $CDCl_3$.

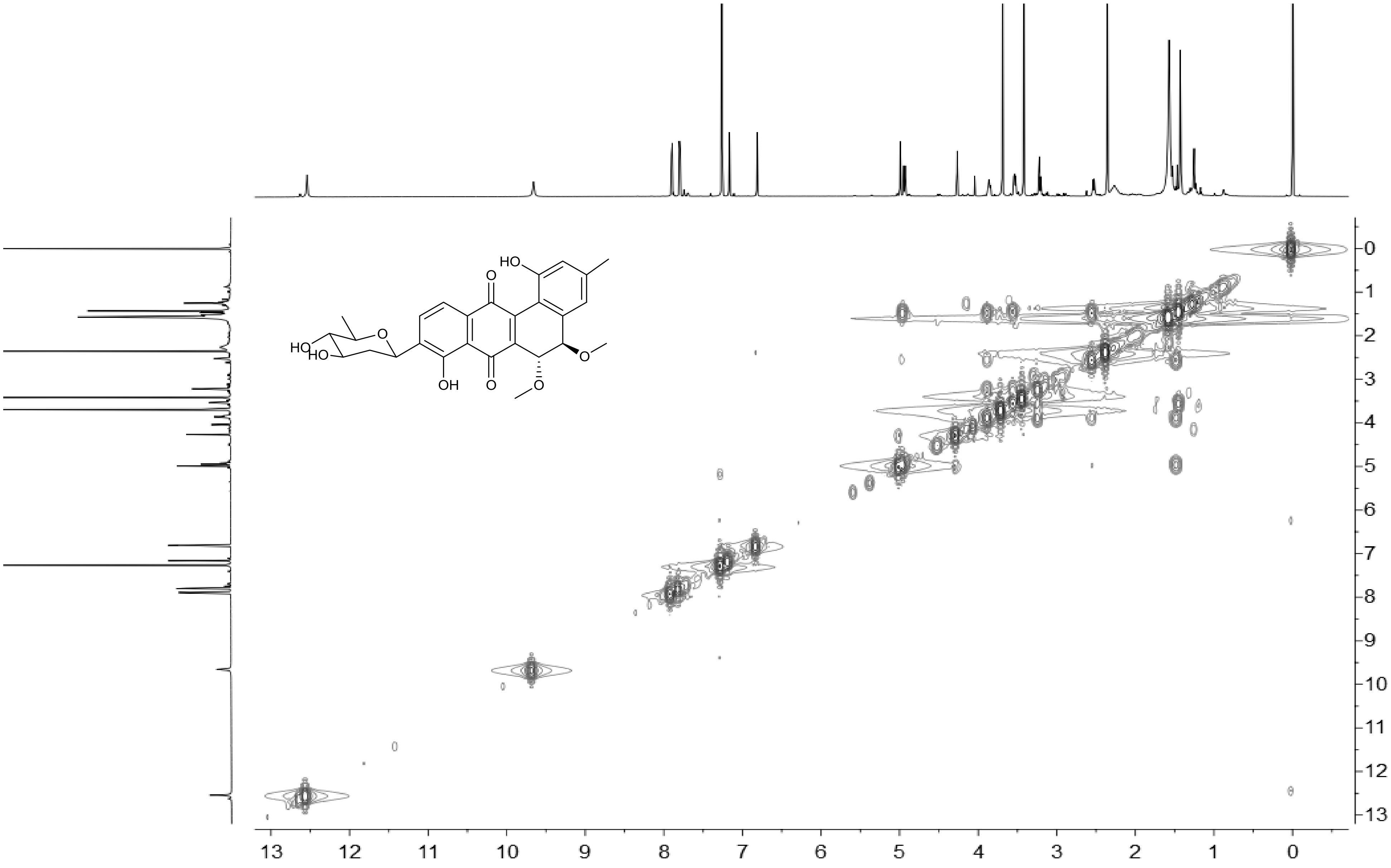

Figure S47. HSQC spectrum of compound **7** in $CDCl_3$.

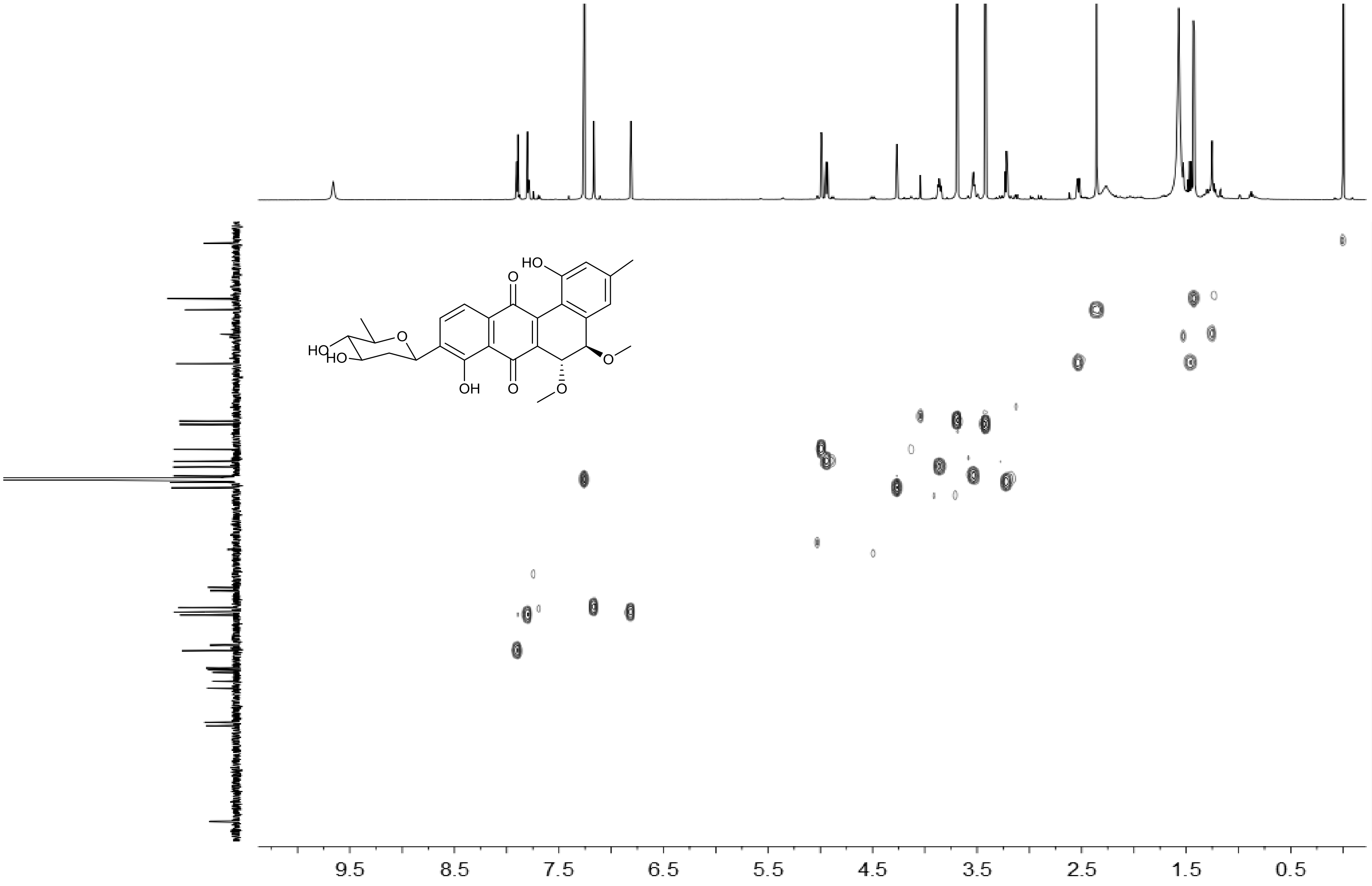

Figure S48. HMBC spectrum of compound **7** in $CDCl_3$.

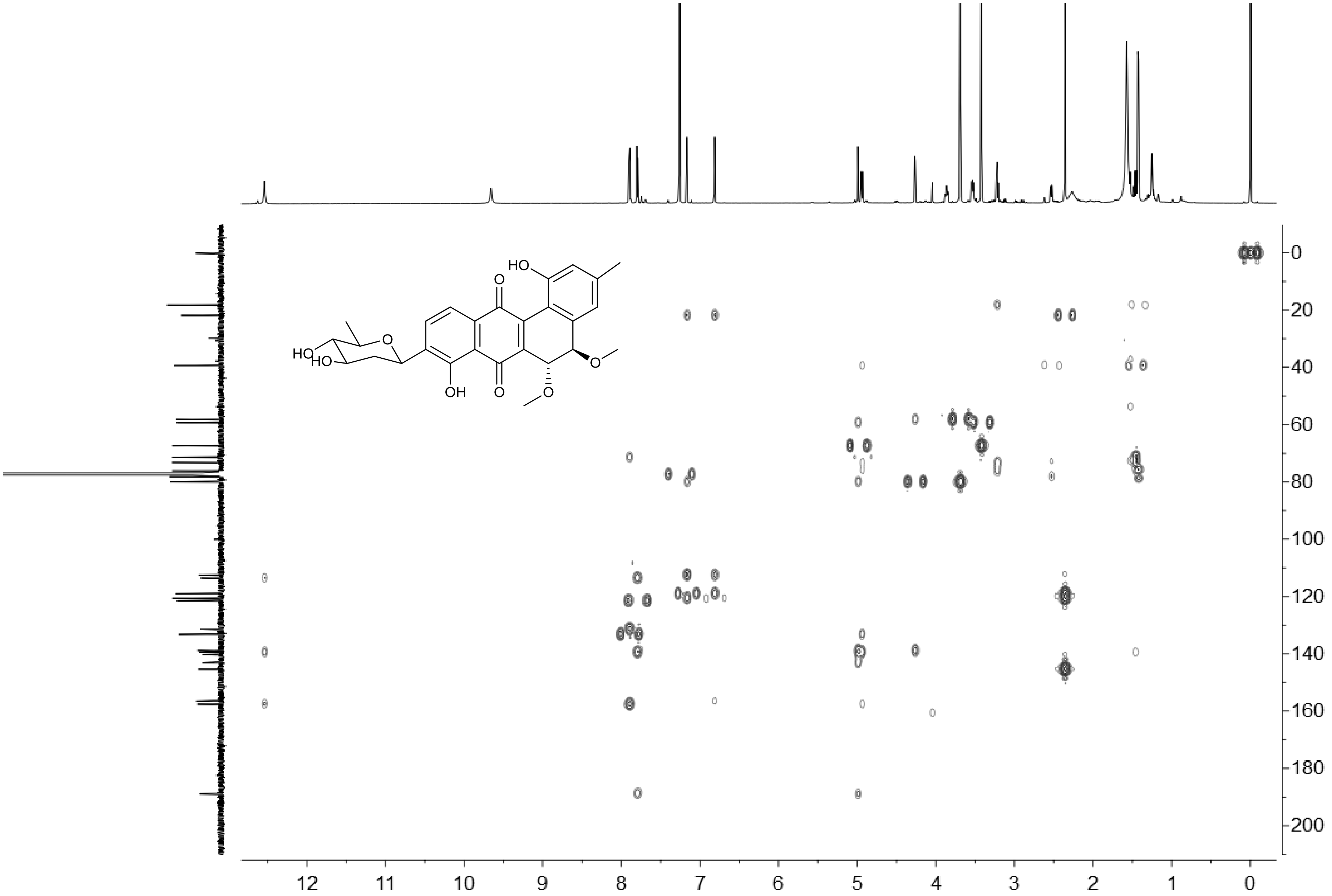

Figure S49. NOESY spectrum of compound **7** in $CDCl_3$.

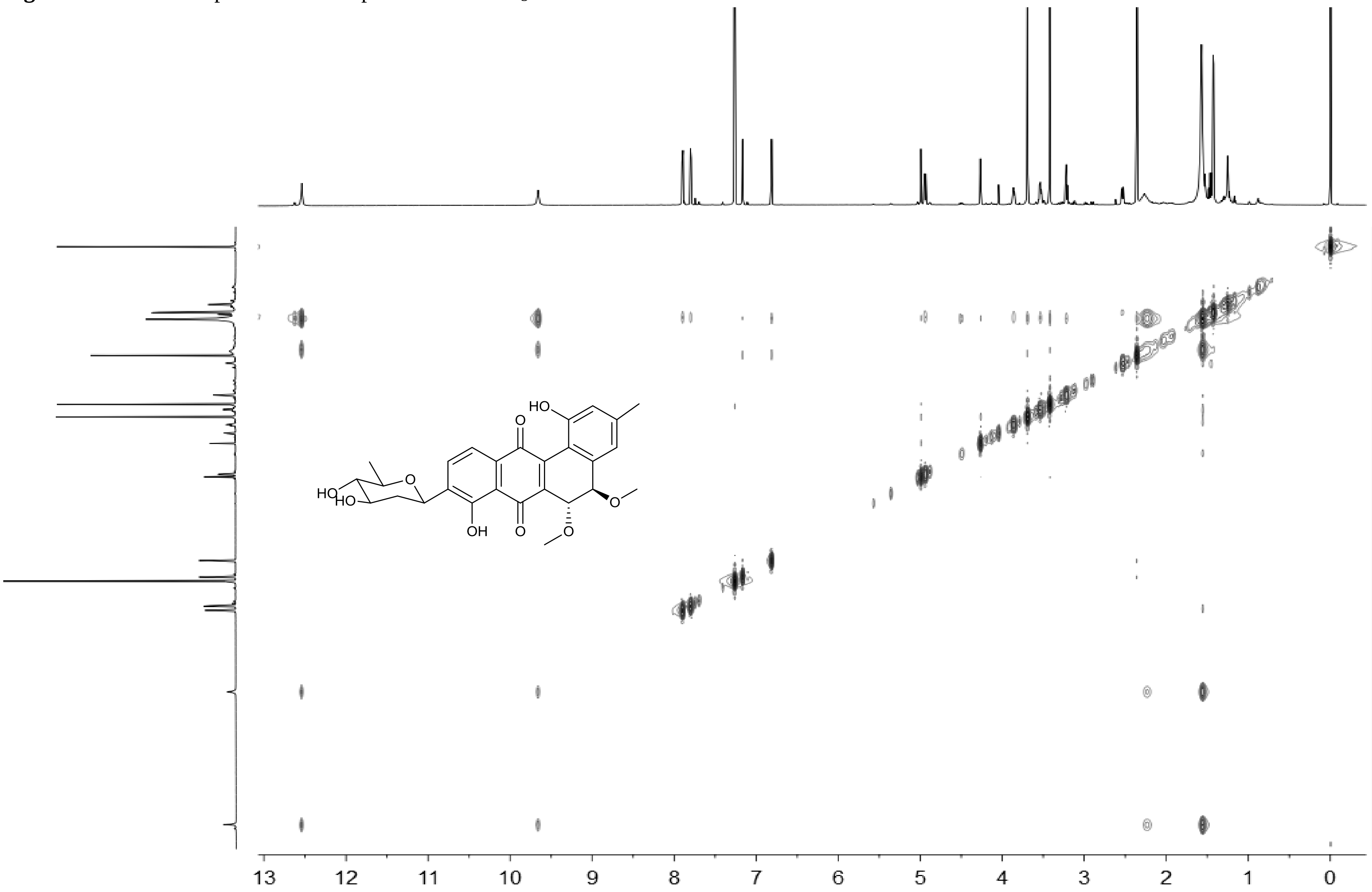

Figure S50. ^{1}H NMR (700 MHz) spectrum of compound **8** in $CDCl_3$.

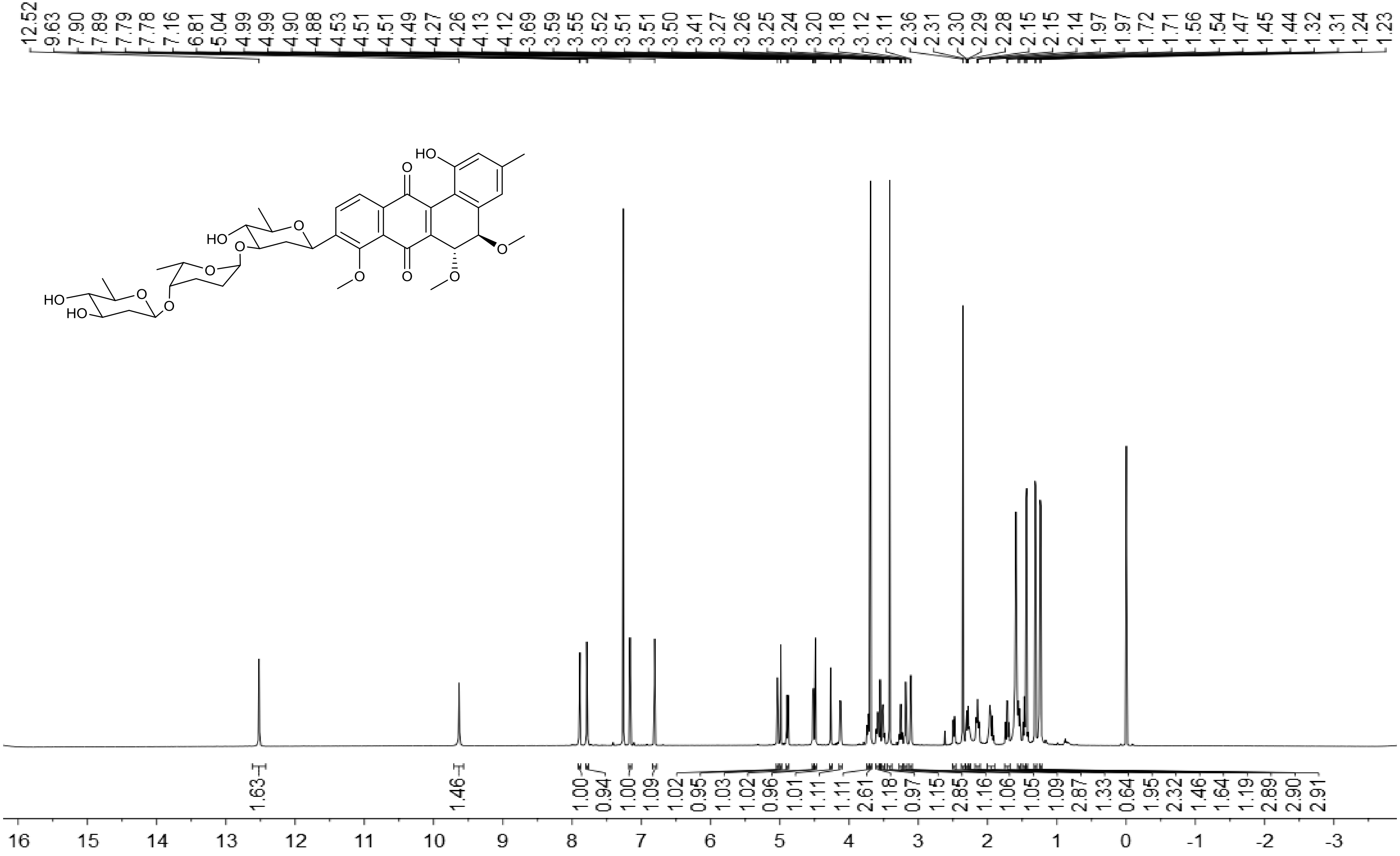

Figure S51. ^{13}C NMR (176 MHz) spectrum of compound **8** in $CDCl_3$.

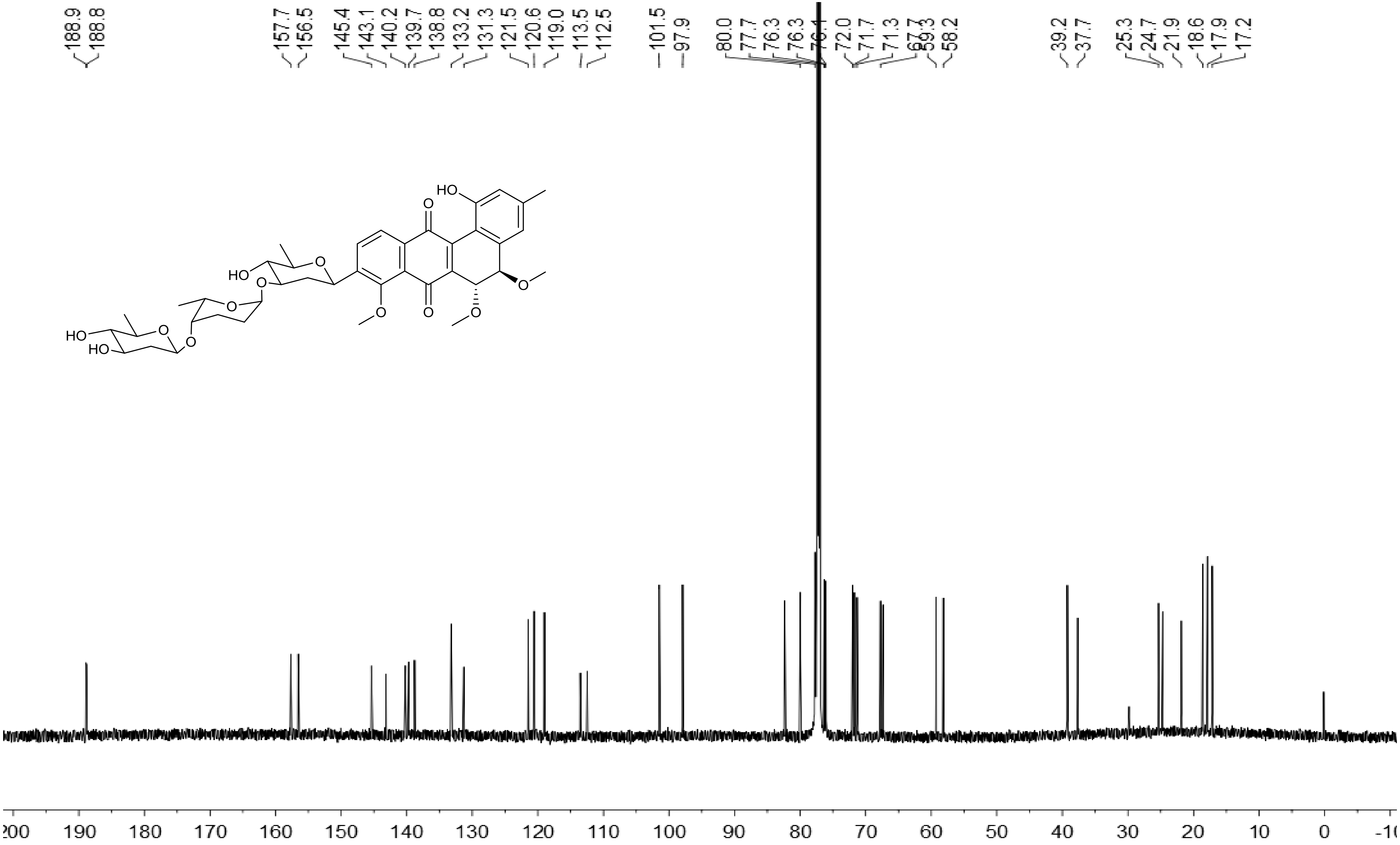

Figure S52. ^{13}C DEPT spectrum of compound **8** in $CDCl_3$.

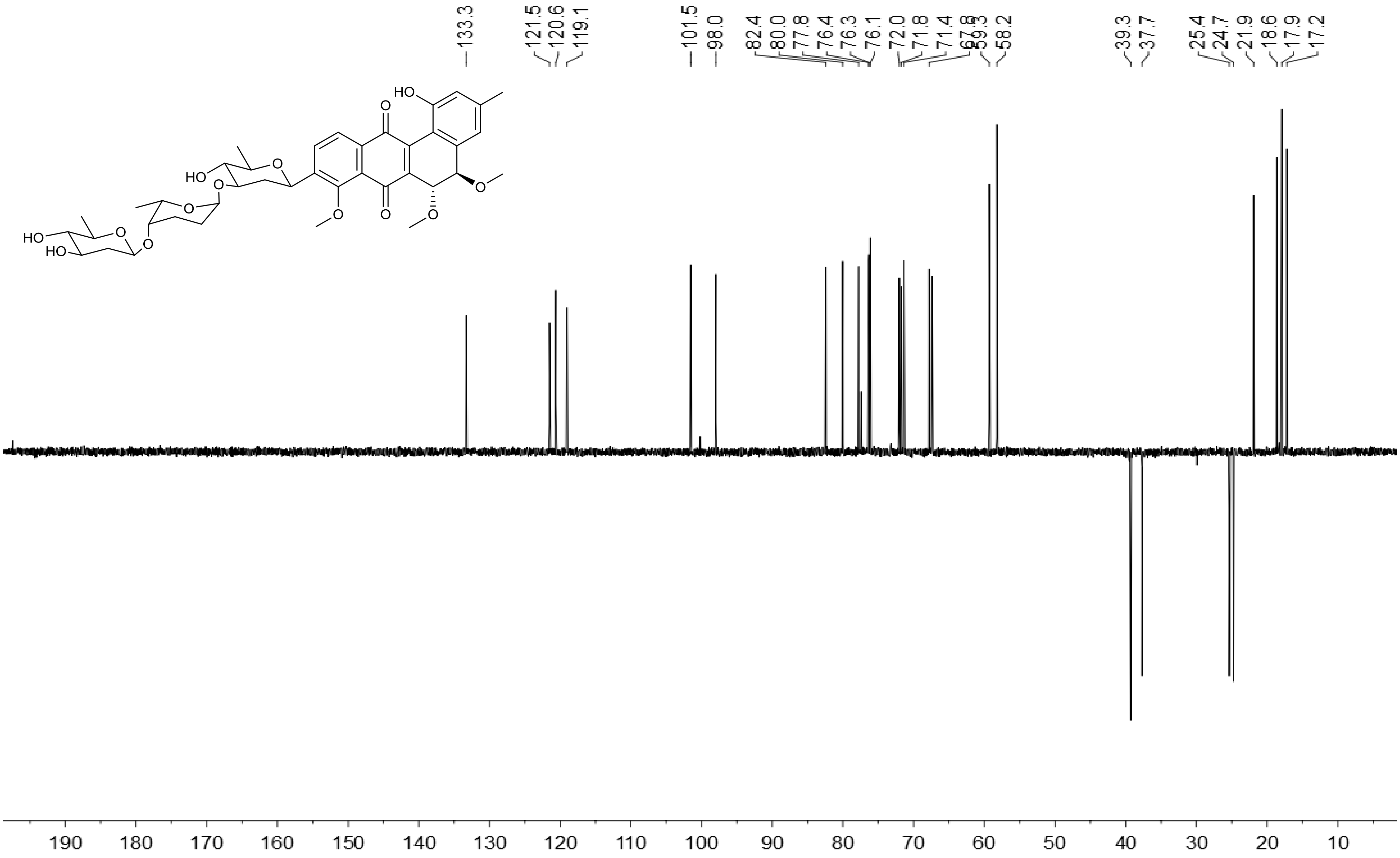

Figure S53. ^{1}H-^{1}H COSY spectrum of compound **8** in $CDCl_3$.

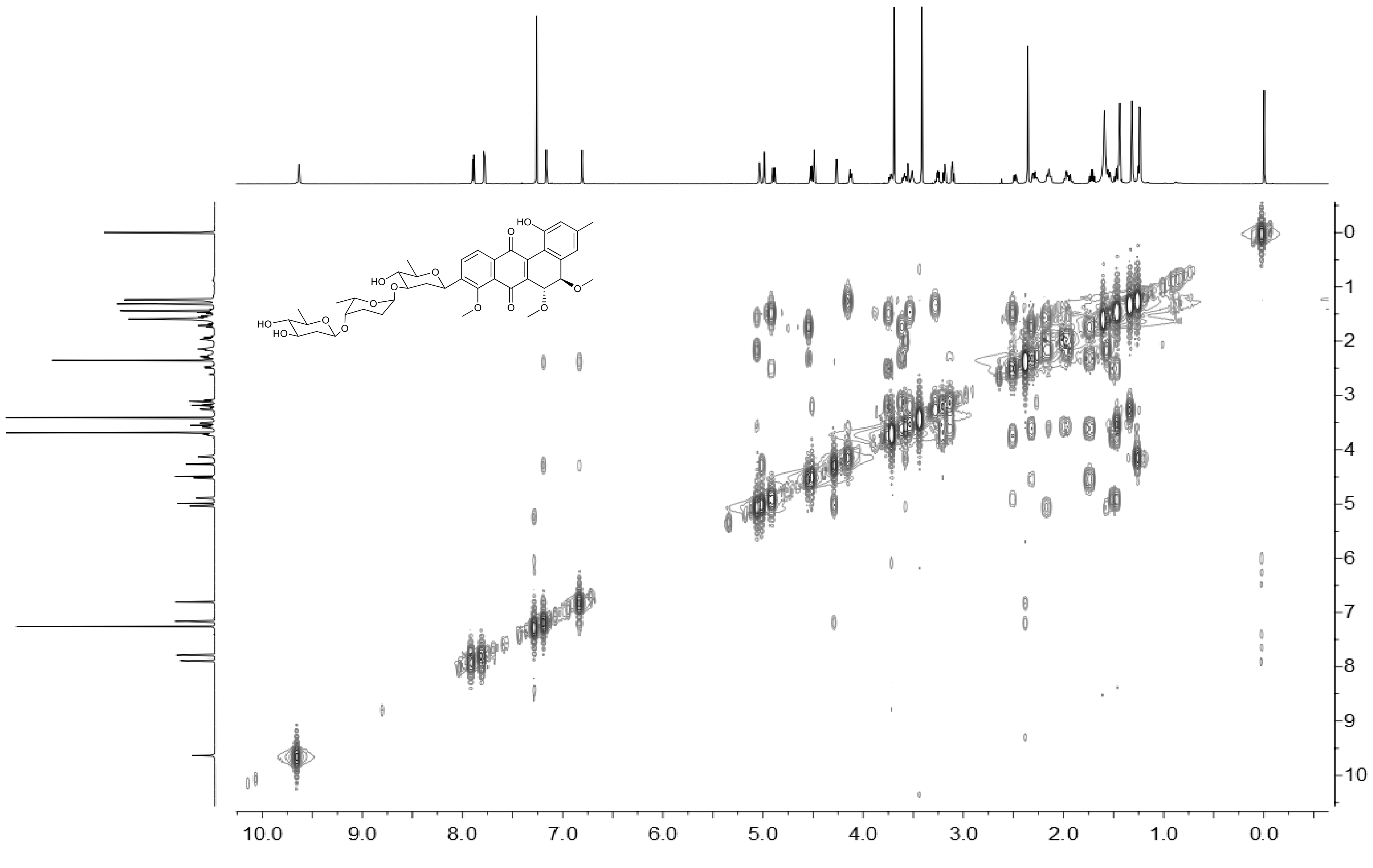

Figure S54. HSQC spectrum of compound **8** in $CDCl_3$.

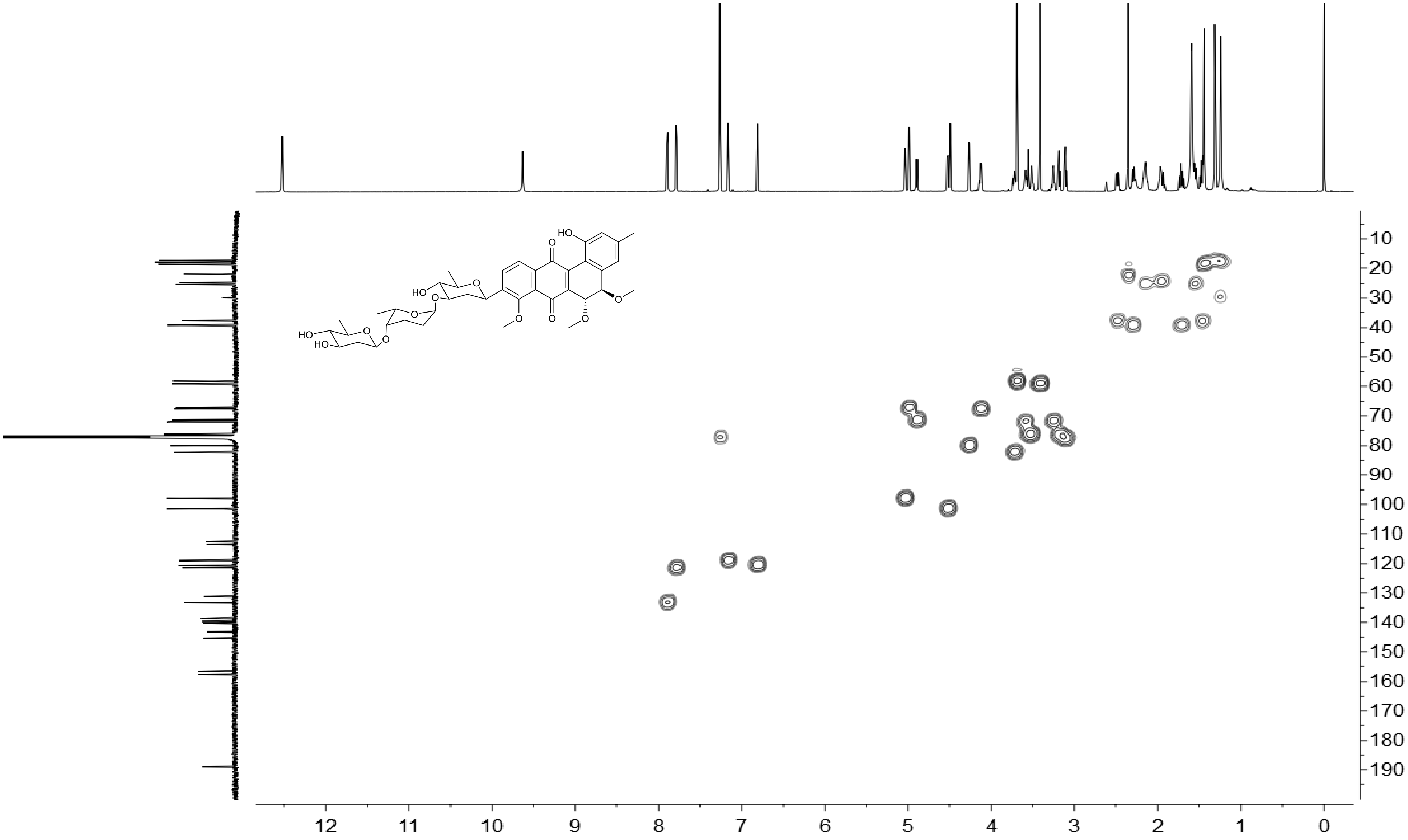

Figure S55. HMBC spectrum of compound **8** in $CDCl_3$.

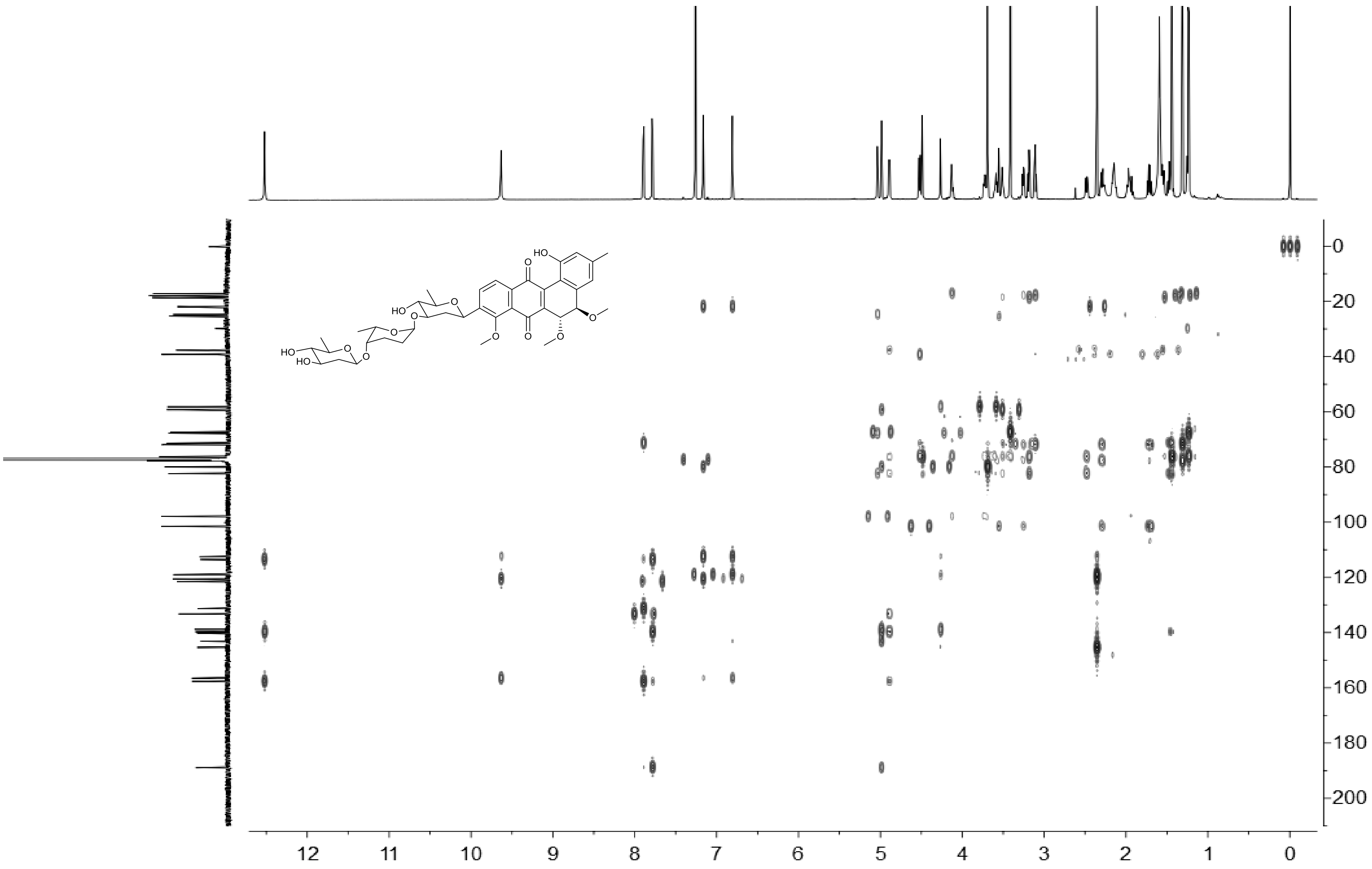

Figure S56. NOESY spectrum of compound **8** in $CDCl_3$.

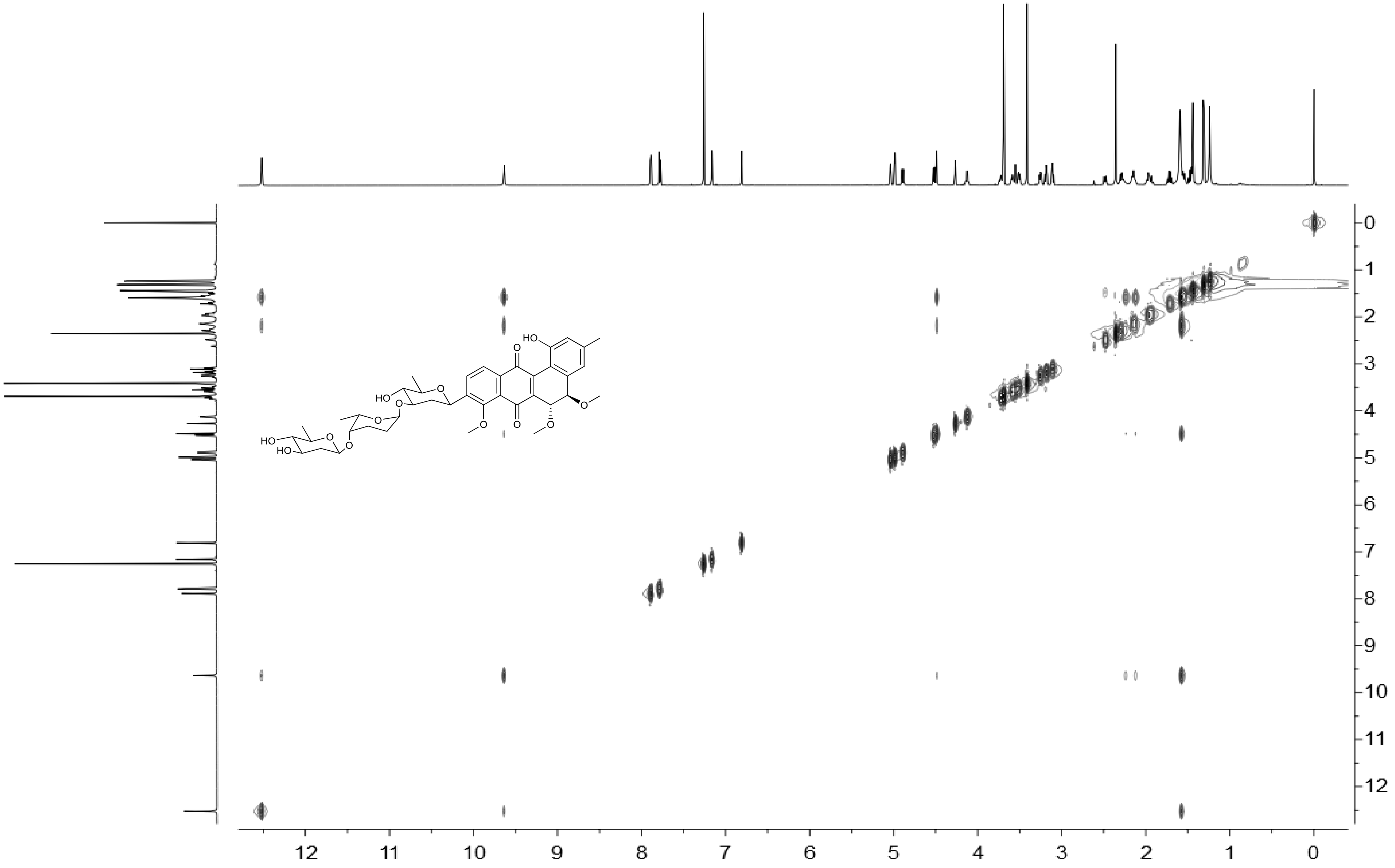

Figure S57. ^{1}H NMR (700 MHz) spectrum of compound **9** in $CDCl_3$.

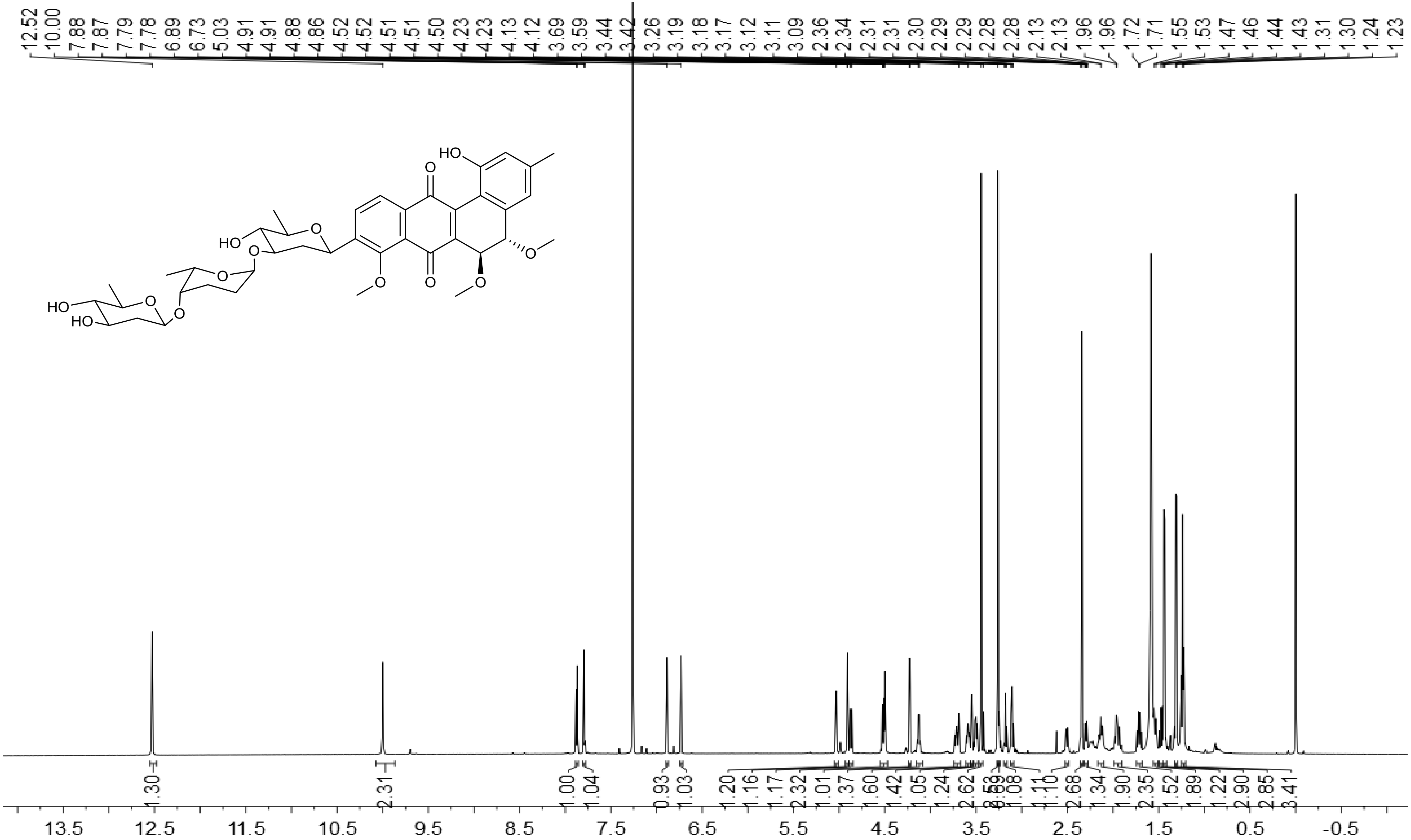

Figure S58. ^{13}C NMR (176 MHz) spectrum of compound **9** in $CDCl_3$.

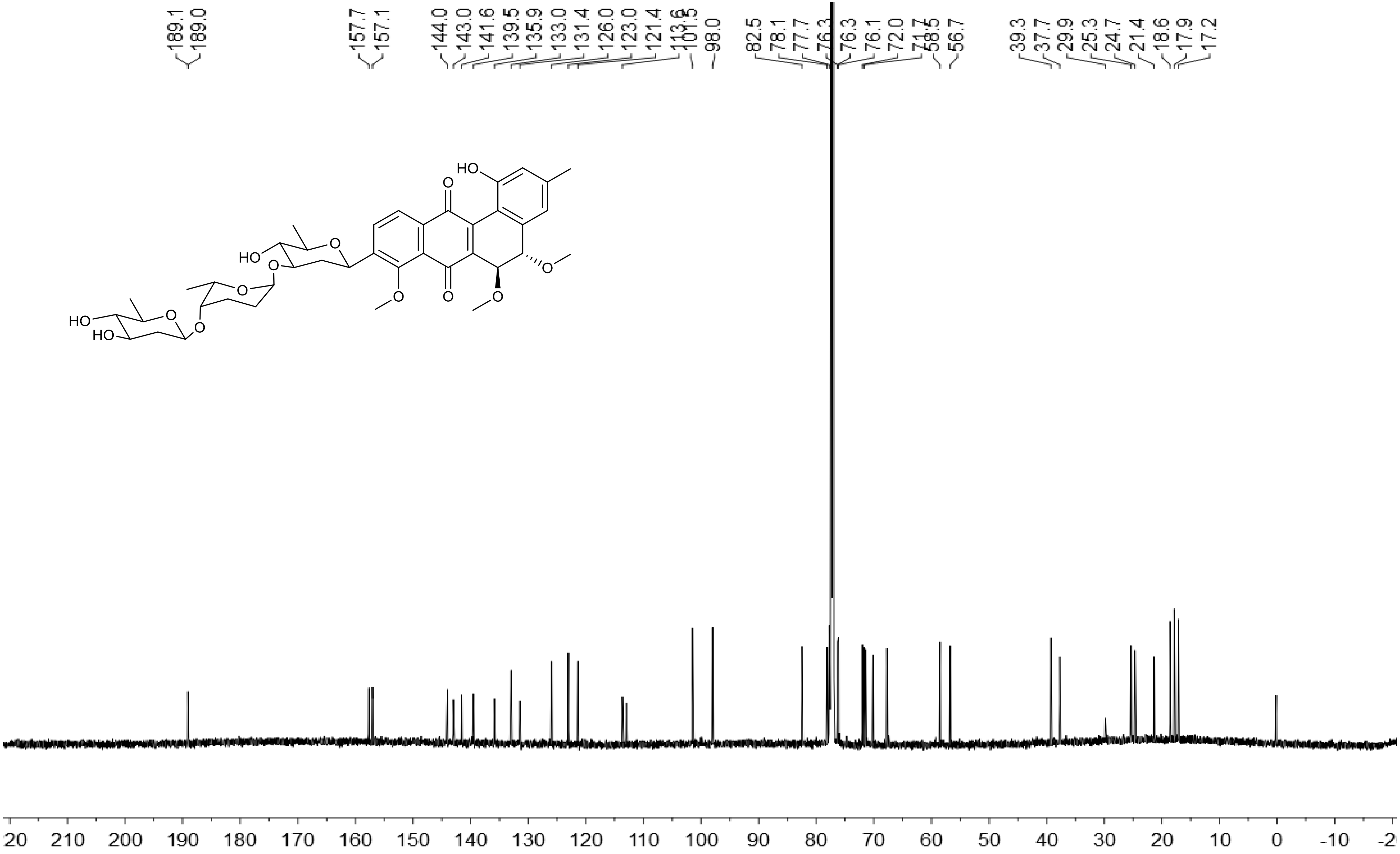

Figure S59. ^{13}C DEPT spectrum of compound **9** in $CDCl_3$.

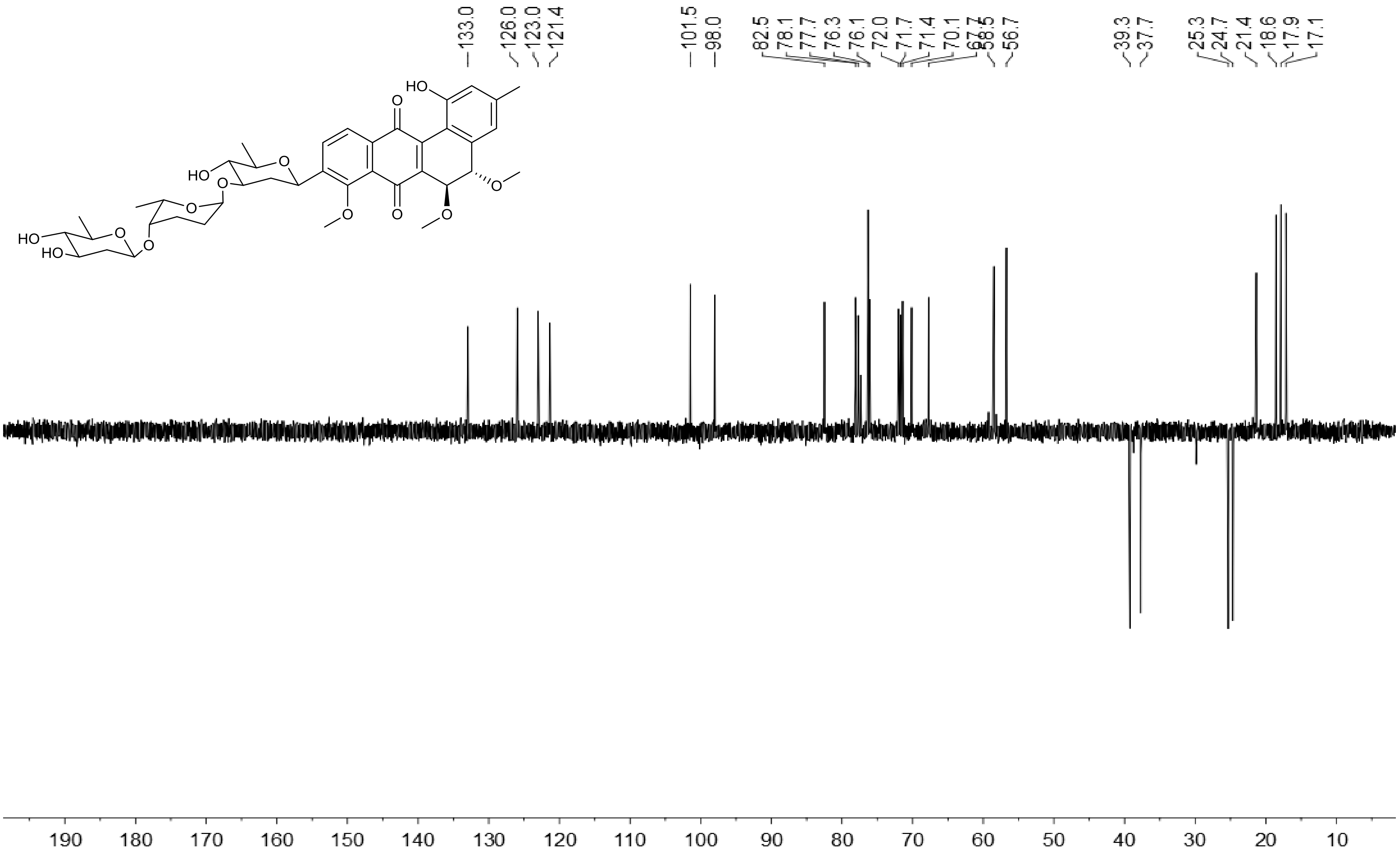

Figure S60. ^{1}H-^{1}H COSY spectrum of compound **9** in $CDCl_3$.

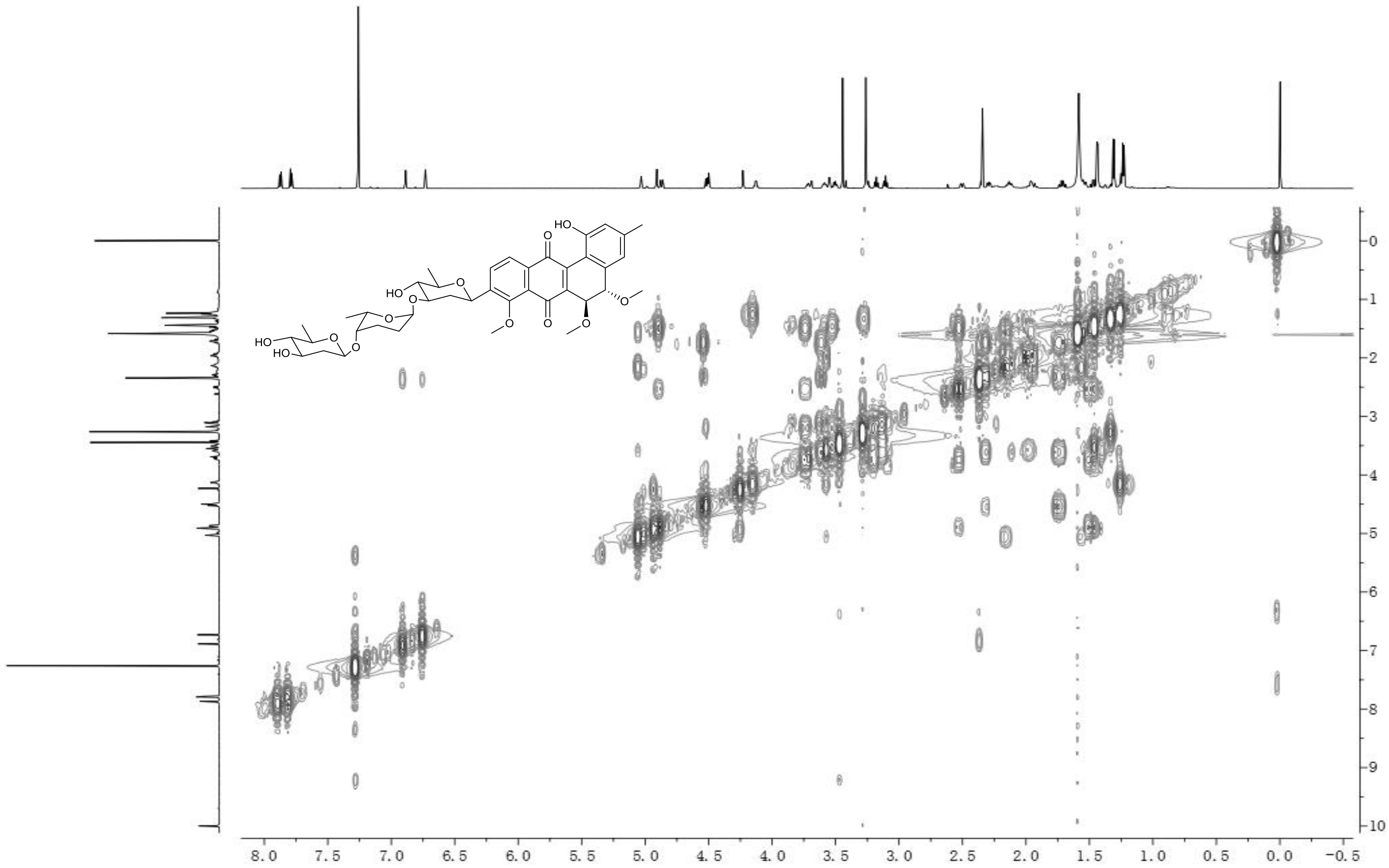

Figure S61. HSQC spectrum of compound **9** in $CDCl_3$.

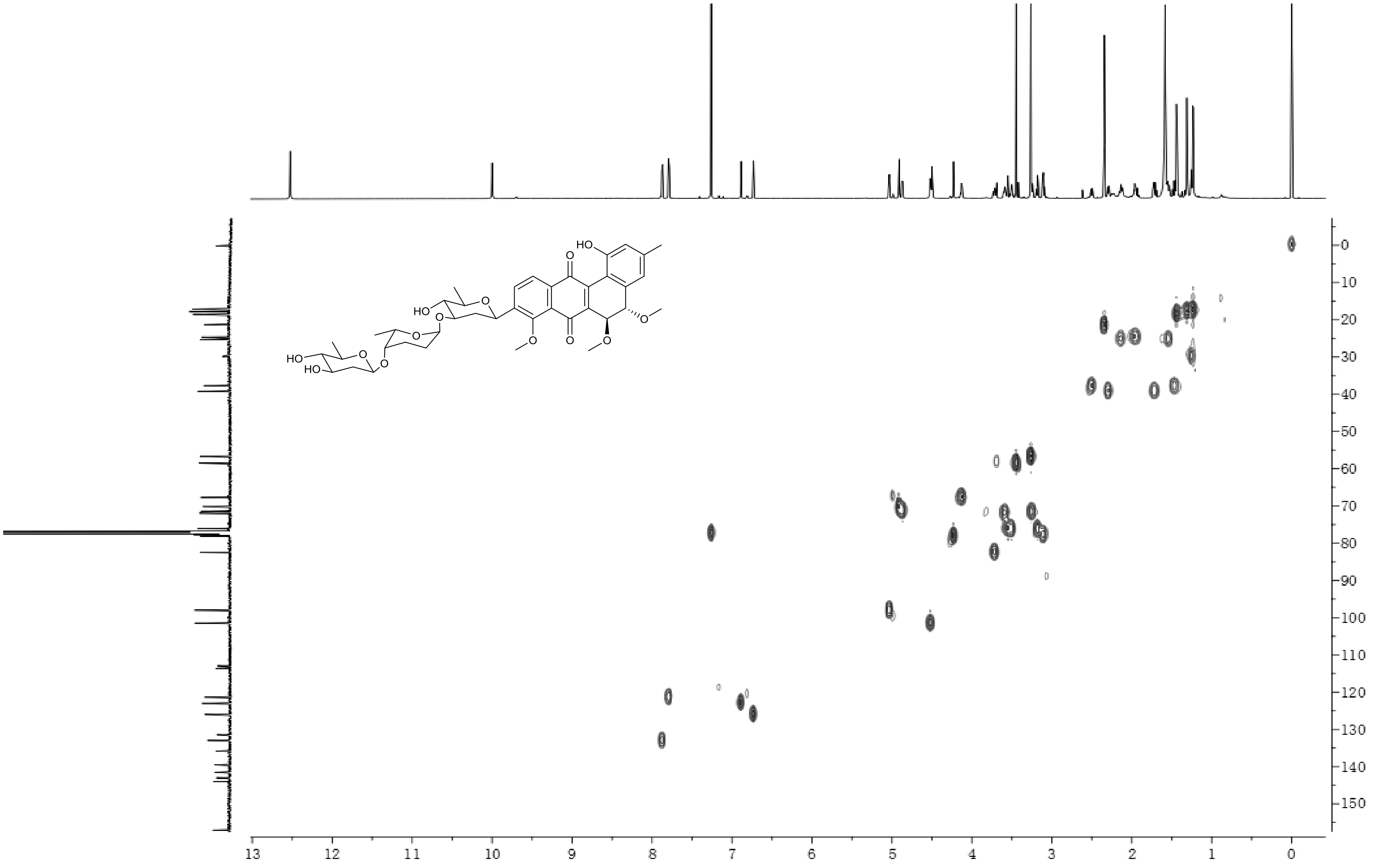

Figure S62. HMBC spectrum of compound **9** in $CDCl_3$.

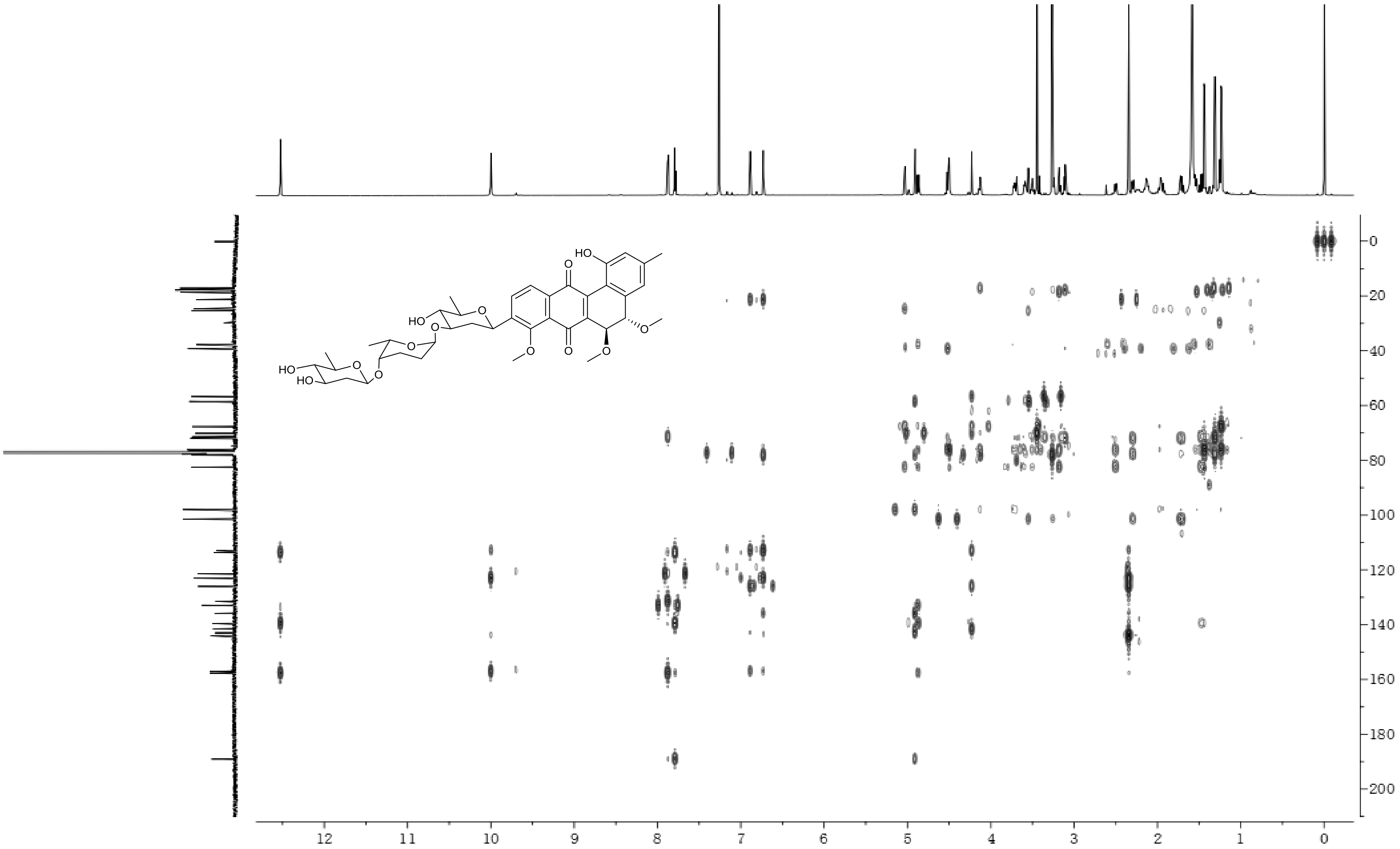

Figure S63. NOESY spectrum of compound **9** in $CDCl_3$.

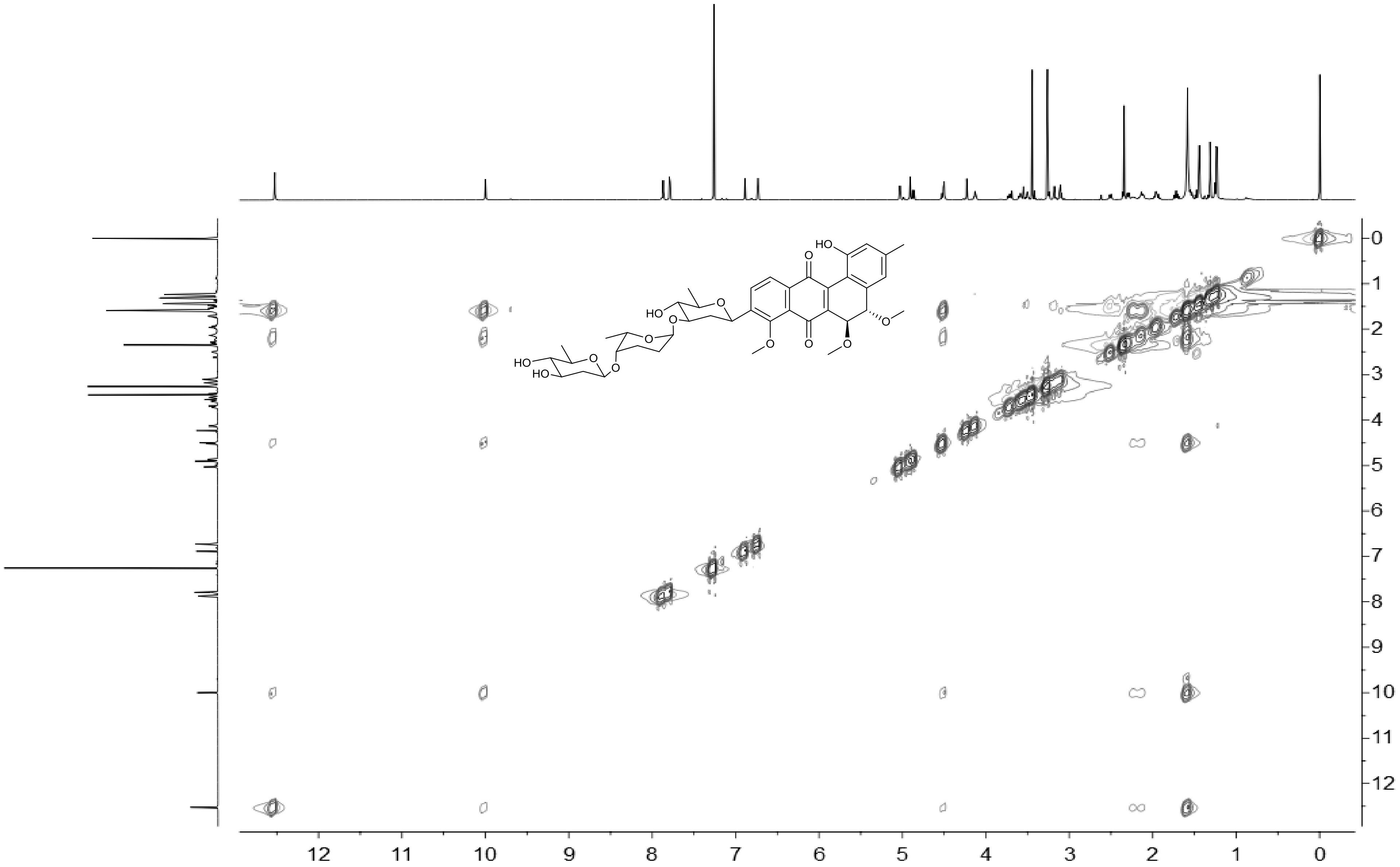

Supplement: Supplementary file 1 [file marinedrugs-16-00185-s001.pdf]
